# Supplementary figures and images for: Acclimation and degradation characteristic of the microbial system in corn straw (part 1 of 2)
Source: PeerJ. 2025 Dec 16;13:e20386. doi: 10.7717/peerj.20386 (PMC12716131; doi:10.7717/peerj.20386)

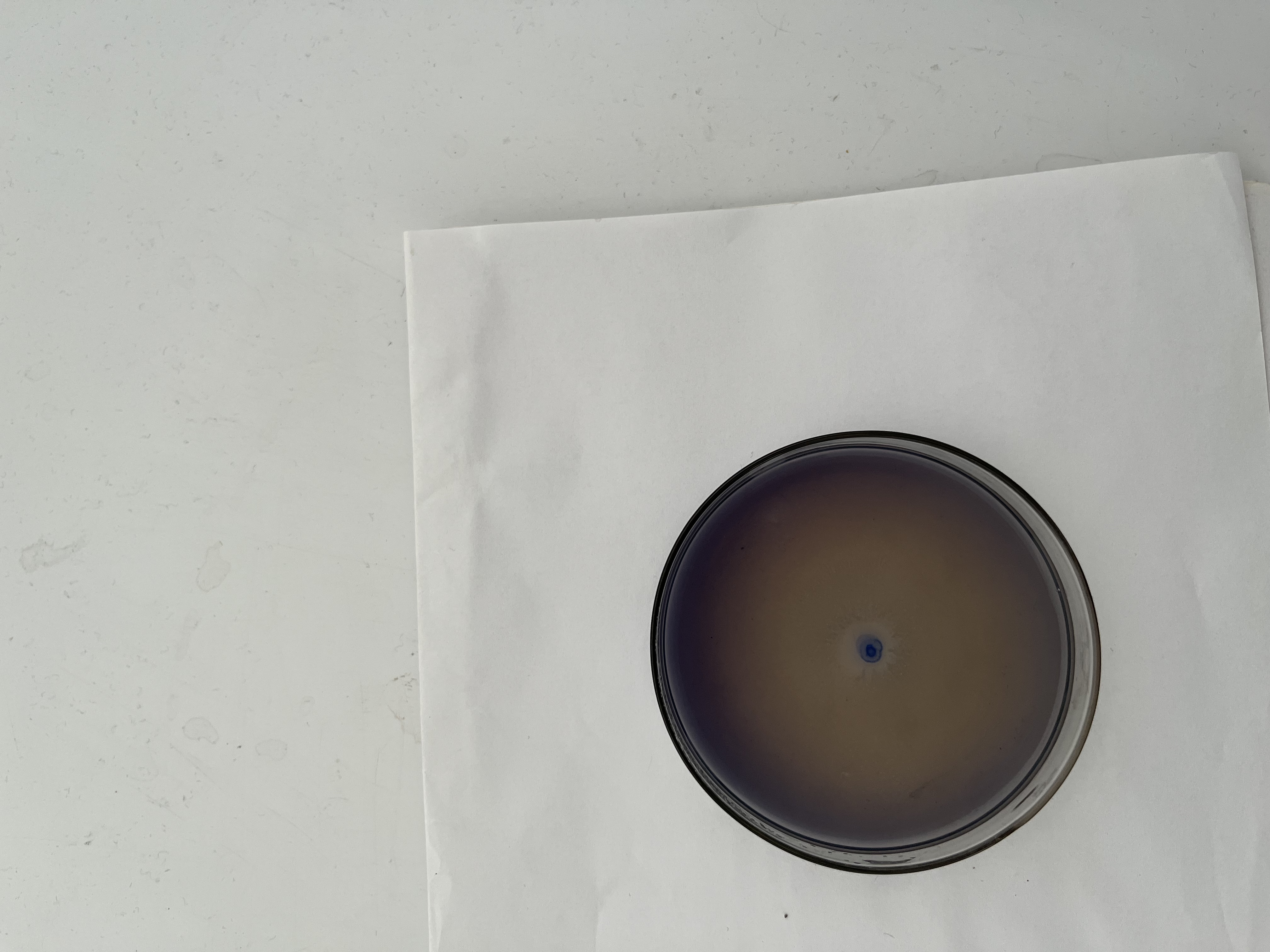

Supplement: Supplemental Information 1 [file peerj-13-20386-s001.zip › Raw data1 Congo red and aniline blue staining results/0d0bb4ba849eefa8080adfdc6bf407e.jpg]

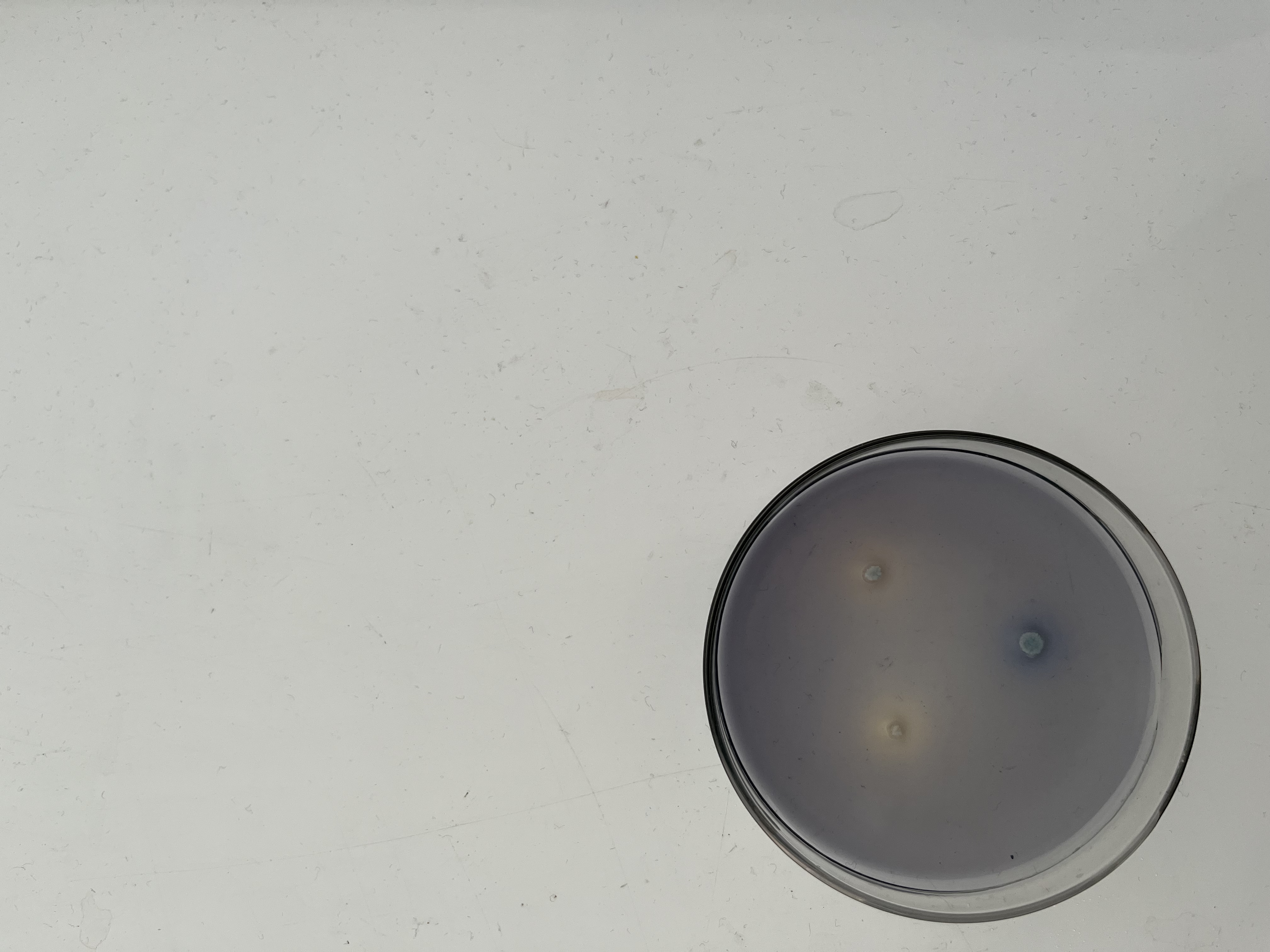

Supplement: Supplemental Information 1 [file peerj-13-20386-s001.zip › Raw data1 Congo red and aniline blue staining results/36b05969c0baceb22ae3333ee1bf76d.jpg]

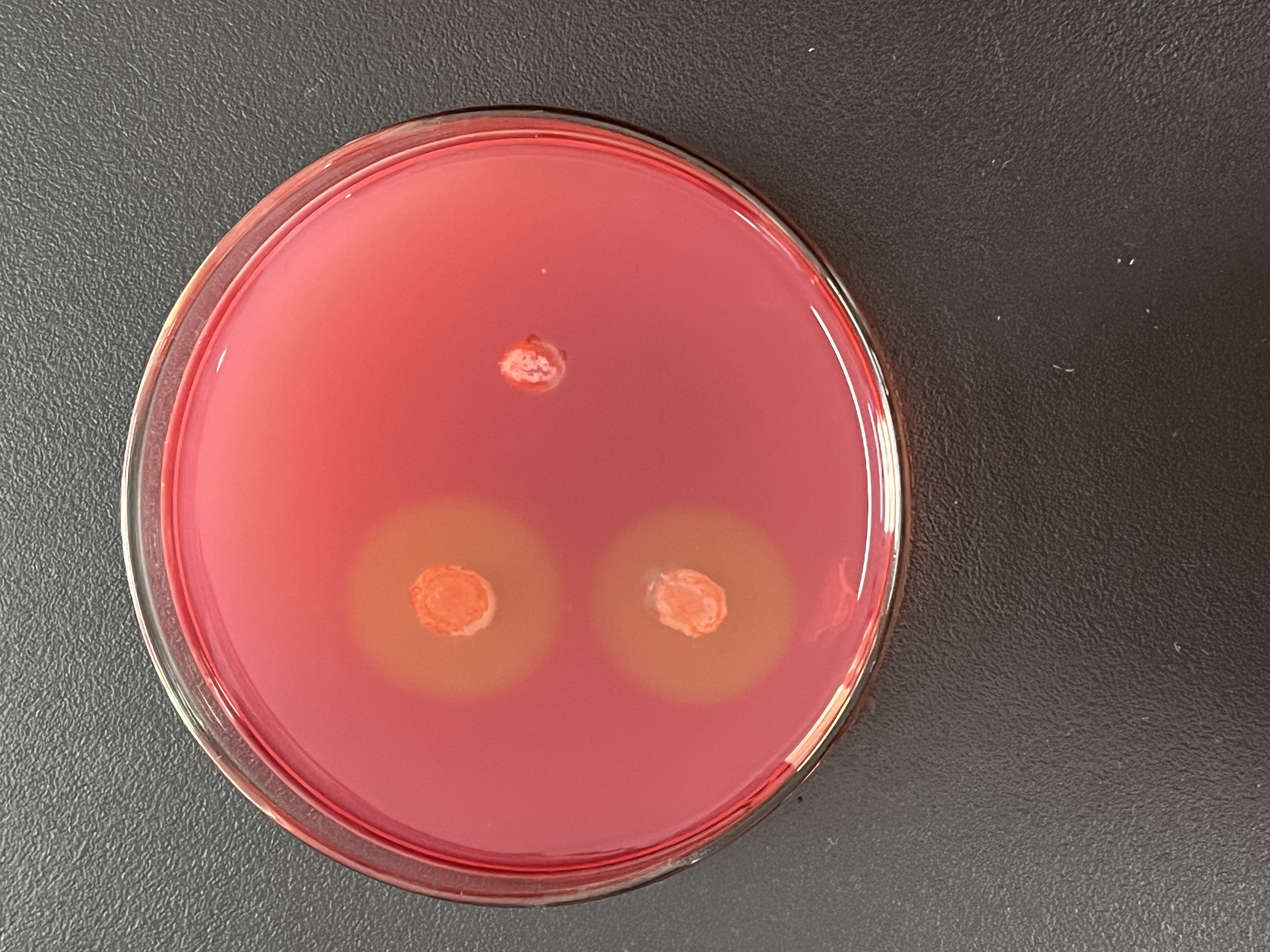

Supplement: Supplemental Information 1 [file peerj-13-20386-s001.zip › Raw data1 Congo red and aniline blue staining results/8c43599f37f34adcda790240aded28c.jpg]

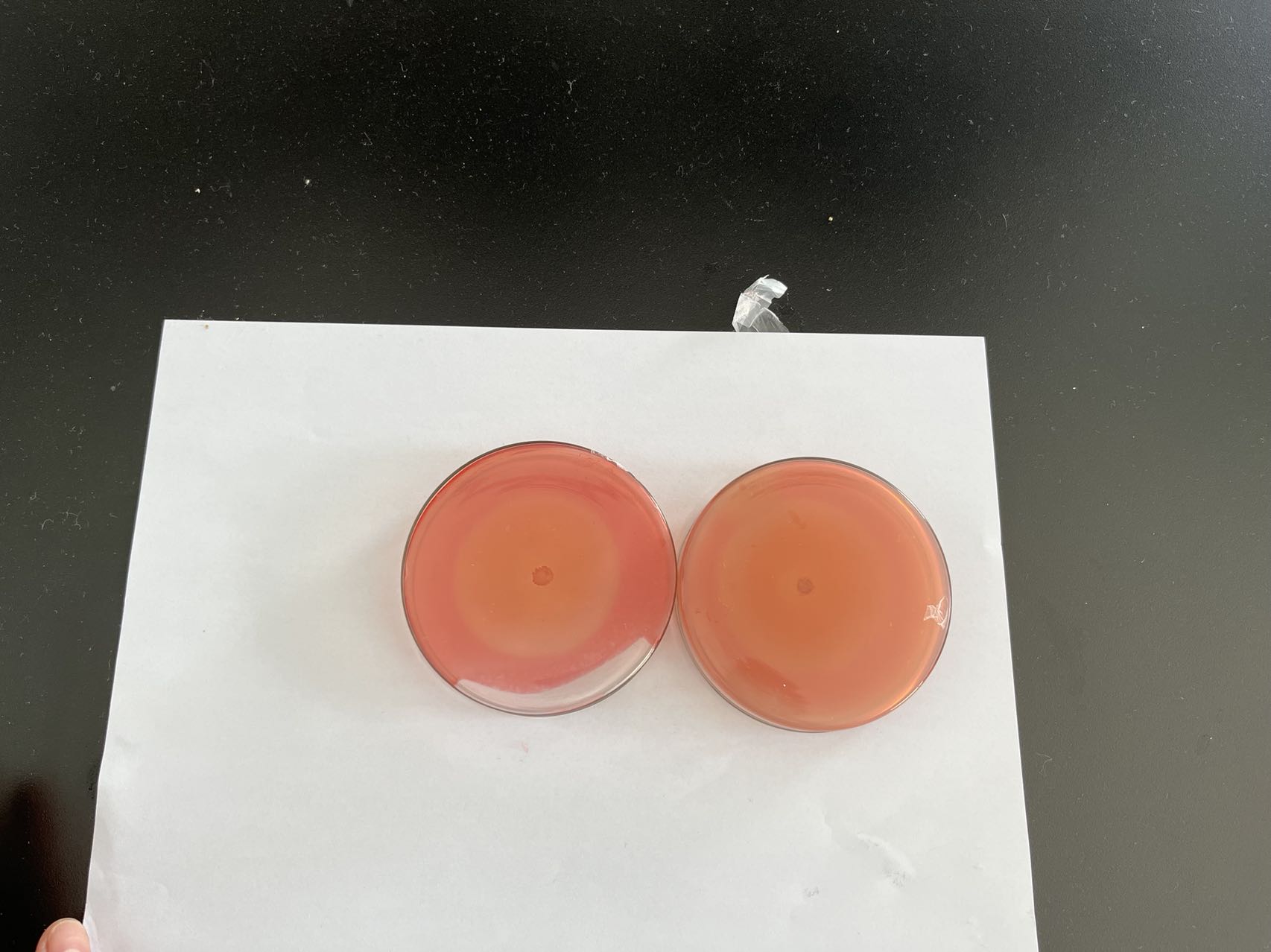

Supplement: Supplemental Information 1 [file peerj-13-20386-s001.zip › Raw data1 Congo red and aniline blue staining results/dac0a838e561a3a621d1b26cacc3261.jpg]

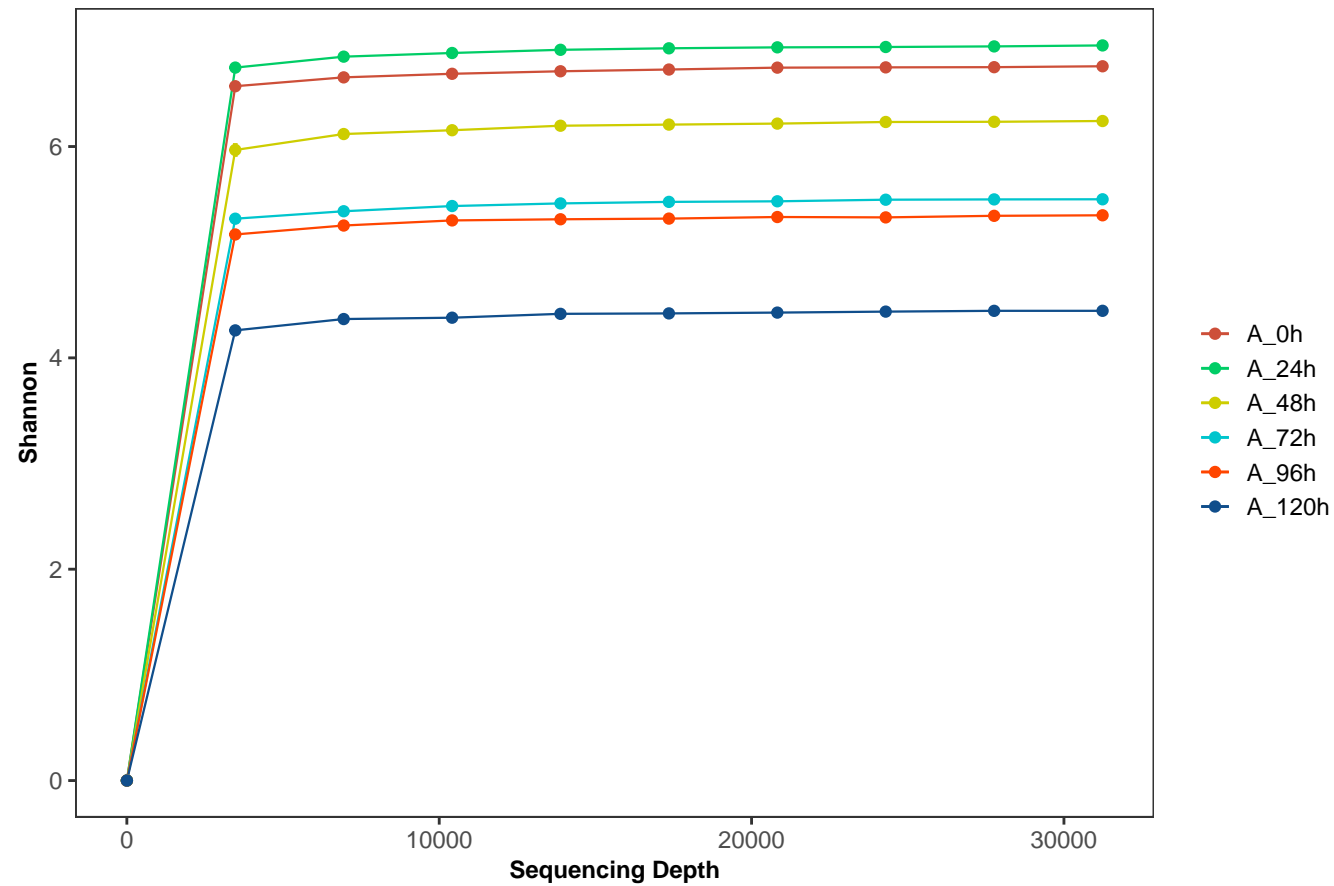

Supplement: Supplemental Information 3 [file peerj-13-20386-s003.zip › Raw data 3 Structural of microbial communities/24 48Shannon_rare_curve_errorbar.group.pdf]

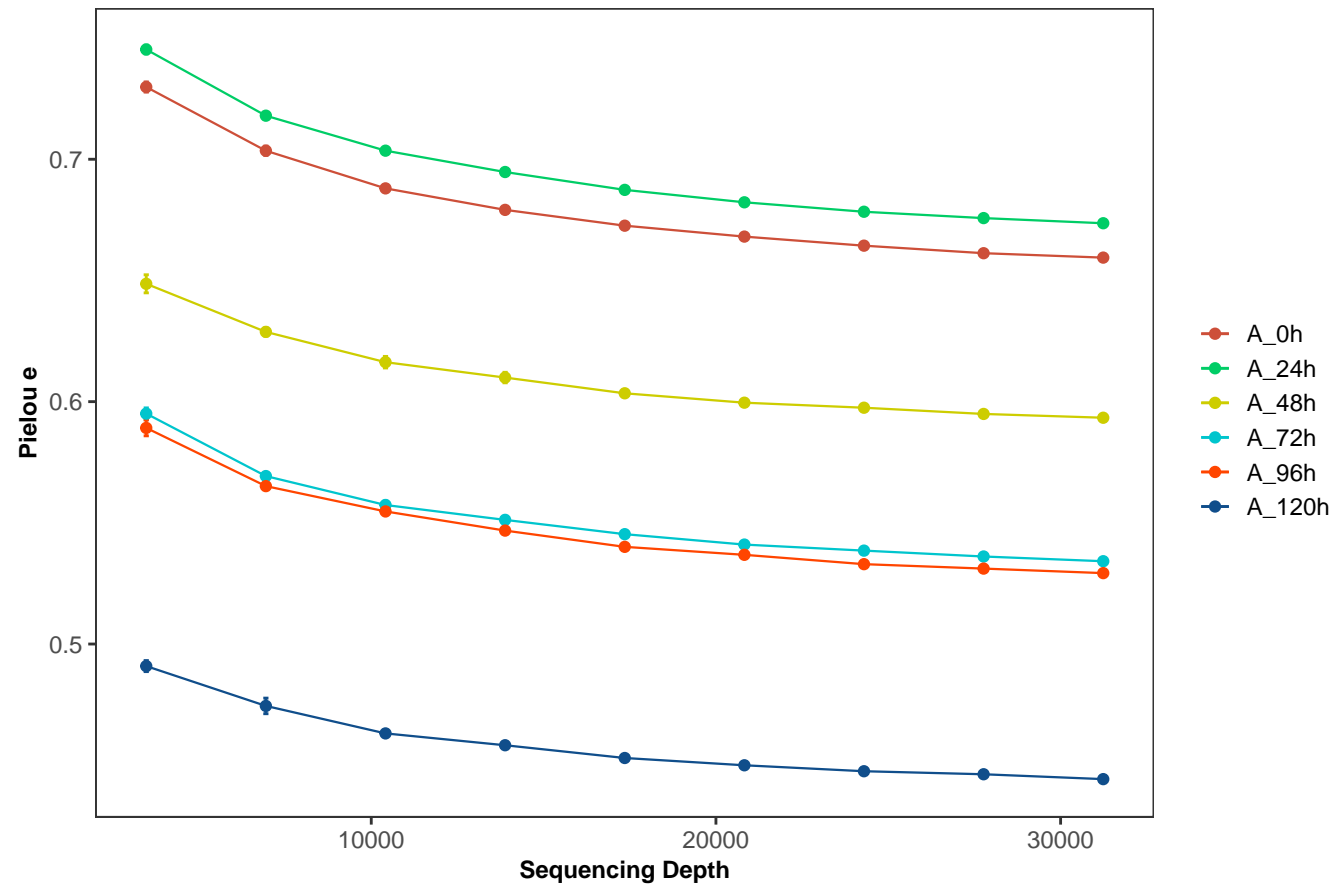

Supplement: Supplemental Information 3 [file peerj-13-20386-s003.zip › Raw data 3 Structural of microbial communities/24 Pielou_e_rare_curve_errorbar.group.pdf]

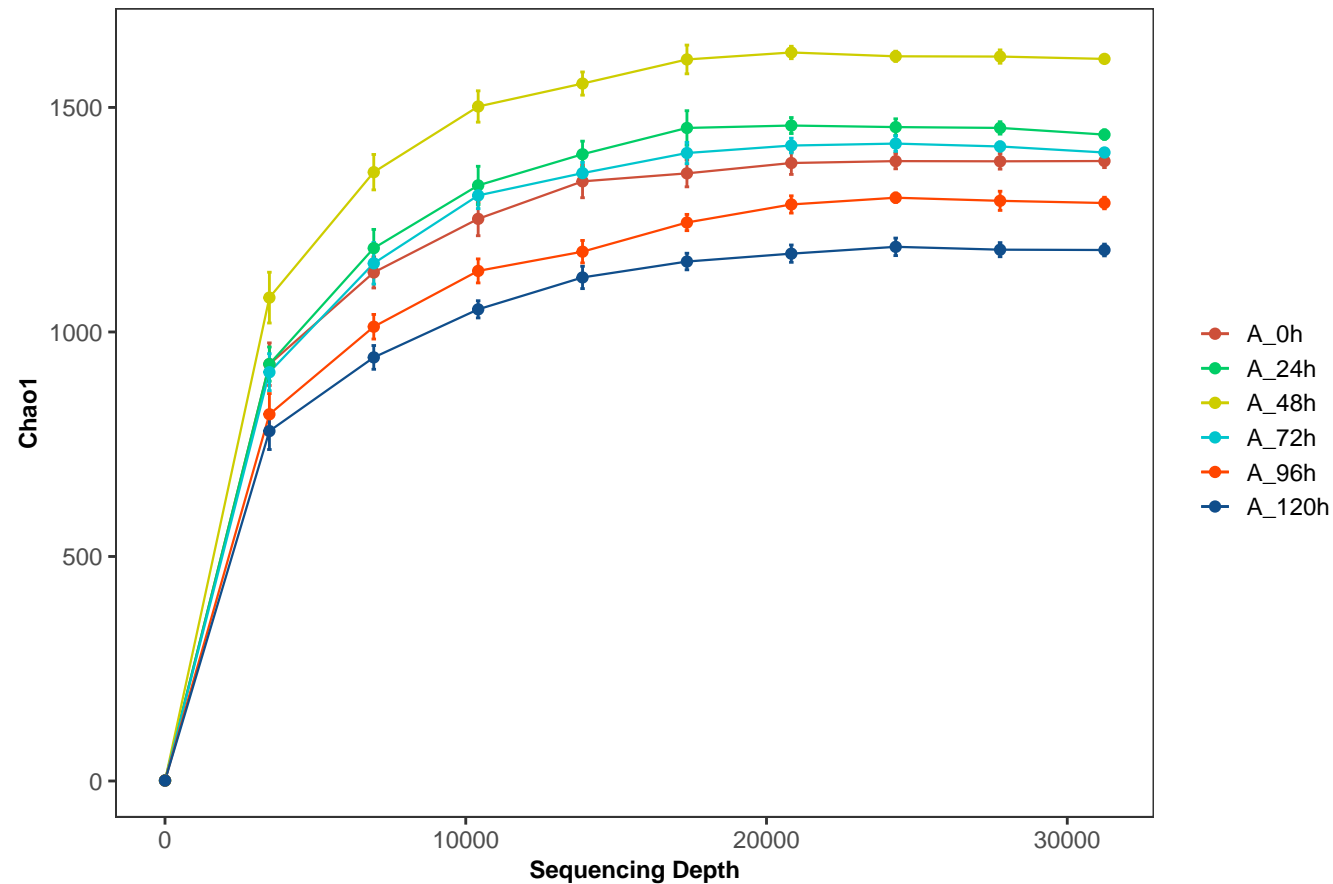

Supplement: Supplemental Information 3 [file peerj-13-20386-s003.zip › Raw data 3 Structural of microbial communities/48Chao1_rare_curve_errorbar.group.pdf]

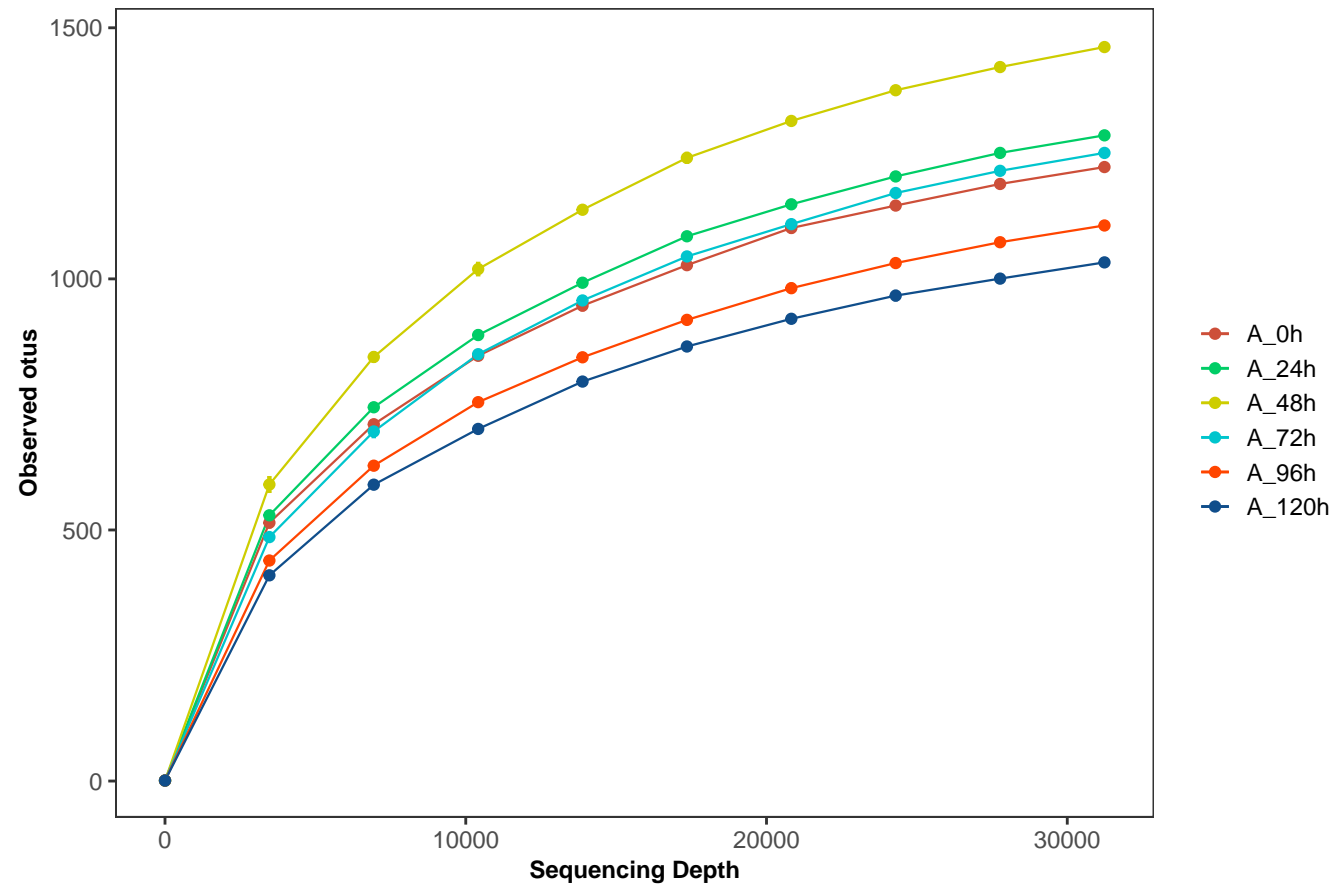

Supplement: Supplemental Information 3 [file peerj-13-20386-s003.zip › Raw data 3 Structural of microbial communities/48Observed_otus_rare_curve_errorbar.group.pdf]

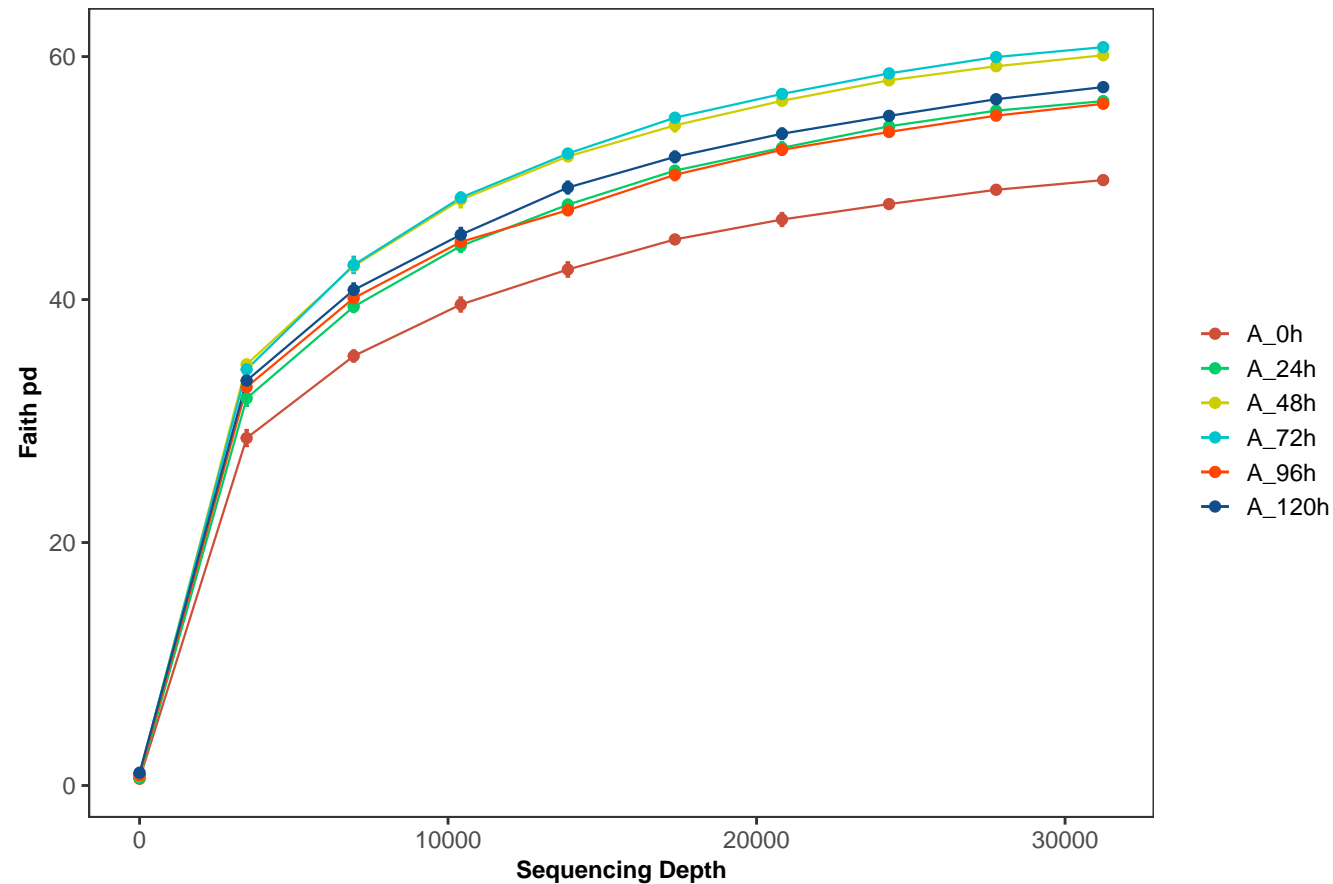

Supplement: Supplemental Information 3 [file peerj-13-20386-s003.zip › Raw data 3 Structural of microbial communities/72 48 Faith_pd_rare_curve_errorbar.group.pdf]

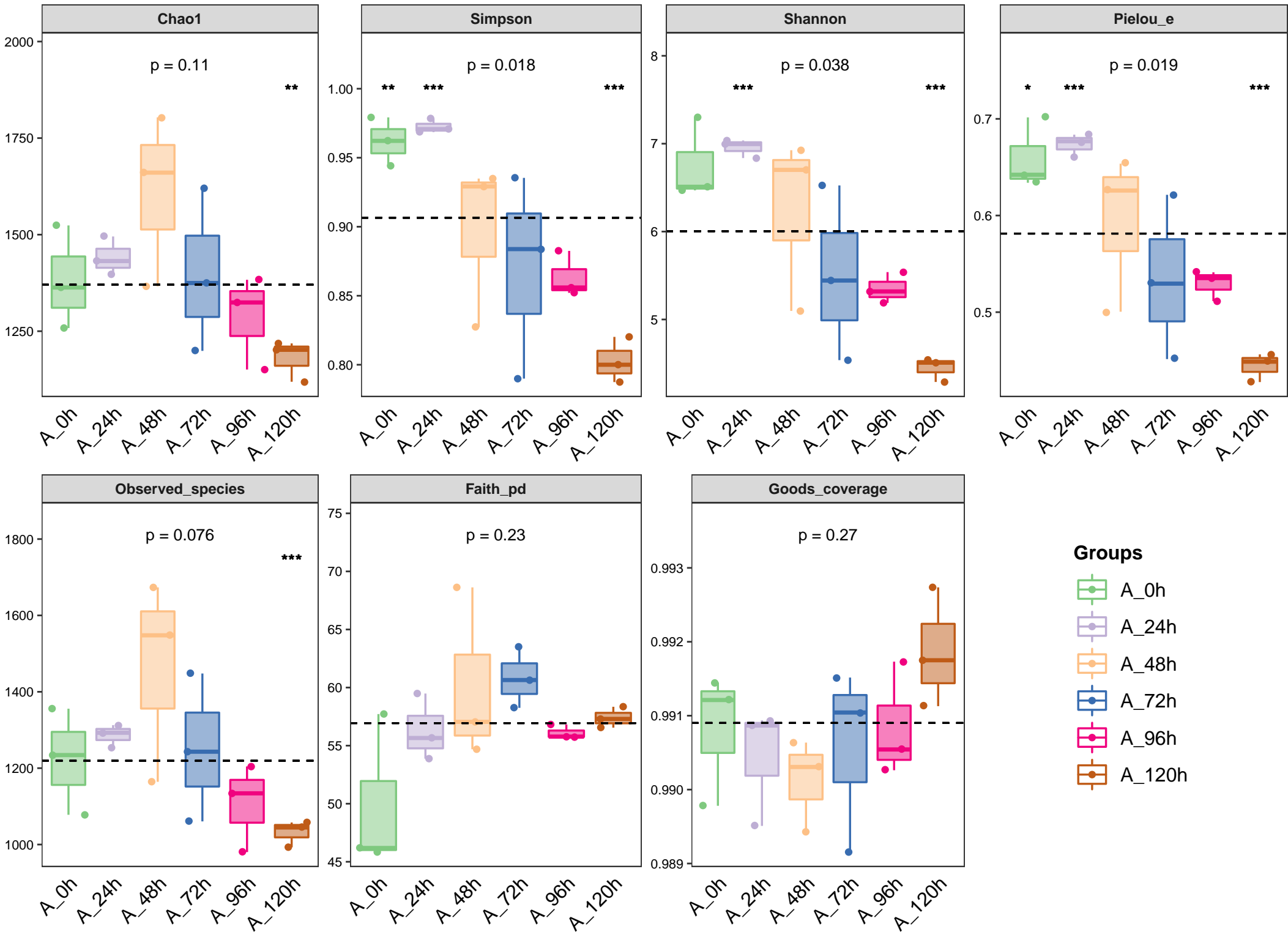

Supplement: Supplemental Information 3 [file peerj-13-20386-s003.zip › Raw data 3 Structural of microbial communities/alpha_boxplot.pdf]

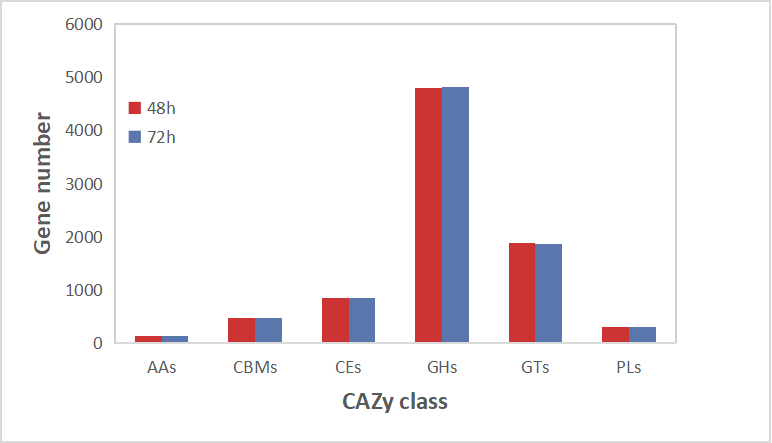

Supplement: Supplemental Information 3 [file peerj-13-20386-s003.zip › Raw data 3 Structural of microbial communities/barplot.png]

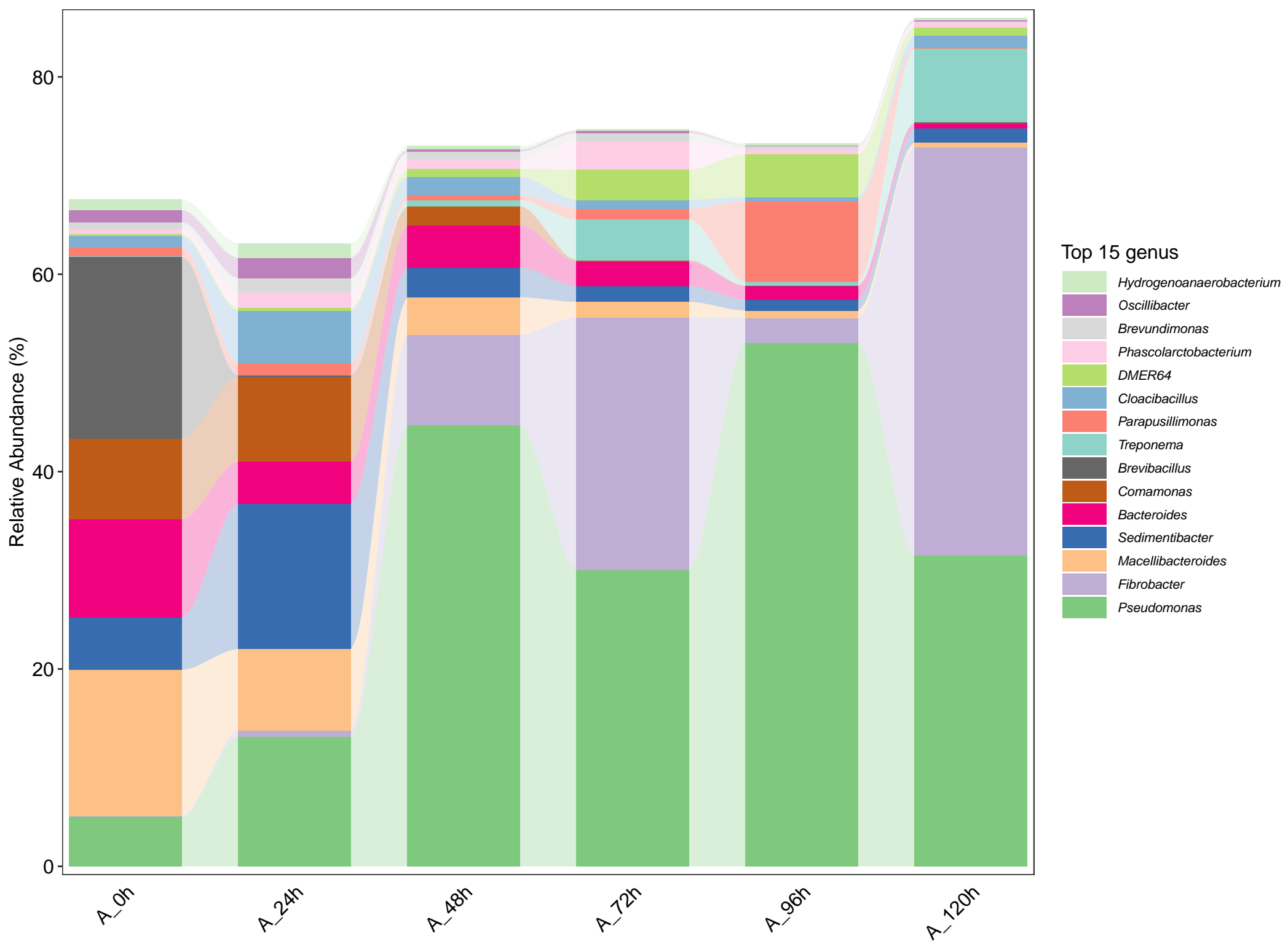

Supplement: Supplemental Information 3 [file peerj-13-20386-s003.zip › Raw data 3 Structural of microbial communities/bar_genus.pdf]

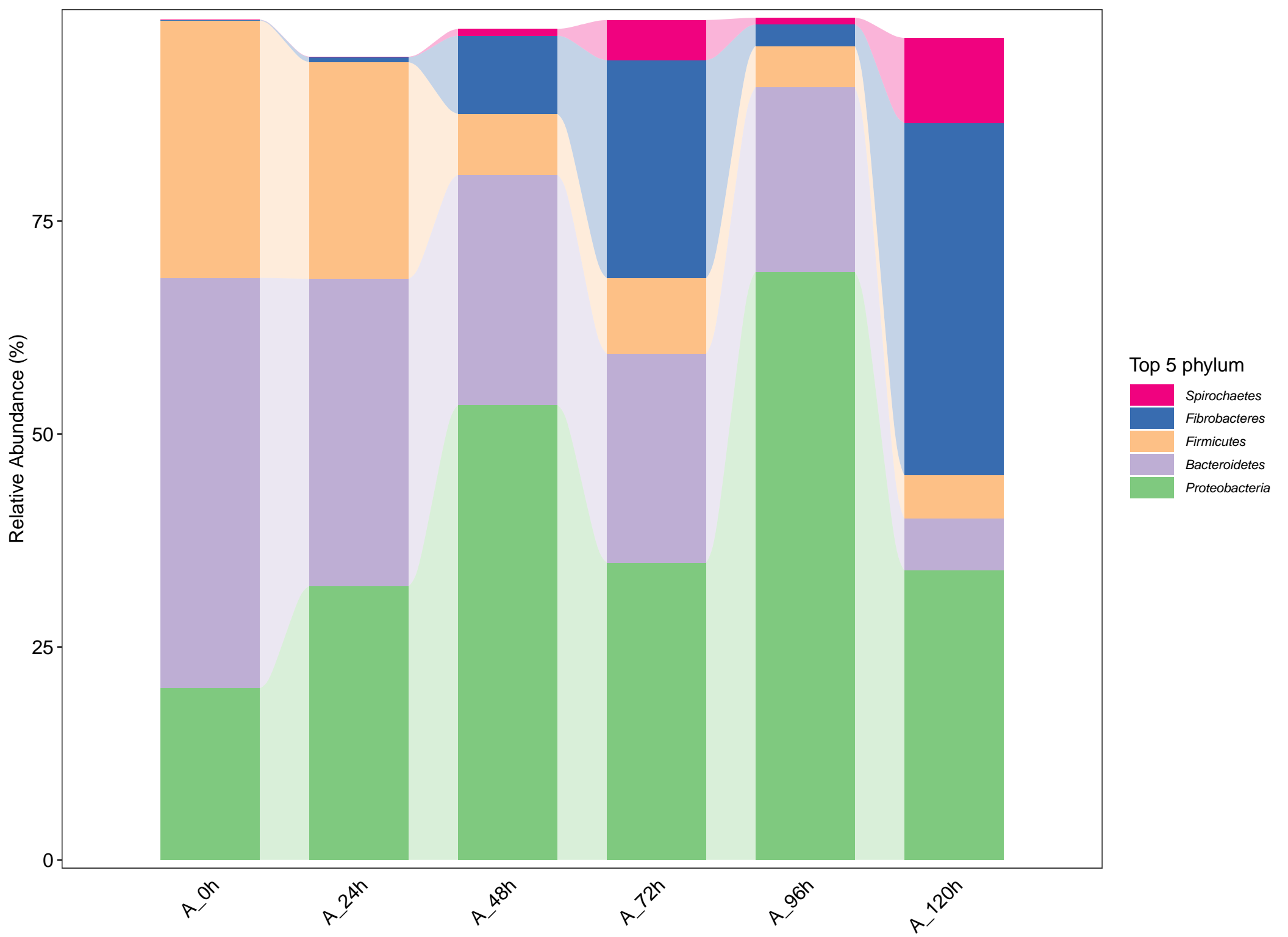

Supplement: Supplemental Information 3 [file peerj-13-20386-s003.zip › Raw data 3 Structural of microbial communities/bar_phylum.pdf]

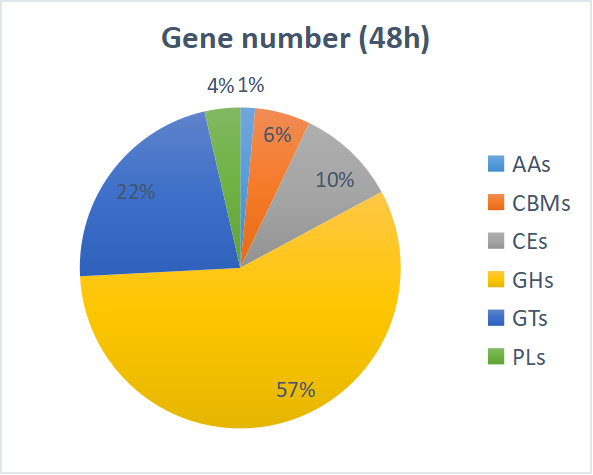

Supplement: Supplemental Information 3 [file peerj-13-20386-s003.zip › Raw data 3 Structural of microbial communities/CAZy.class.geneNum.48h.png]

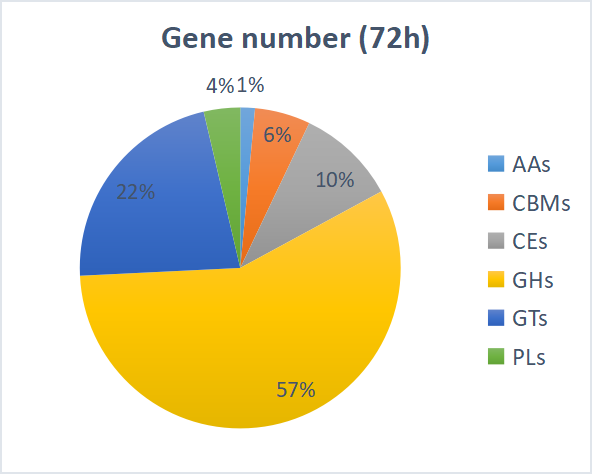

Supplement: Supplemental Information 3 [file peerj-13-20386-s003.zip › Raw data 3 Structural of microbial communities/CAZy.class.geneNum.72h.png]

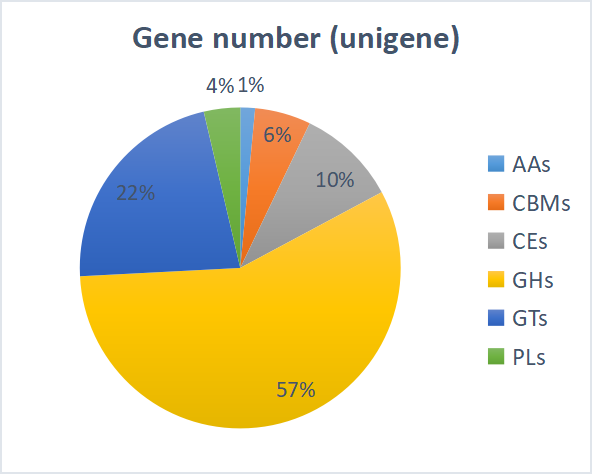

Supplement: Supplemental Information 3 [file peerj-13-20386-s003.zip › Raw data 3 Structural of microbial communities/CAZy.class.geneNum.total.png]

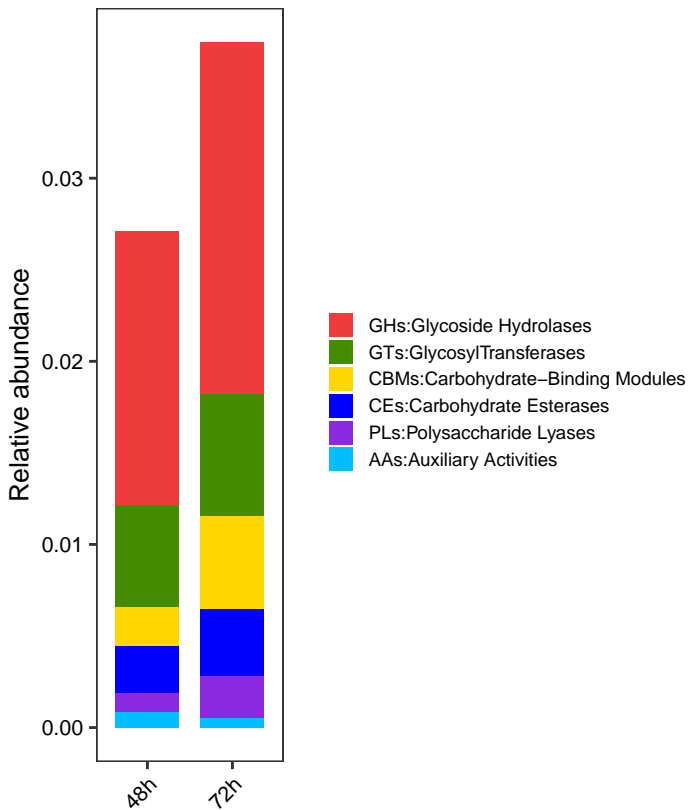

Supplement: Supplemental Information 3 [file peerj-13-20386-s003.zip › Raw data 3 Structural of microbial communities/CAZy.pdf]

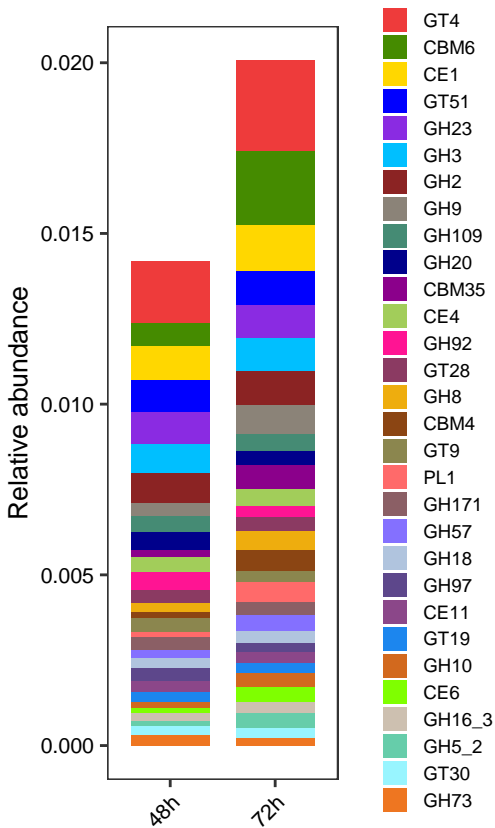

Supplement: Supplemental Information 3 [file peerj-13-20386-s003.zip › Raw data 3 Structural of microbial communities/CAZytop30.pdf]

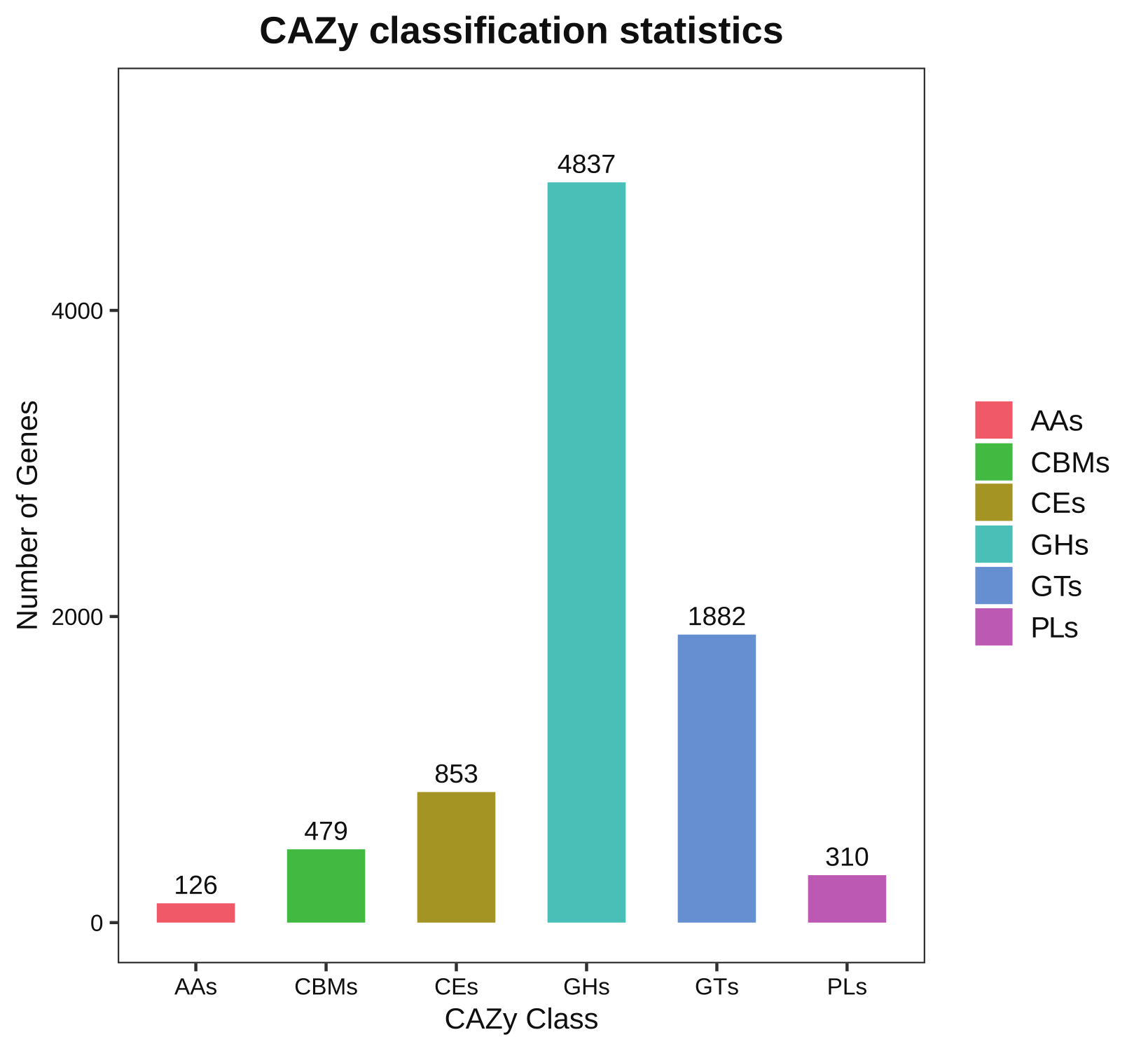

Supplement: Supplemental Information 3 [file peerj-13-20386-s003.zip › Raw data 3 Structural of microbial communities/CAZy_classification.png]

# eggNOG classification statistics

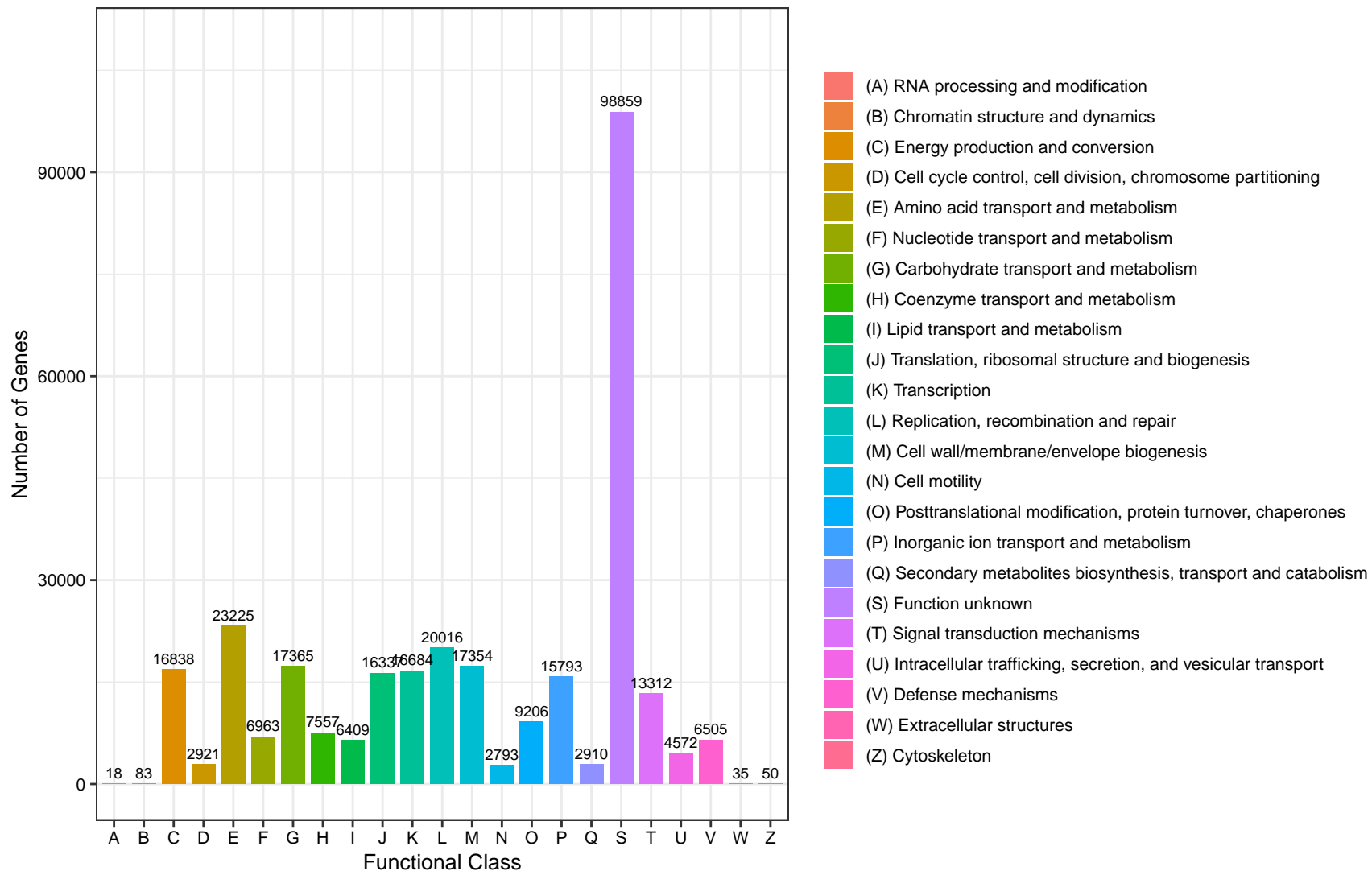

Supplement: Supplemental Information 3 [file peerj-13-20386-s003.zip › Raw data 3 Structural of microbial communities/eggNOG_classification.pdf]

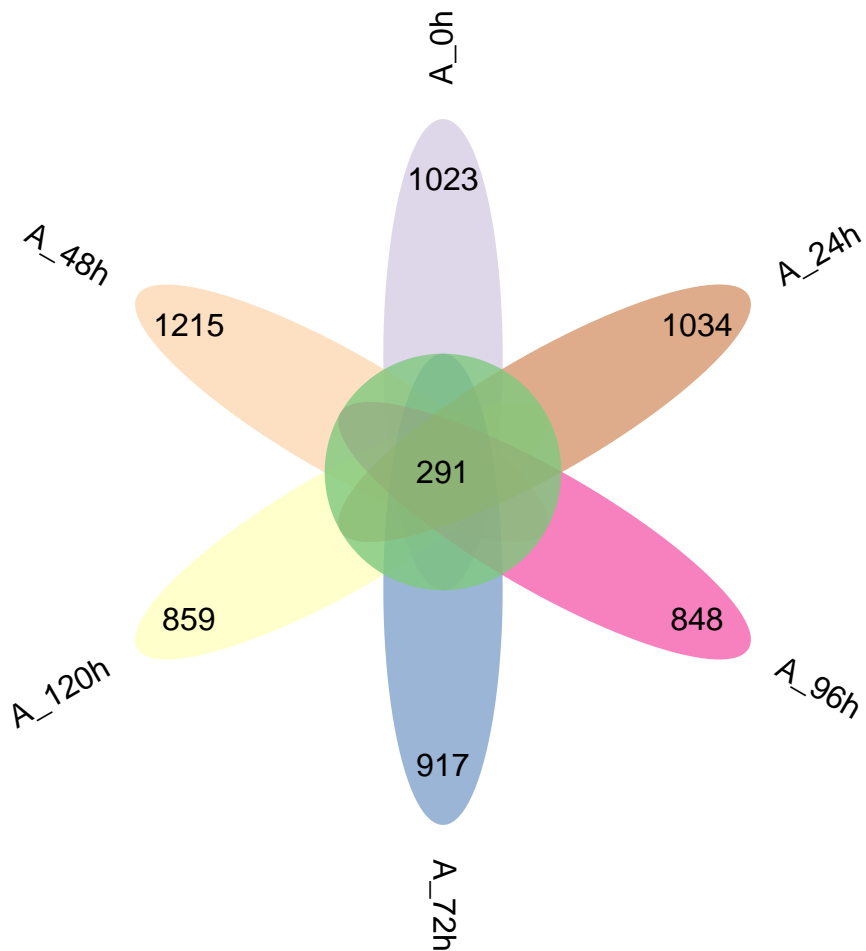

Supplement: Supplemental Information 3 [file peerj-13-20386-s003.zip › Raw data 3 Structural of microbial communities/flower.plot.pdf]

48h

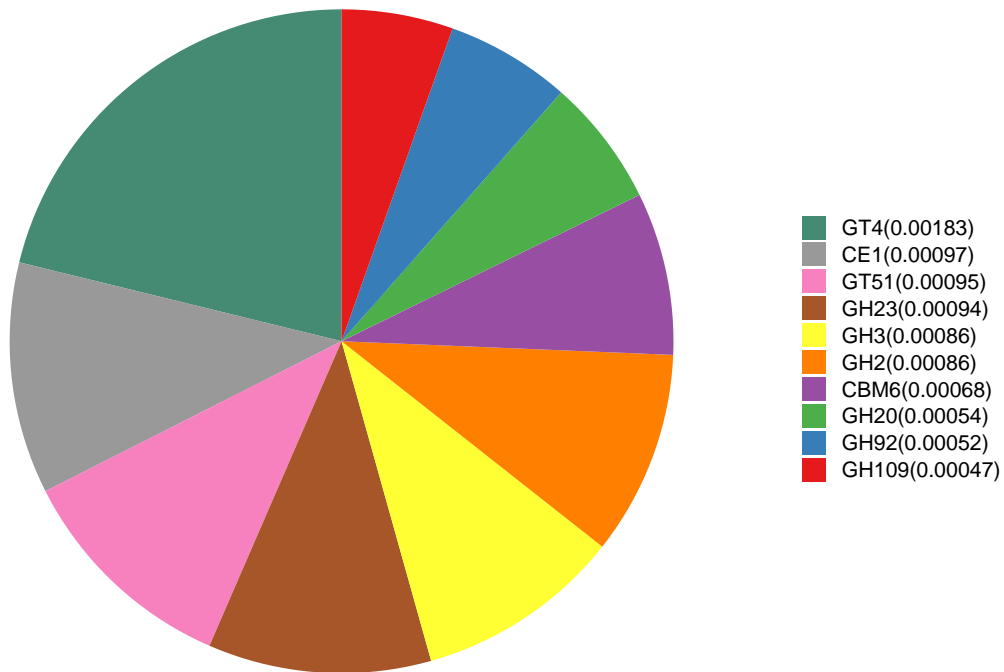

Supplement: Supplemental Information 3 [file peerj-13-20386-s003.zip › Raw data 3 Structural of microbial communities/Group.CAZy.family.percentage.top10.pie.48h.pdf]

72h

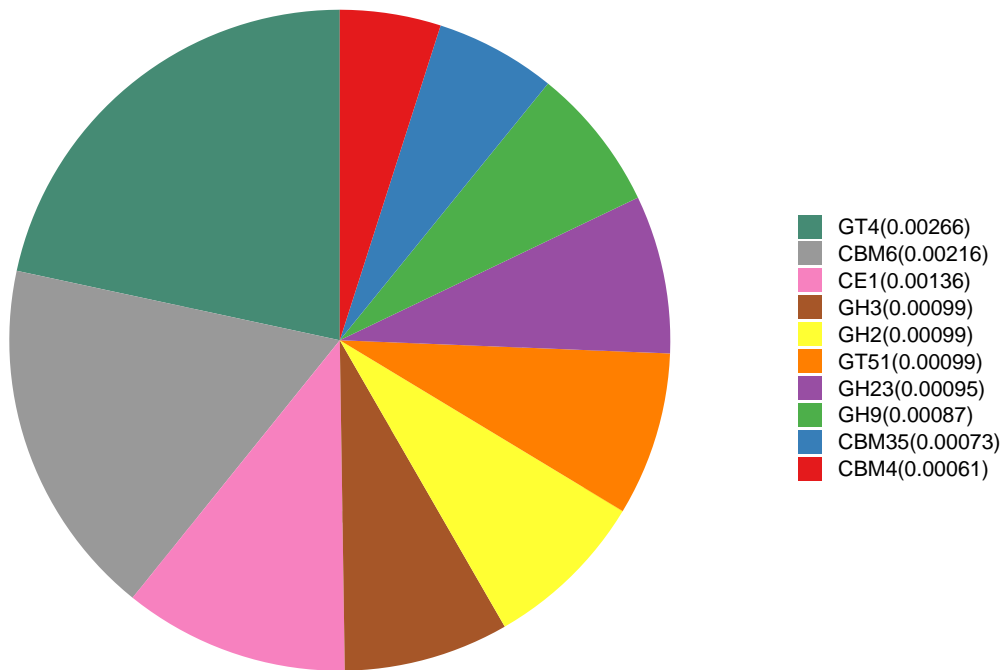

Supplement: Supplemental Information 3 [file peerj-13-20386-s003.zip › Raw data 3 Structural of microbial communities/Group.CAZy.family.percentage.top10.pie.72h.pdf]

48h

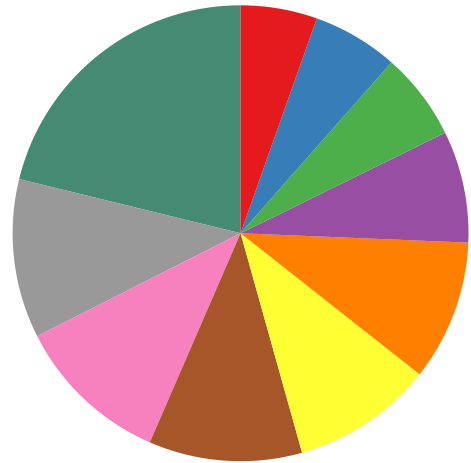

GT4(0.00183)  
CE1(0.00097)  
GT51(0.00095)  
GH23(0.00094)  
GH3(0.00086)  
GH2(0.00086)  
CBM6(0.00068)  
GH20(0.00054)  
GH92(0.00052)  
GH109(0.00047)

72h

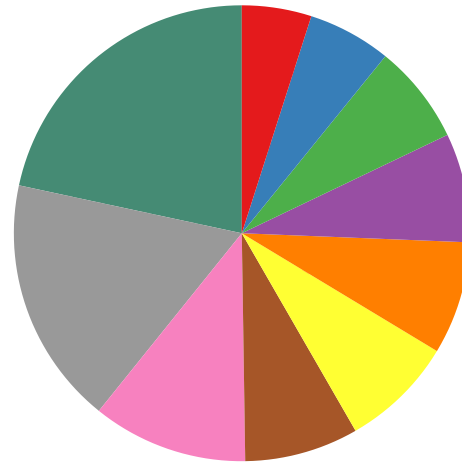

GT4(0.00266)  
CBM6(0.00216)  
CE1(0.00136)  
GH3(0.00099)  
GH2(0.00099)  
GT51(0.00099)  
GH23(0.00095)  
GH9(0.00087)  
CBM35(0.00073)  
CBM4(0.00061)

Supplement: Supplemental Information 3 [file peerj-13-20386-s003.zip › Raw data 3 Structural of microbial communities/Group.CAZy.family.percentage.top10.pie.all.pdf]

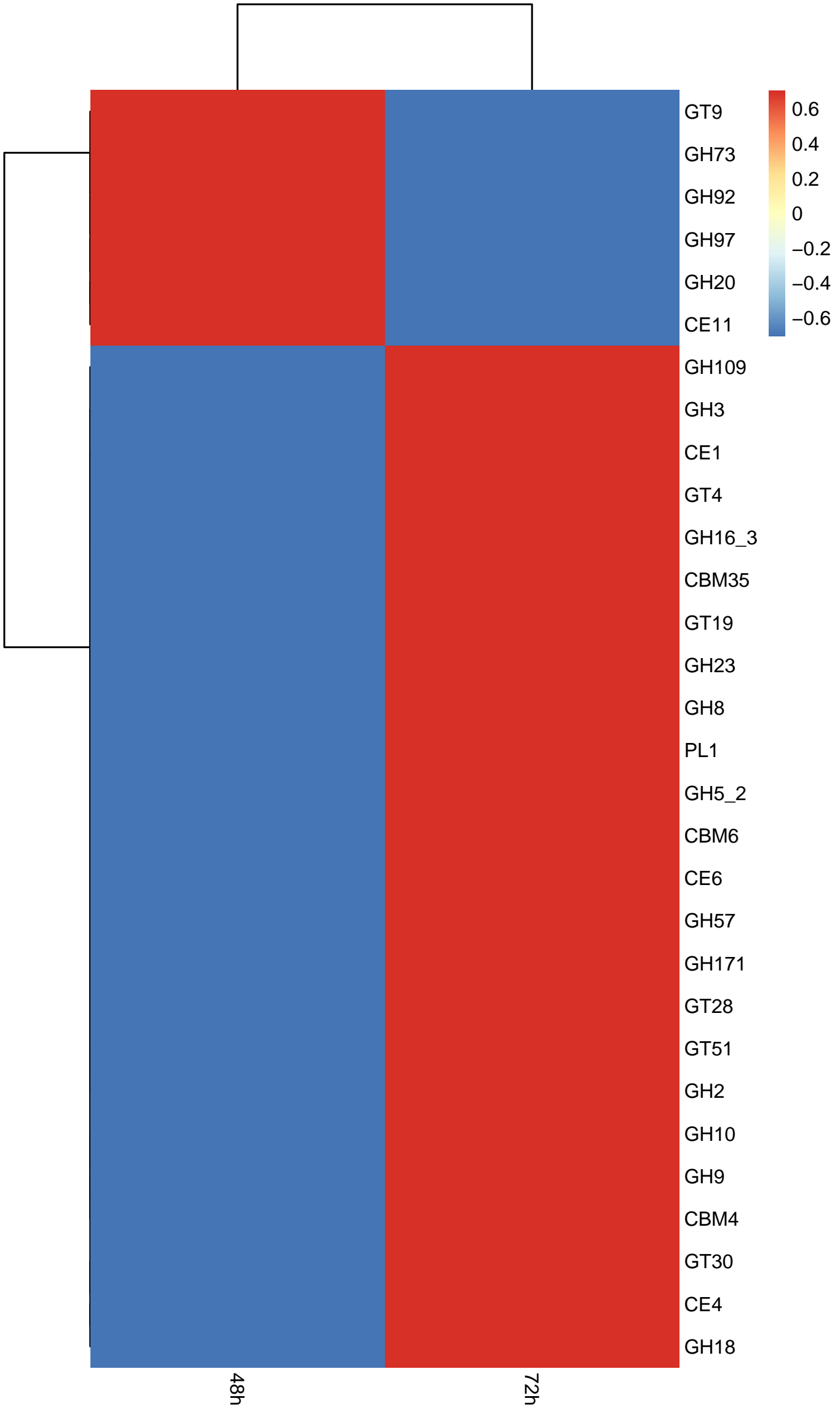

Supplement: Supplemental Information 3 [file peerj-13-20386-s003.zip › Raw data 3 Structural of microbial communities/Group.CAZy.family.TPM.top30.heatmap.pdf]

48h

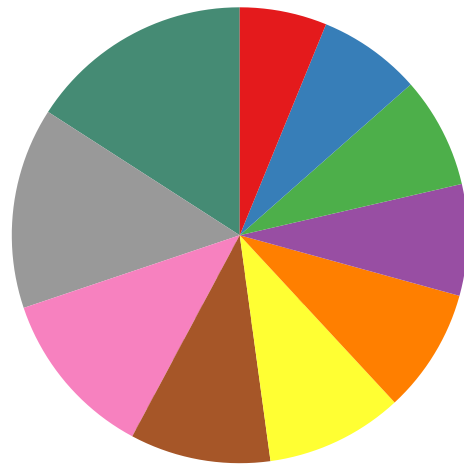

EC:3.6.4.12(0.00389)  
EC:2.7.7.7(0.00349)  
EC:2.7.13.3(0.00294)  
EC:5.2.1.8(0.00244)  
EC:3.1.--(0.00238)  
EC:7.1.1.2(0.00216)  
EC:3.4.16.4(0.00193)  
EC:3.1.21.3(0.00193)  
EC:3.4.24.- (0.00179)  
EC:2.7.7.6(0.00151)

72h

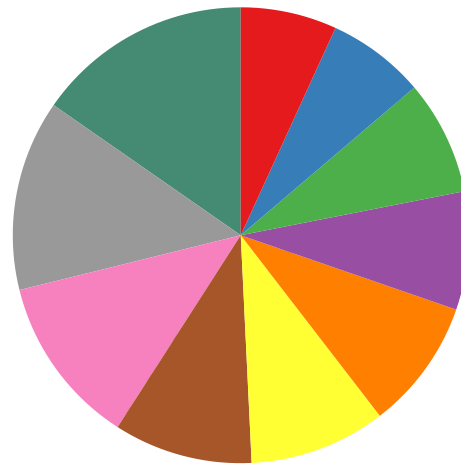

EC:3.6.4.12(0.00406)  
EC:2.7.7.7(0.00359)  
EC:7.1.1.2(0.0032)  
EC:3.1.--(0.00261)  
EC:5.2.1.8(0.00257)  
EC:2.7.13.3(0.00246)  
EC:3.2.1.4(0.00222)  
EC:3.1.21.3(0.00216)  
EC:3.4.16.4(0.00184)  
EC:3.4.24.- (0.00181)

Supplement: Supplemental Information 3 [file peerj-13-20386-s003.zip › Raw data 3 Structural of microbial communities/Group.KEGG.enzyme.percentage.top10.pie.all.pdf]

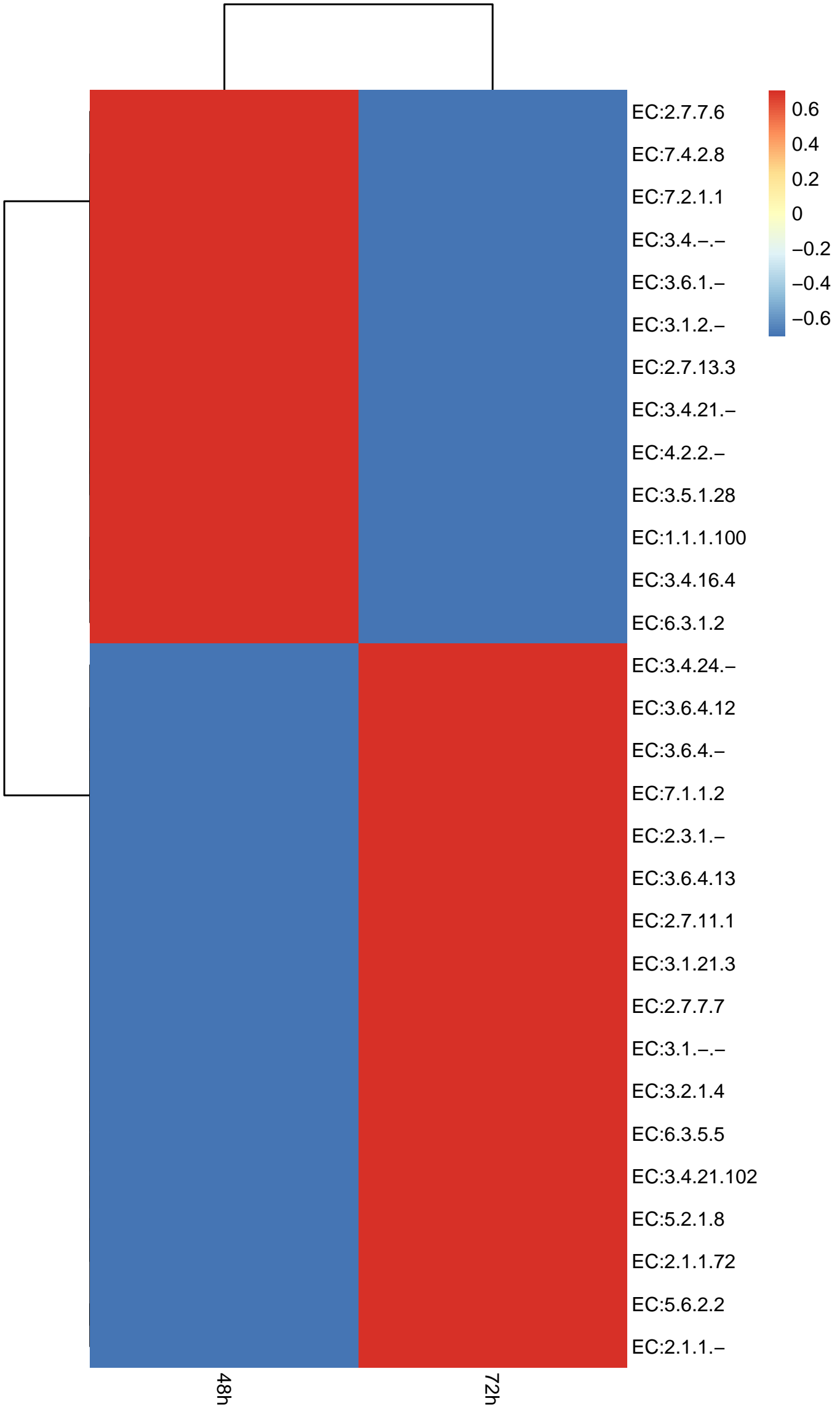

Supplement: Supplemental Information 3 [file peerj-13-20386-s003.zip › Raw data 3 Structural of microbial communities/Group.KEGG.enzyme.TPM.top30.heatmap.pdf]

48h

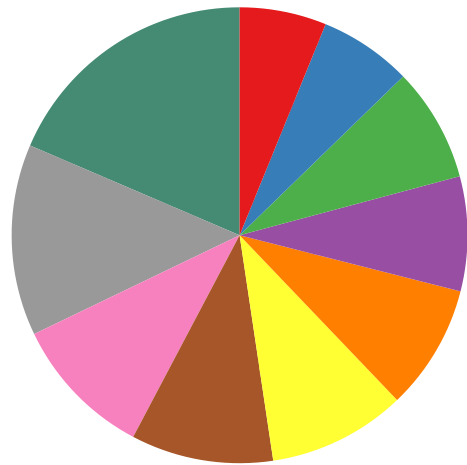

K21572(0.00316)  
K03406(0.00231)  
K03088(0.00173)  
K01992(0.00172)  
K07497(0.00167)  
K01990(0.00152)  
K07133(0.00138)  
K02004(0.00138)  
K02014(0.00112)  
K00059(0.00105)

72h

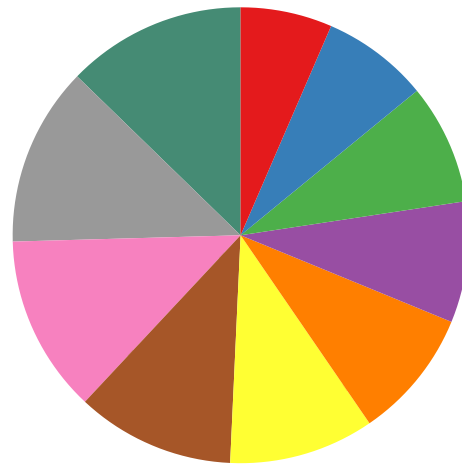

K21572(0.00226)  
K07497(0.00226)  
K01179(0.00224)  
K03406(0.002)  
K01992(0.00183)  
K01990(0.00164)  
K07133(0.00153)  
K03088(0.00152)  
K02004(0.00135)  
K03427(0.00116)

Supplement: Supplemental Information 3 [file peerj-13-20386-s003.zip › Raw data 3 Structural of microbial communities/Group.KEGG.KO.percentage.top10.pie.all.pdf]

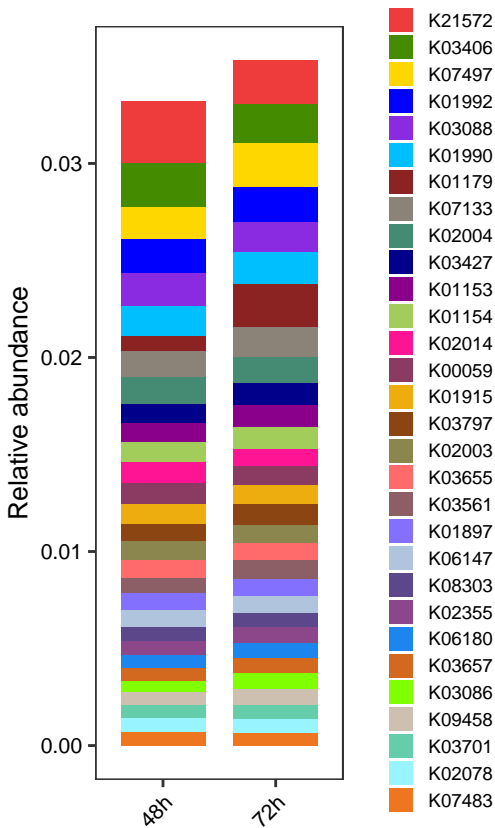

Supplement: Supplemental Information 3 [file peerj-13-20386-s003.zip › Raw data 3 Structural of microbial communities/Group.KEGG.KO.percentage.top30.histogram.pdf]

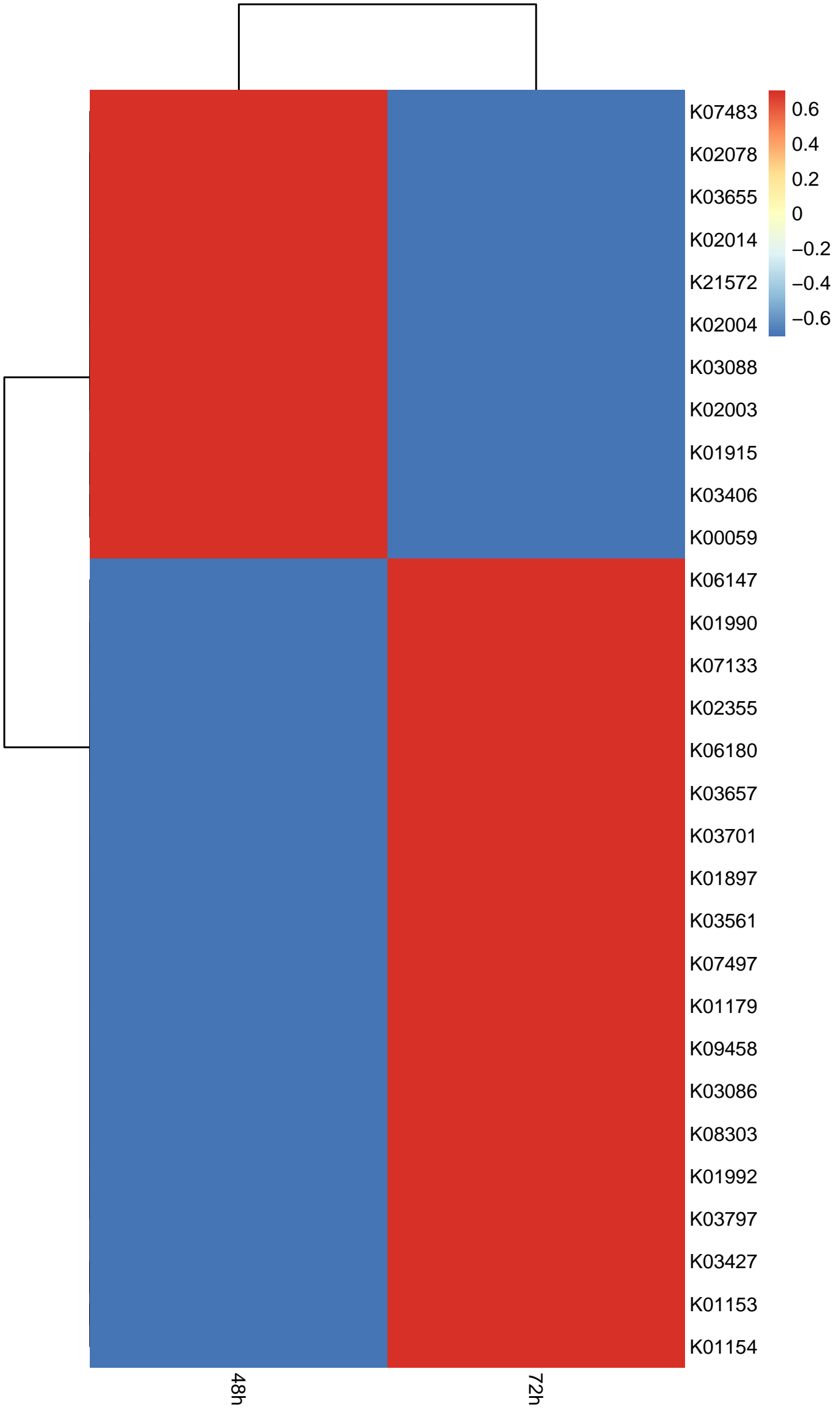

Supplement: Supplemental Information 3 [file peerj-13-20386-s003.zip › Raw data 3 Structural of microbial communities/Group.KEGG.KO.TPM.top30.heatmap.pdf]

48h

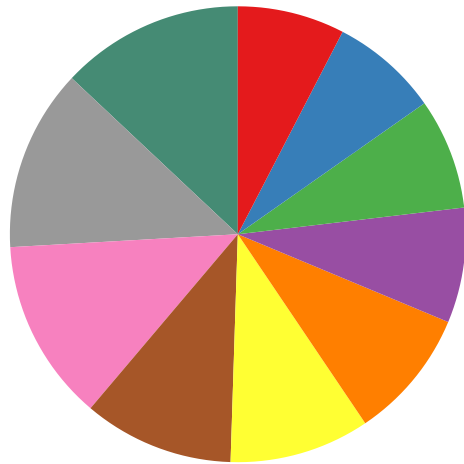

M00009(0.00485)  
M00614(0.00482)  
M00173(0.00482)  
M00011(0.00398)  
M00001(0.00371)  
M00048(0.00346)  
M00003(0.00305)  
M00083(0.00294)  
M00051(0.00286)  
M00016(0.00284)

72h

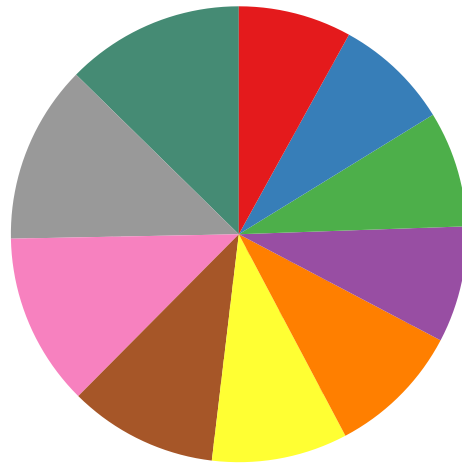

M00173(0.0045)  
M00614(0.0045)  
M00009(0.00435)  
M00001(0.00375)  
M00048(0.00342)  
M00011(0.00339)  
M00083(0.00293)  
M00003(0.00292)  
M00051(0.00291)  
M00026(0.00285)

Supplement: Supplemental Information 3 [file peerj-13-20386-s003.zip › Raw data 3 Structural of microbial communities/Group.KEGG.module.percentage.top10.pie.all.pdf]

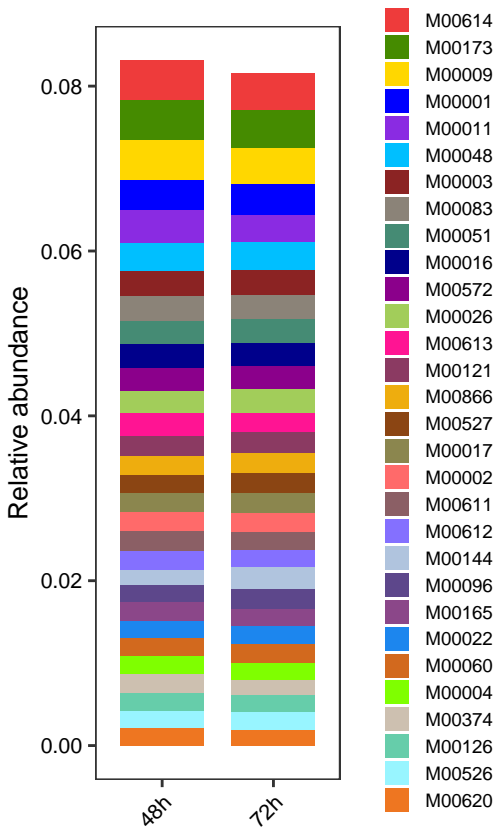

Supplement: Supplemental Information 3 [file peerj-13-20386-s003.zip › Raw data 3 Structural of microbial communities/Group.KEGG.module.percentage.top30.histogram.pdf]

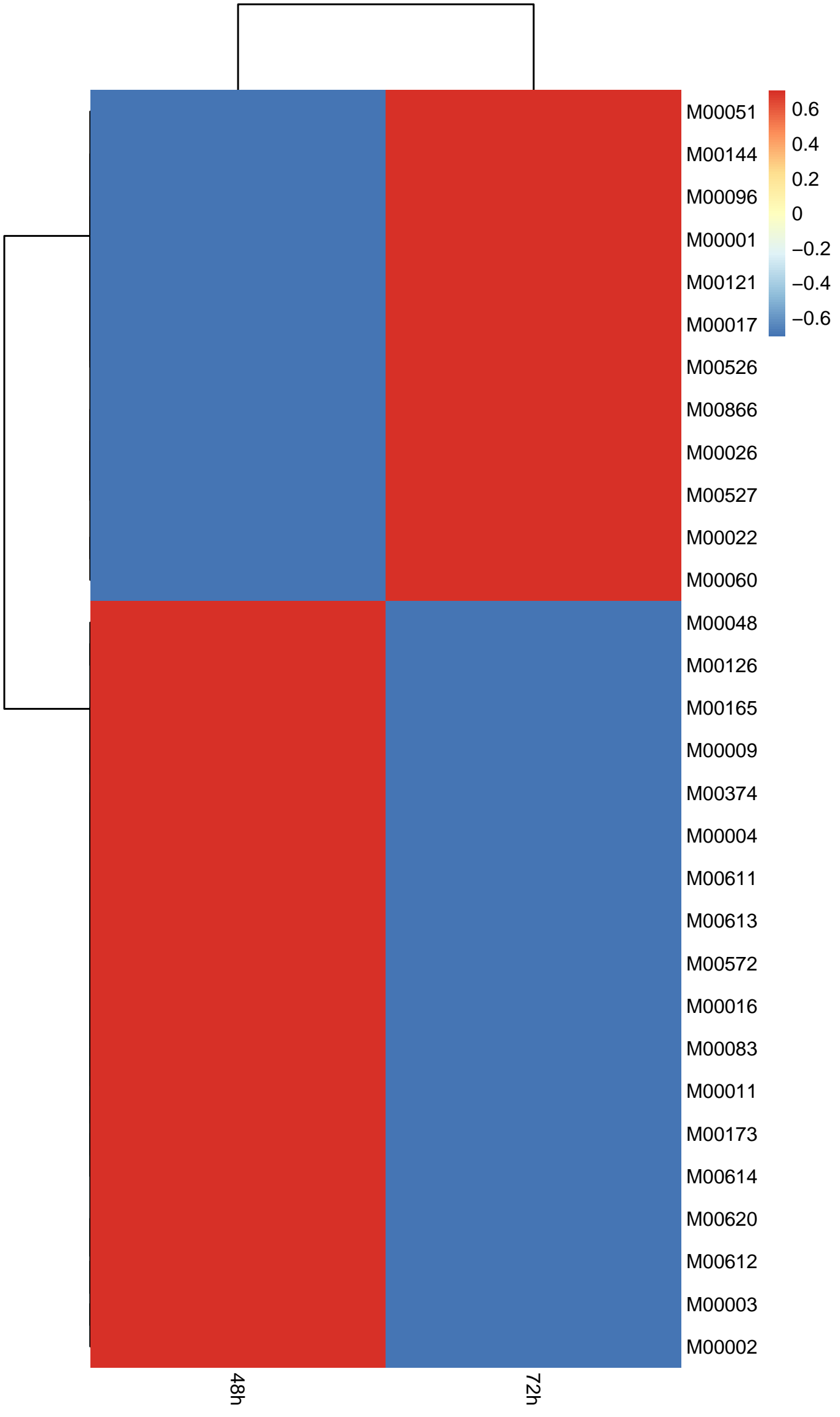

Supplement: Supplemental Information 3 [file peerj-13-20386-s003.zip › Raw data 3 Structural of microbial communities/Group.KEGG.module.TPM.top30.heatmap.pdf]

48h

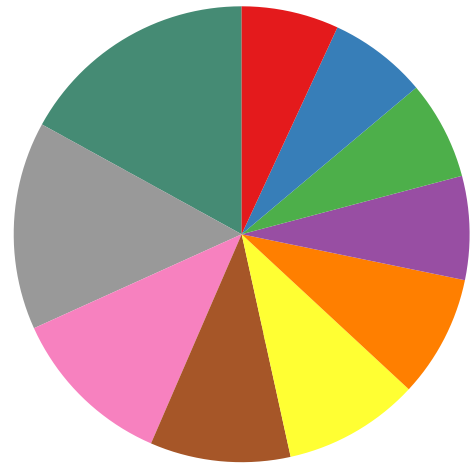

ko02020(0.01844)  
ko02010(0.01601)  
ko03010(0.01273)  
ko00230(0.0108)  
ko02024(0.01047)  
ko00620(0.00941)  
ko00190(0.00798)  
ko00520(0.00759)  
ko00270(0.00757)  
ko00240(0.00749)

72h

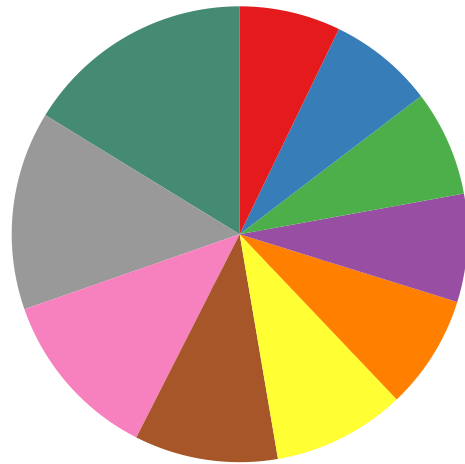

ko02020(0.01711)  
ko02010(0.01483)  
ko03010(0.01282)  
ko00230(0.01072)  
ko02024(0.00991)  
ko00620(0.00853)  
ko00520(0.00807)  
ko00190(0.0079)  
ko00970(0.00787)  
ko00240(0.00755)

Supplement: Supplemental Information 3 [file peerj-13-20386-s003.zip › Raw data 3 Structural of microbial communities/Group.KEGG.pathway.percentage.top10.pie.all.pdf]

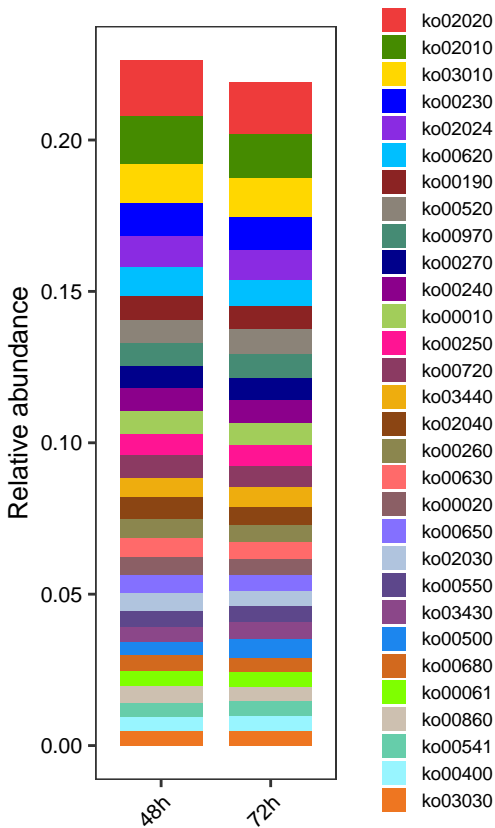

Supplement: Supplemental Information 3 [file peerj-13-20386-s003.zip › Raw data 3 Structural of microbial communities/Group.KEGG.pathway.percentage.top30.histogram.pdf]

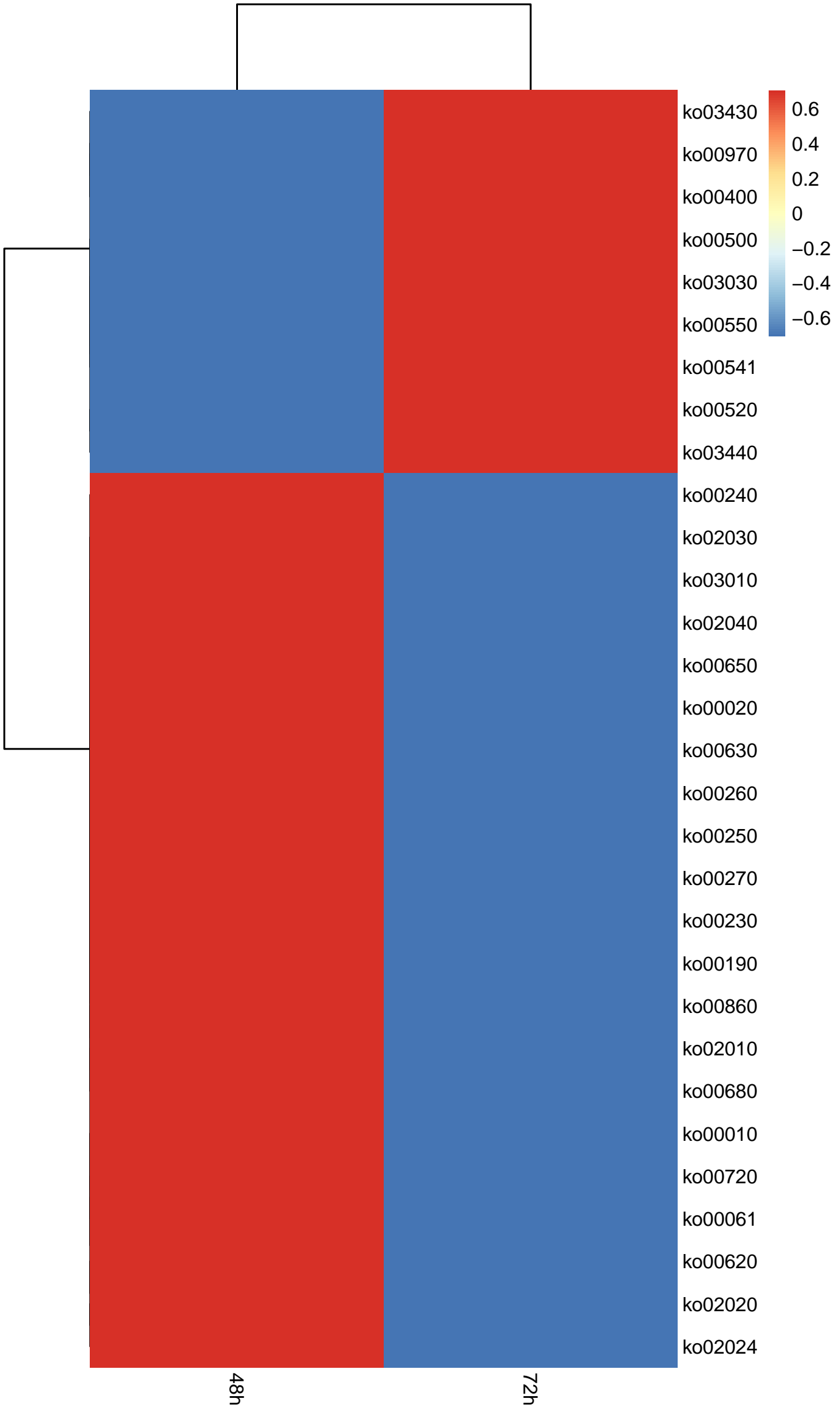

Supplement: Supplemental Information 3 [file peerj-13-20386-s003.zip › Raw data 3 Structural of microbial communities/Group.KEGG.pathway.TPM.top30.heatmap.pdf]

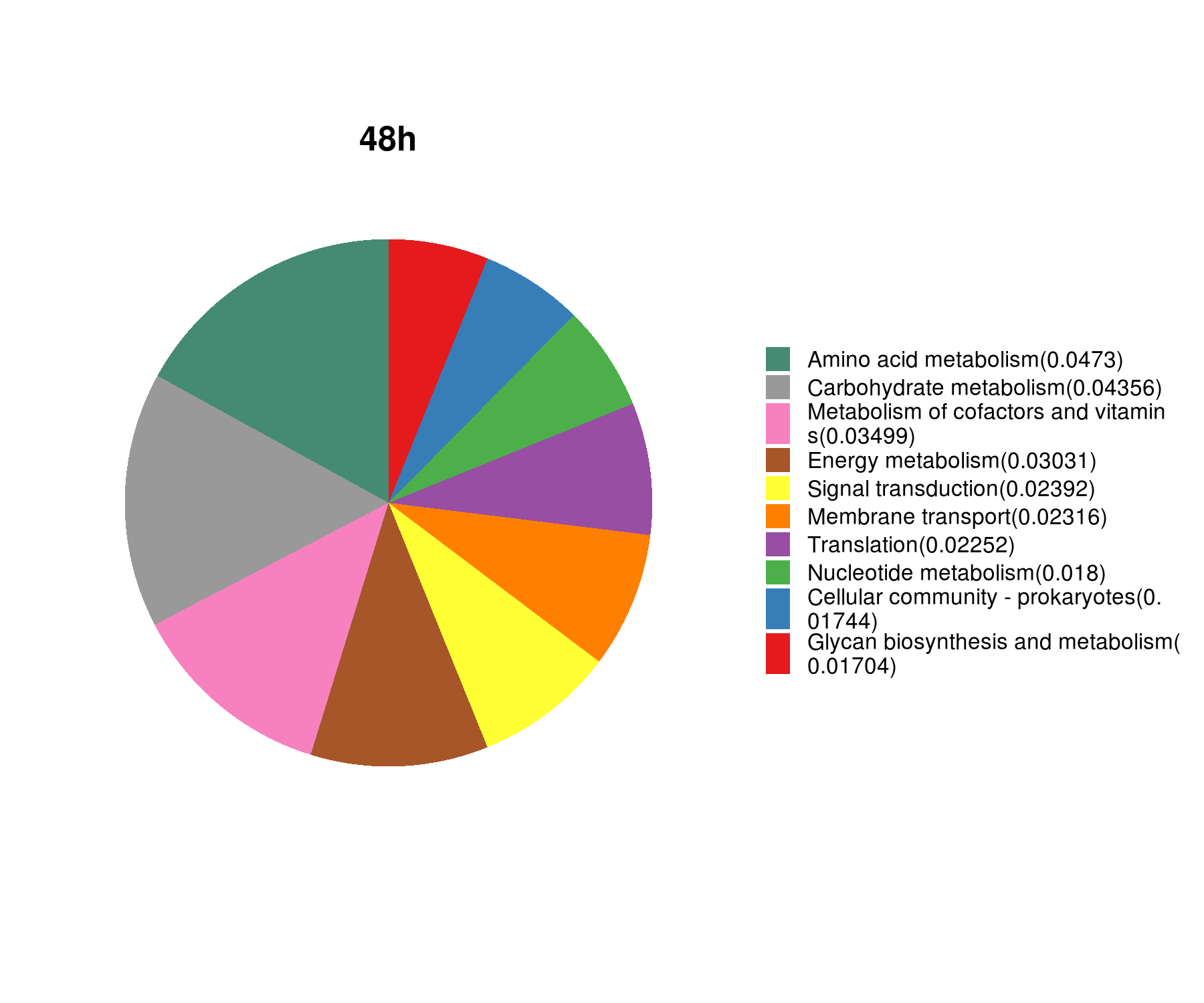

Supplement: Supplemental Information 3 [file peerj-13-20386-s003.zip › Raw data 3 Structural of microbial communities/Group.KEGG.pathway_hierarchy2.percentage.top10.pie.48h.png]

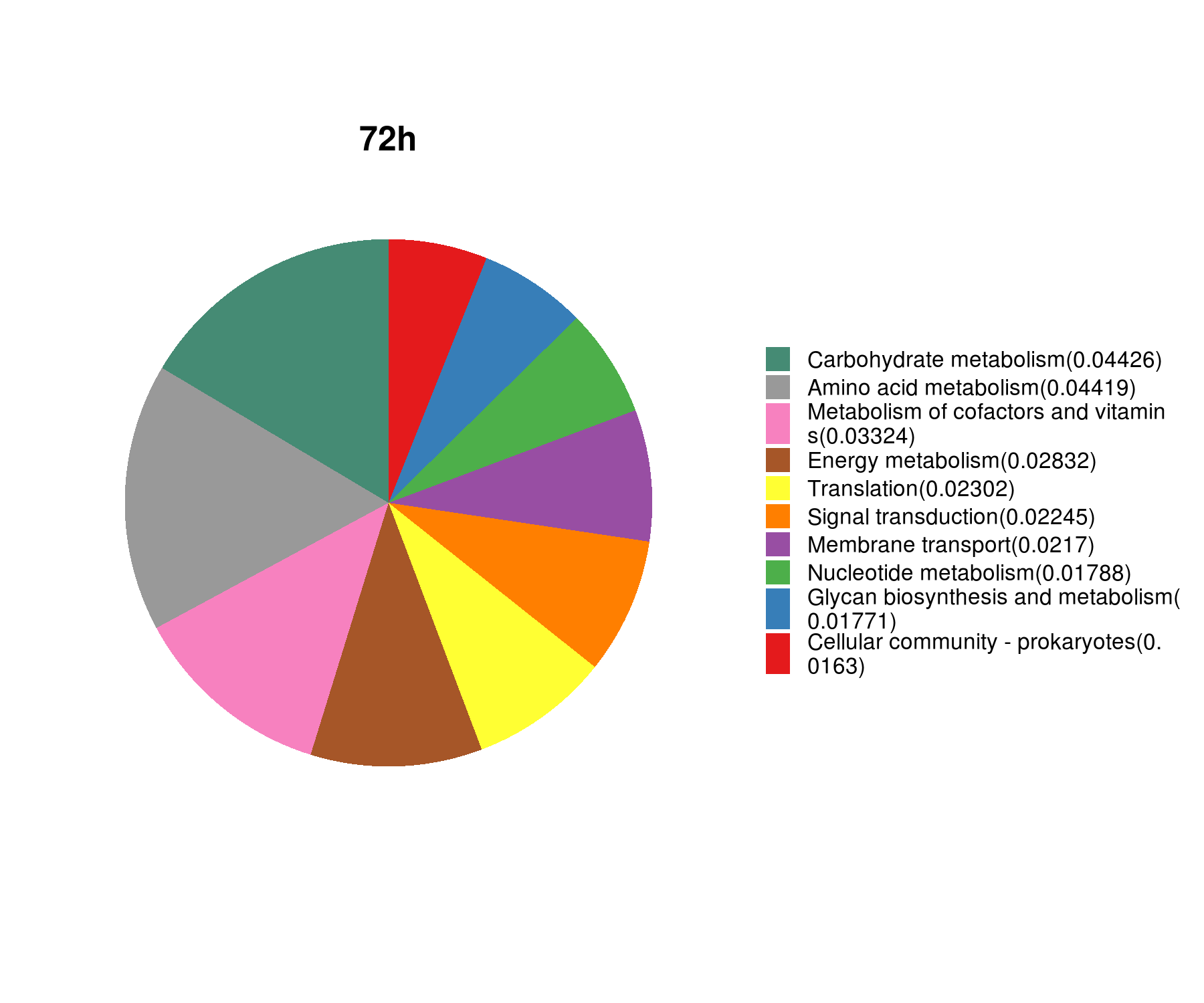

Supplement: Supplemental Information 3 [file peerj-13-20386-s003.zip › Raw data 3 Structural of microbial communities/Group.KEGG.pathway_hierarchy2.percentage.top10.pie.72h.png]

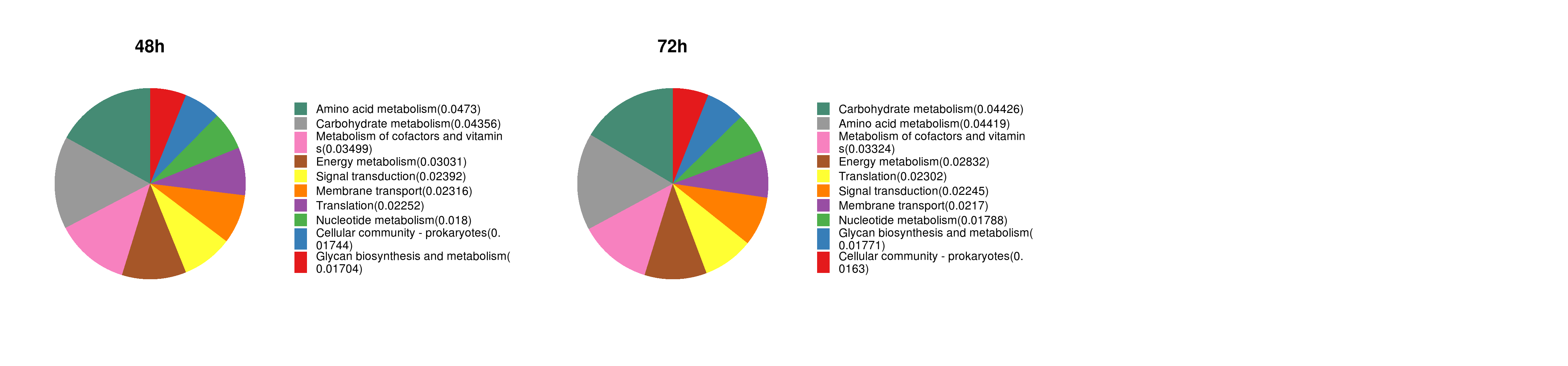

Supplement: Supplemental Information 3 [file peerj-13-20386-s003.zip › Raw data 3 Structural of microbial communities/Group.KEGG.pathway_hierarchy2.percentage.top10.pie.all.png]

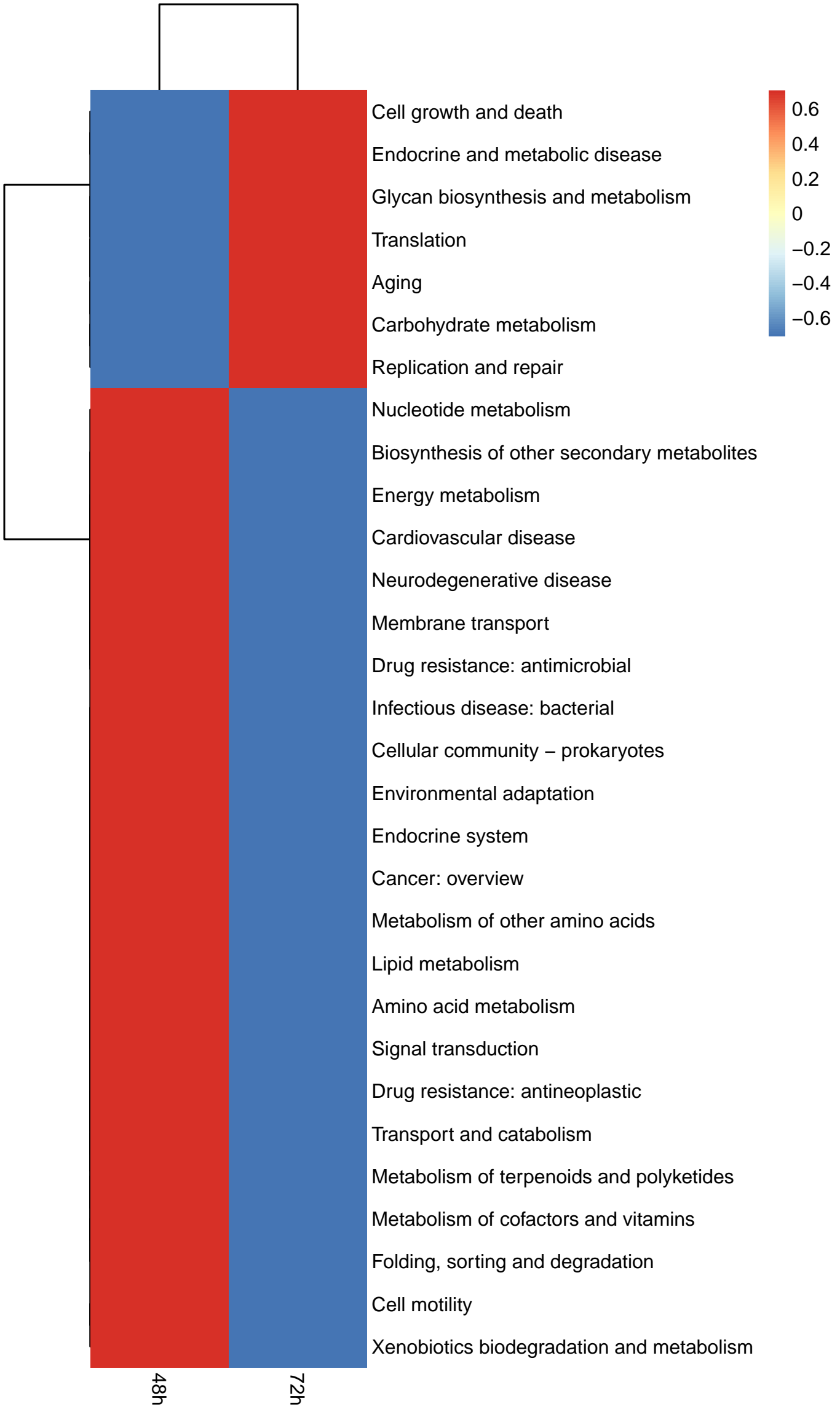

Supplement: Supplemental Information 3 [file peerj-13-20386-s003.zip › Raw data 3 Structural of microbial communities/Group.KEGG.pathway_hierarchy2.TPM.top30.heatmap.pdf]

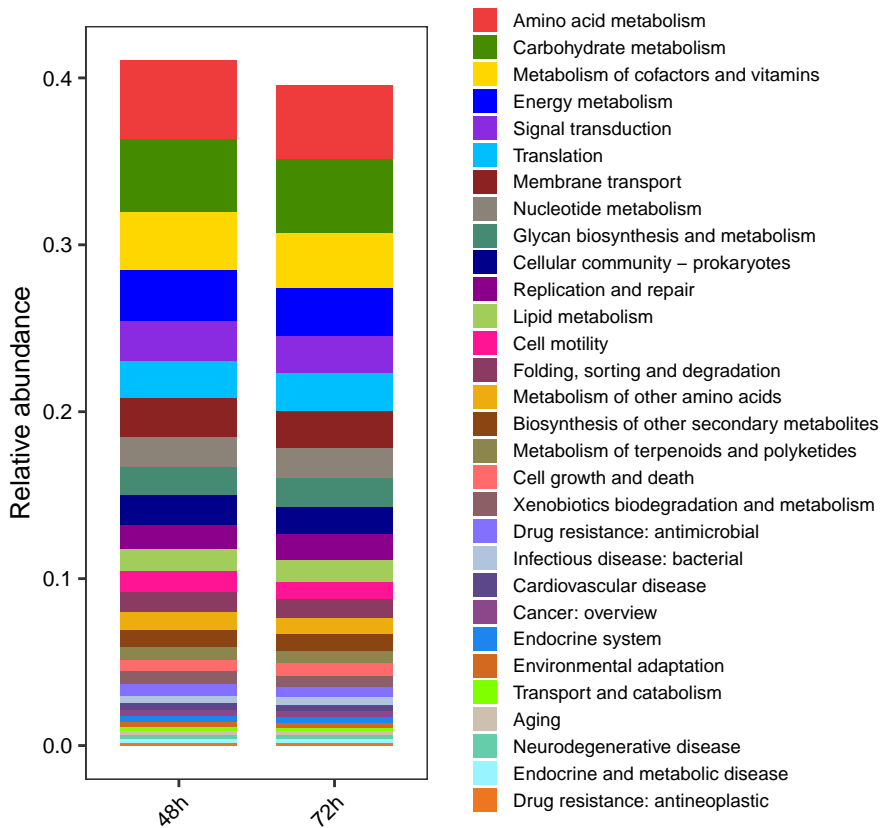

Supplement: Supplemental Information 3 [file peerj-13-20386-s003.zip › Raw data 3 Structural of microbial communities/KEGG.pathway.top30.pdf]

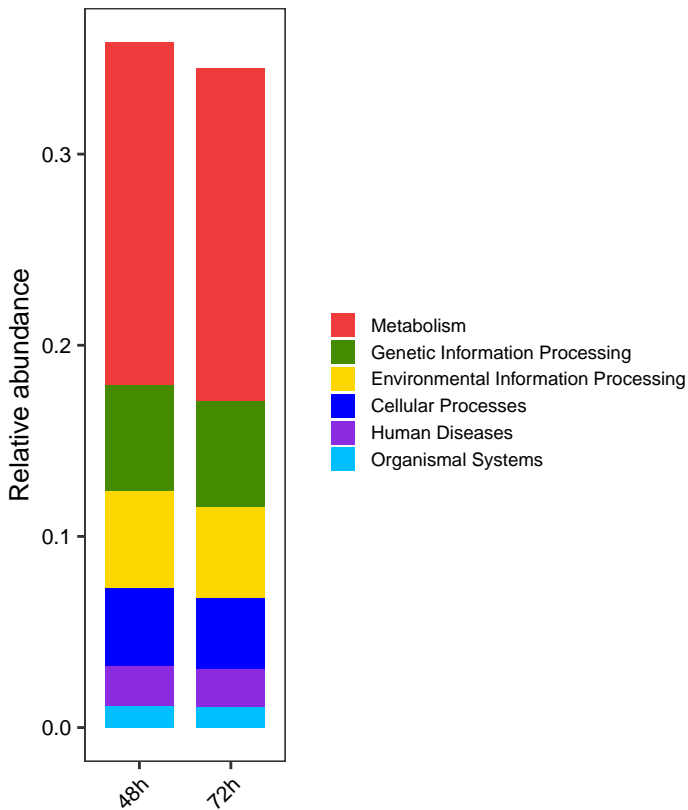

Supplement: Supplemental Information 3 [file peerj-13-20386-s003.zip › Raw data 3 Structural of microbial communities/KEGG.pathwayall.pdf]

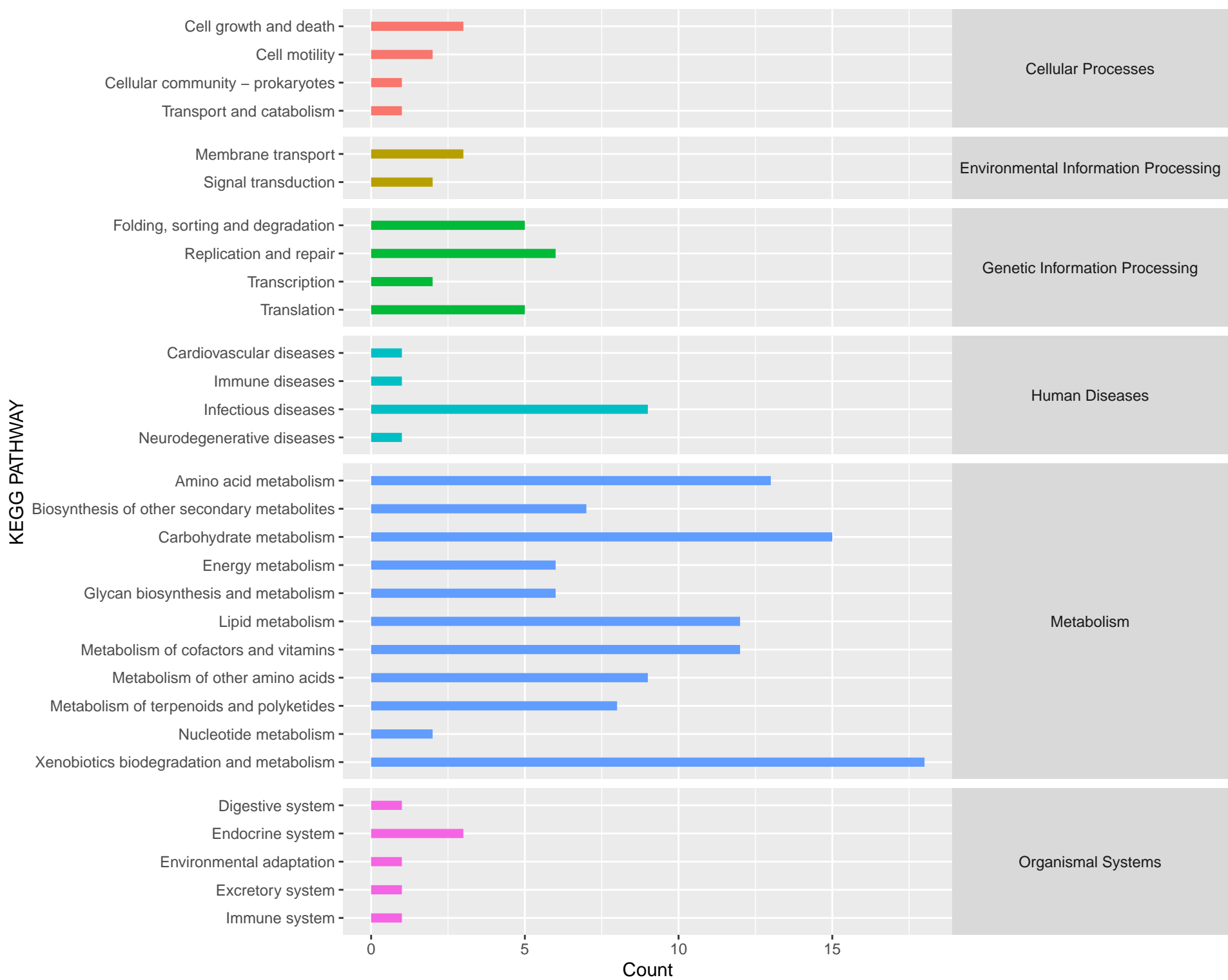

Supplement: Supplemental Information 3 [file peerj-13-20386-s003.zip › Raw data 3 Structural of microbial communities/KEGG_occur.pdf]

# KEGG classification statistics

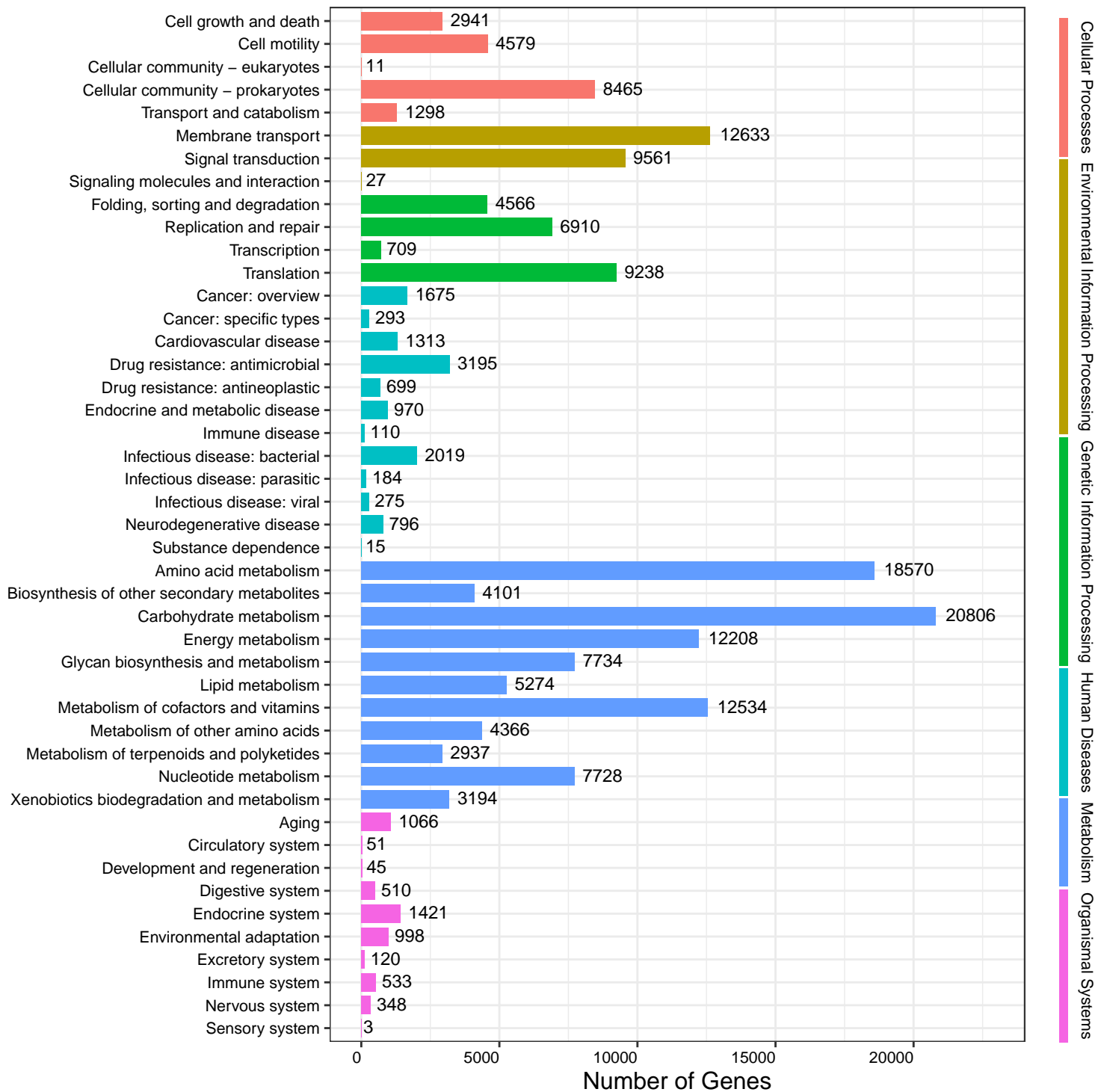

Supplement: Supplemental Information 3 [file peerj-13-20386-s003.zip › Raw data 3 Structural of microbial communities/KEGG_pathway_hierarchy2.pdf]

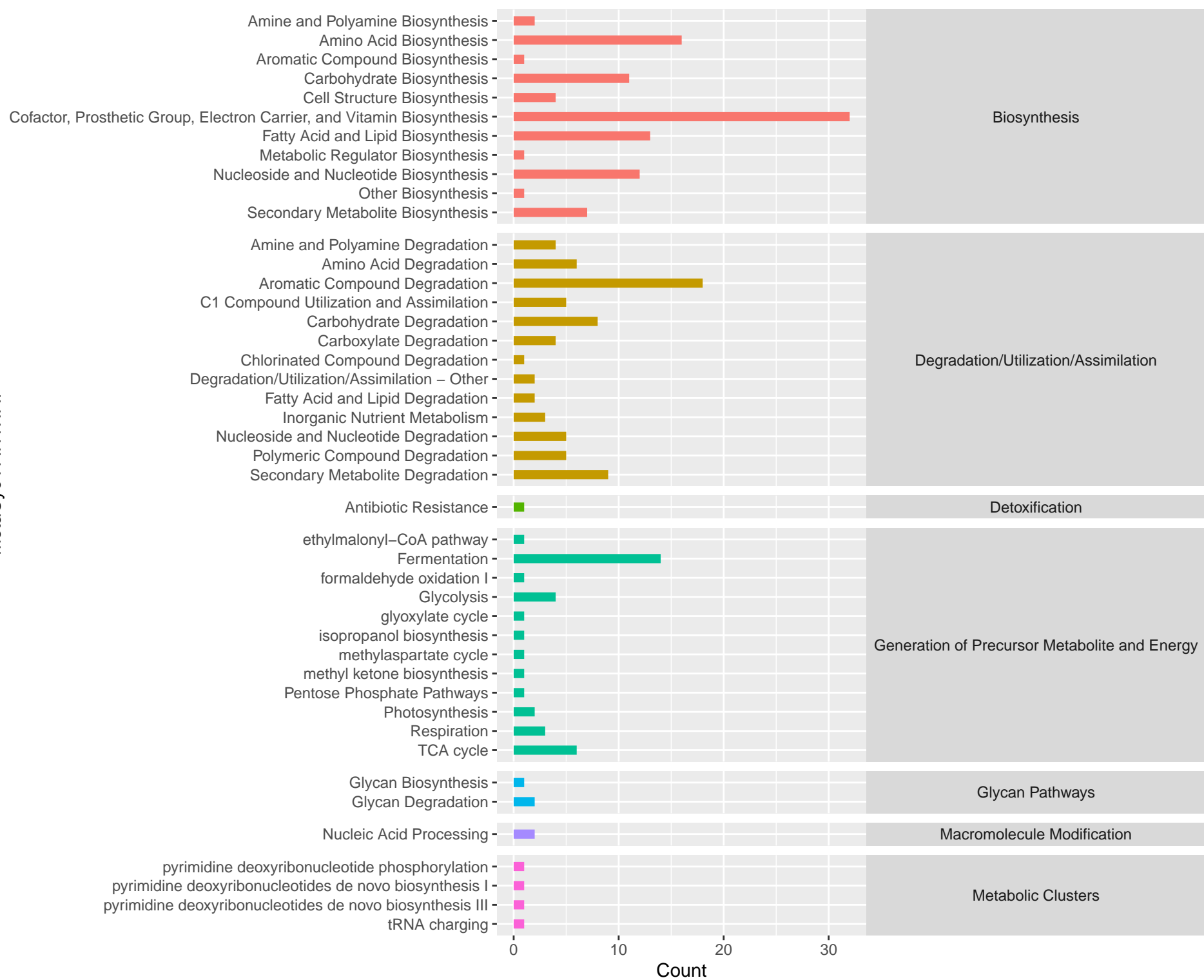

Supplement: Supplemental Information 3 [file peerj-13-20386-s003.zip › Raw data 3 Structural of microbial communities/MetaCyc_occur.pdf]

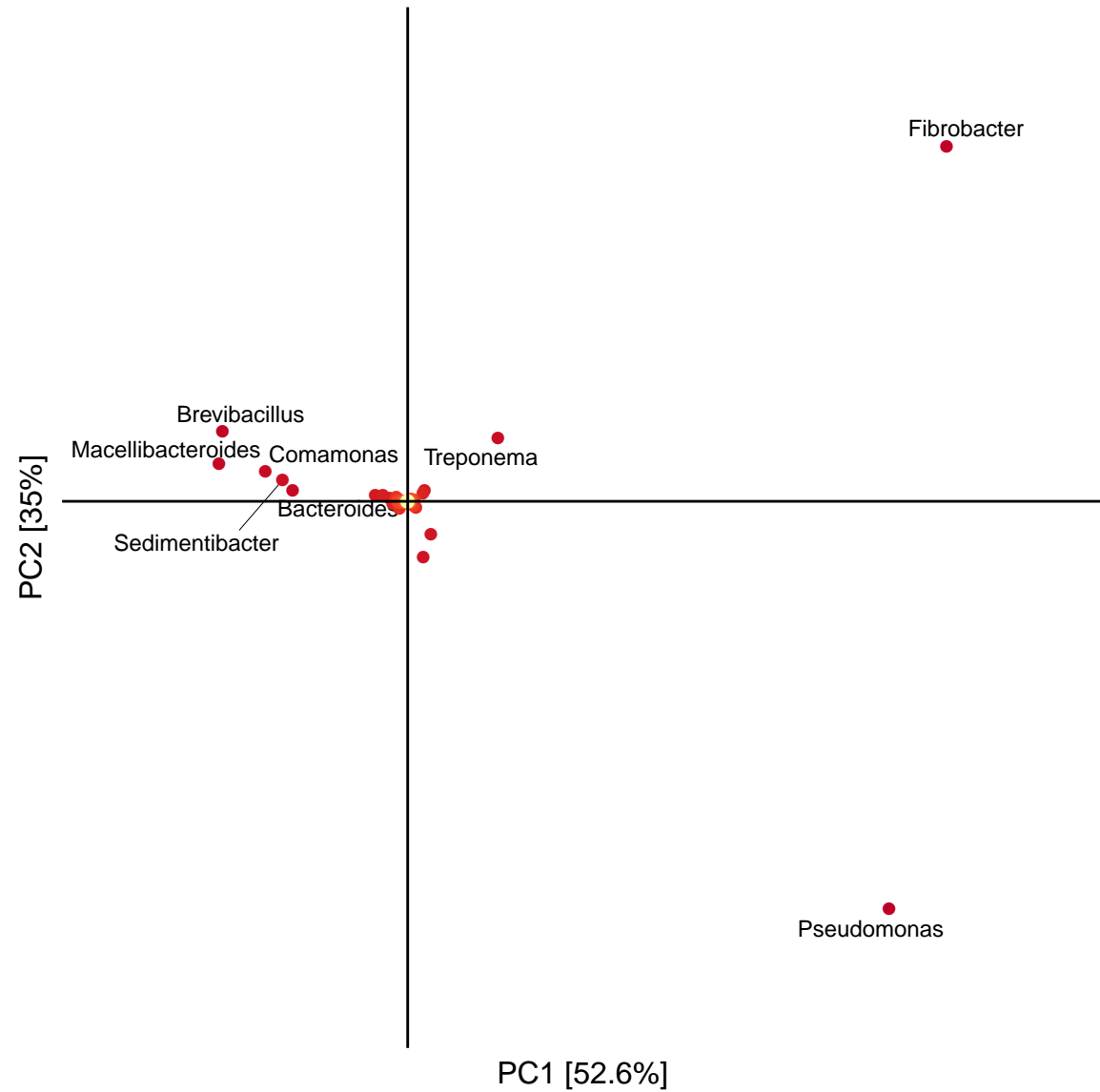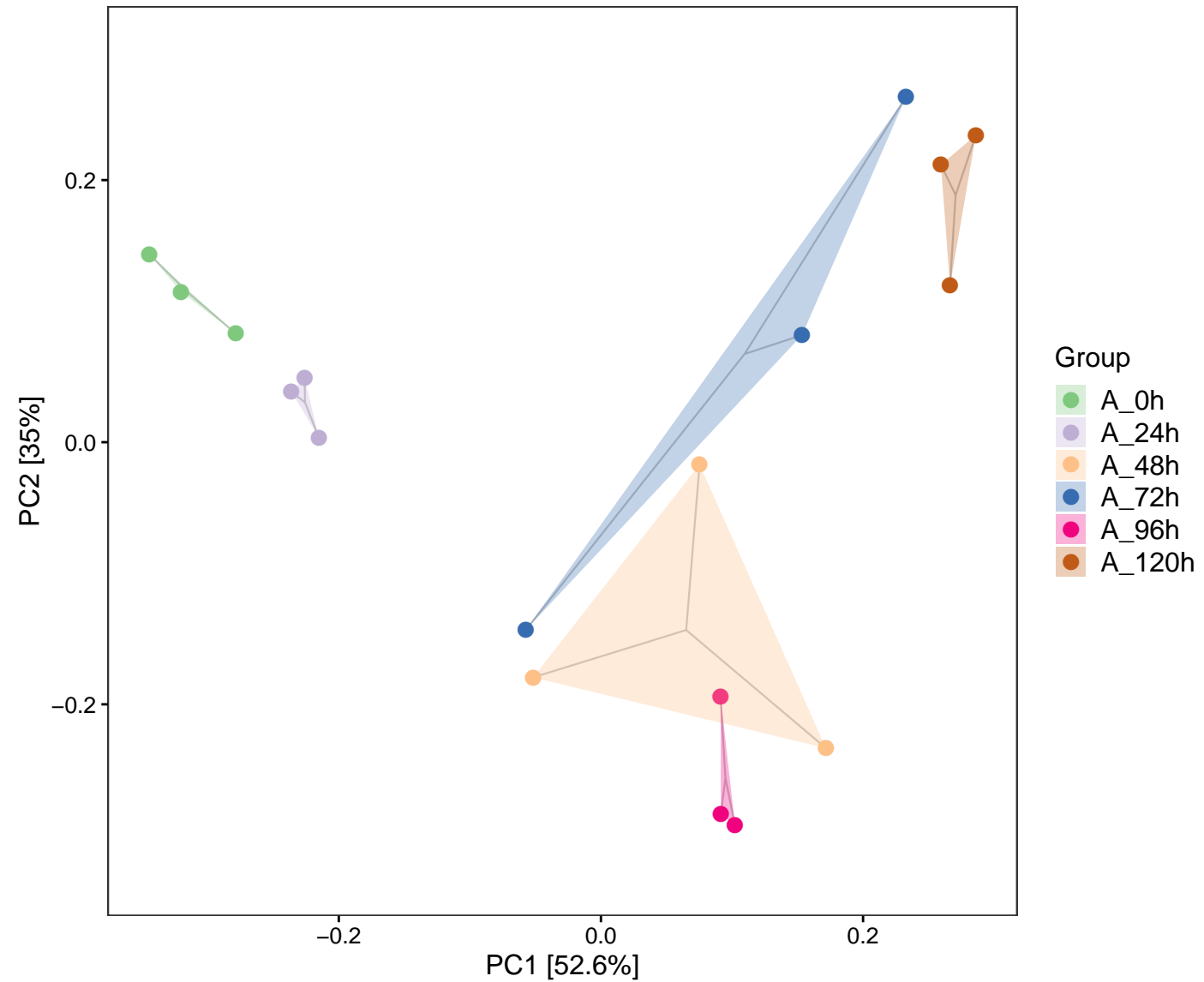

Supplement: Supplemental Information 3 [file peerj-13-20386-s003.zip › Raw data 3 Structural of microbial communities/PCA.hull.pdf]

Rank Abundance Curve

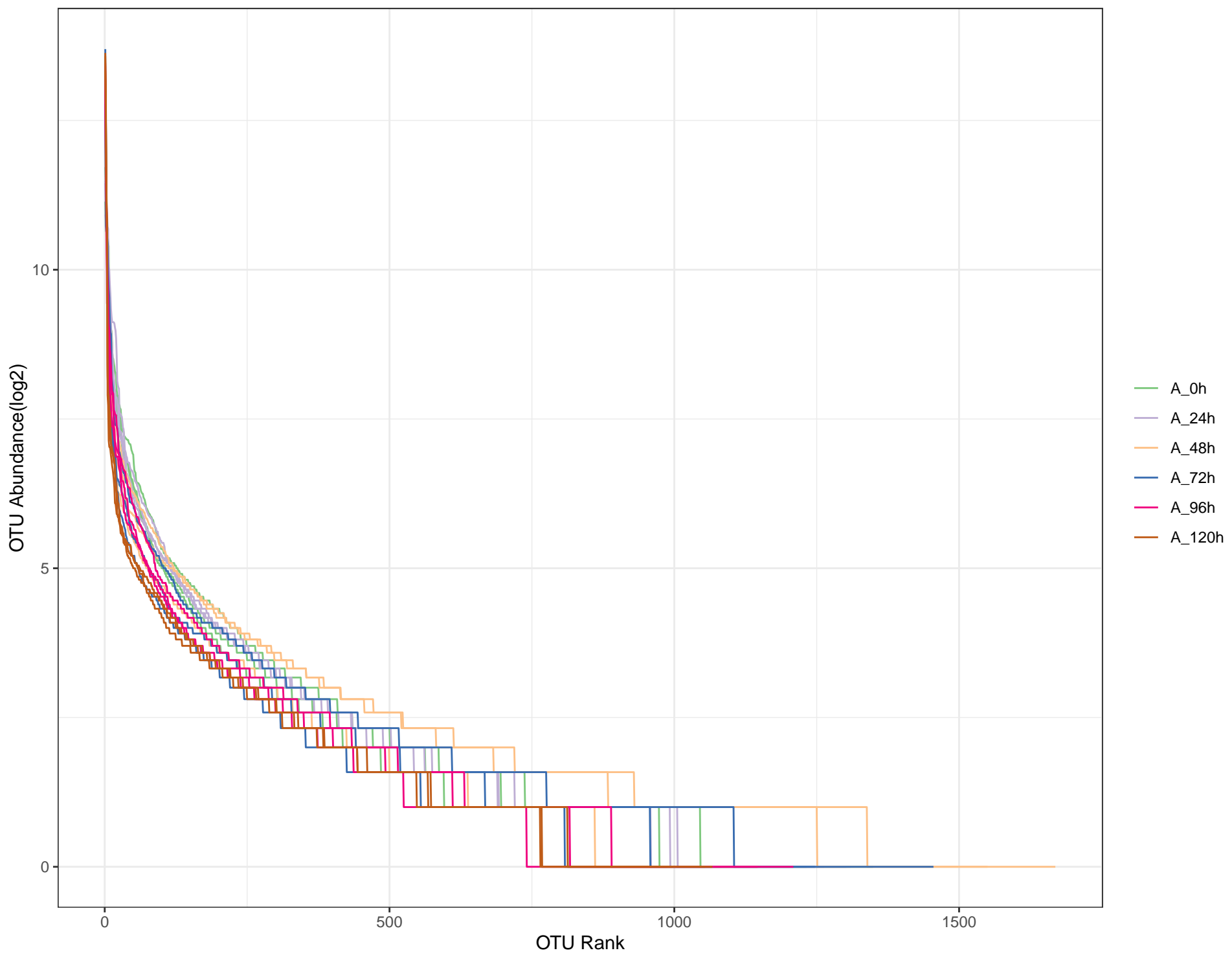

Supplement: Supplemental Information 3 [file peerj-13-20386-s003.zip › Raw data 3 Structural of microbial communities/rabund.pdf]

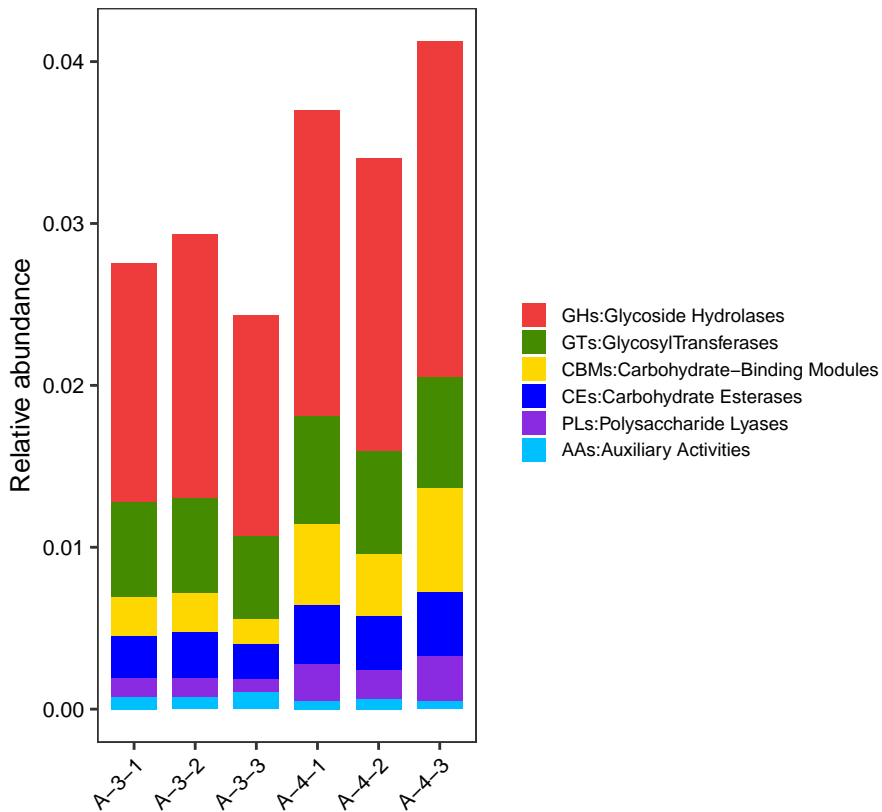

Supplement: Supplemental Information 3 [file peerj-13-20386-s003.zip › Raw data 3 Structural of microbial communities/Sample.CAZy.class.percentage.all.histogram.pdf]

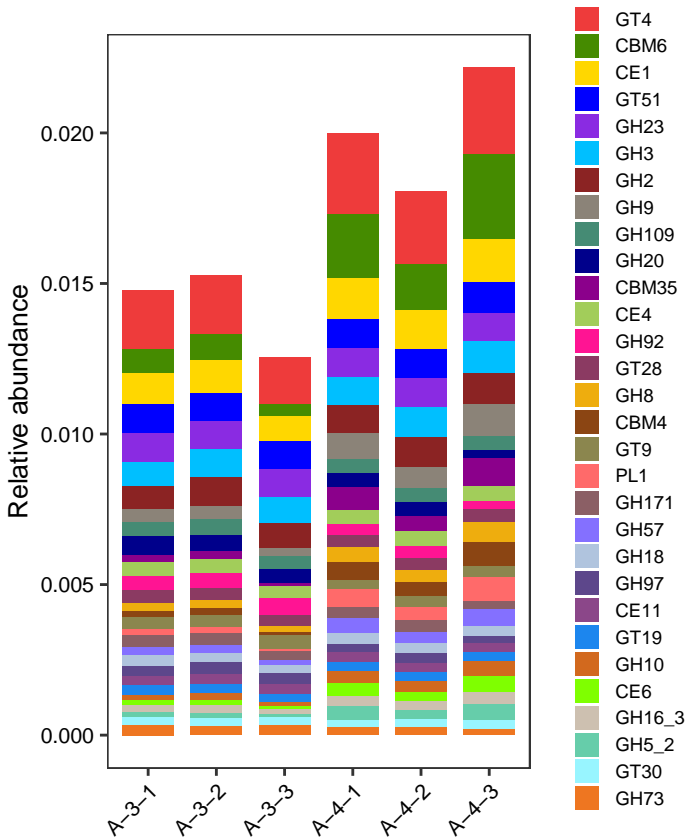

Supplement: Supplemental Information 3 [file peerj-13-20386-s003.zip › Raw data 3 Structural of microbial communities/Sample.CAZy.family.percentage.top30.histogram.pdf]

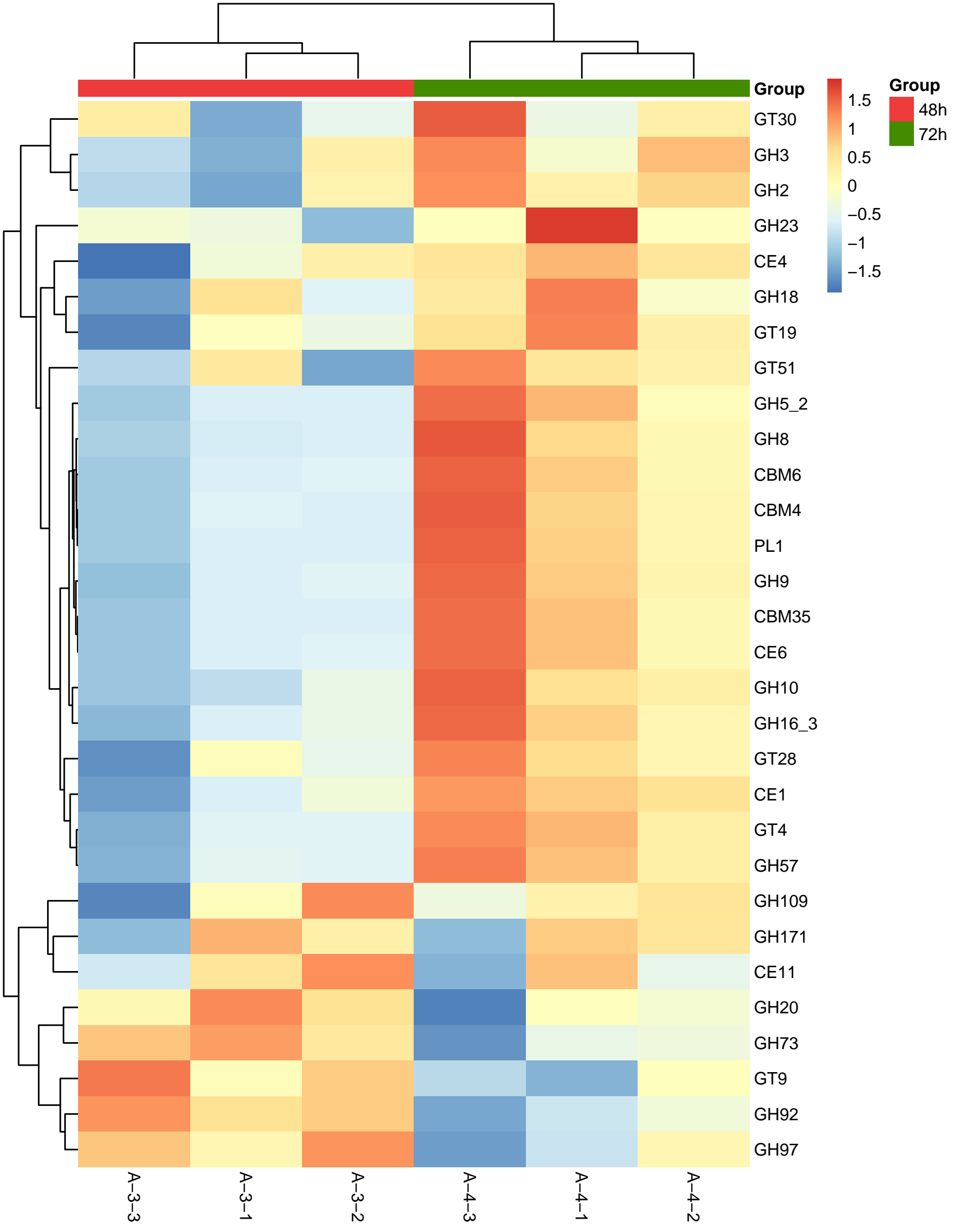

Supplement: Supplemental Information 3 [file peerj-13-20386-s003.zip › Raw data 3 Structural of microbial communities/Sample.CAZy.family.TPM.top30.heatmap.pdf]

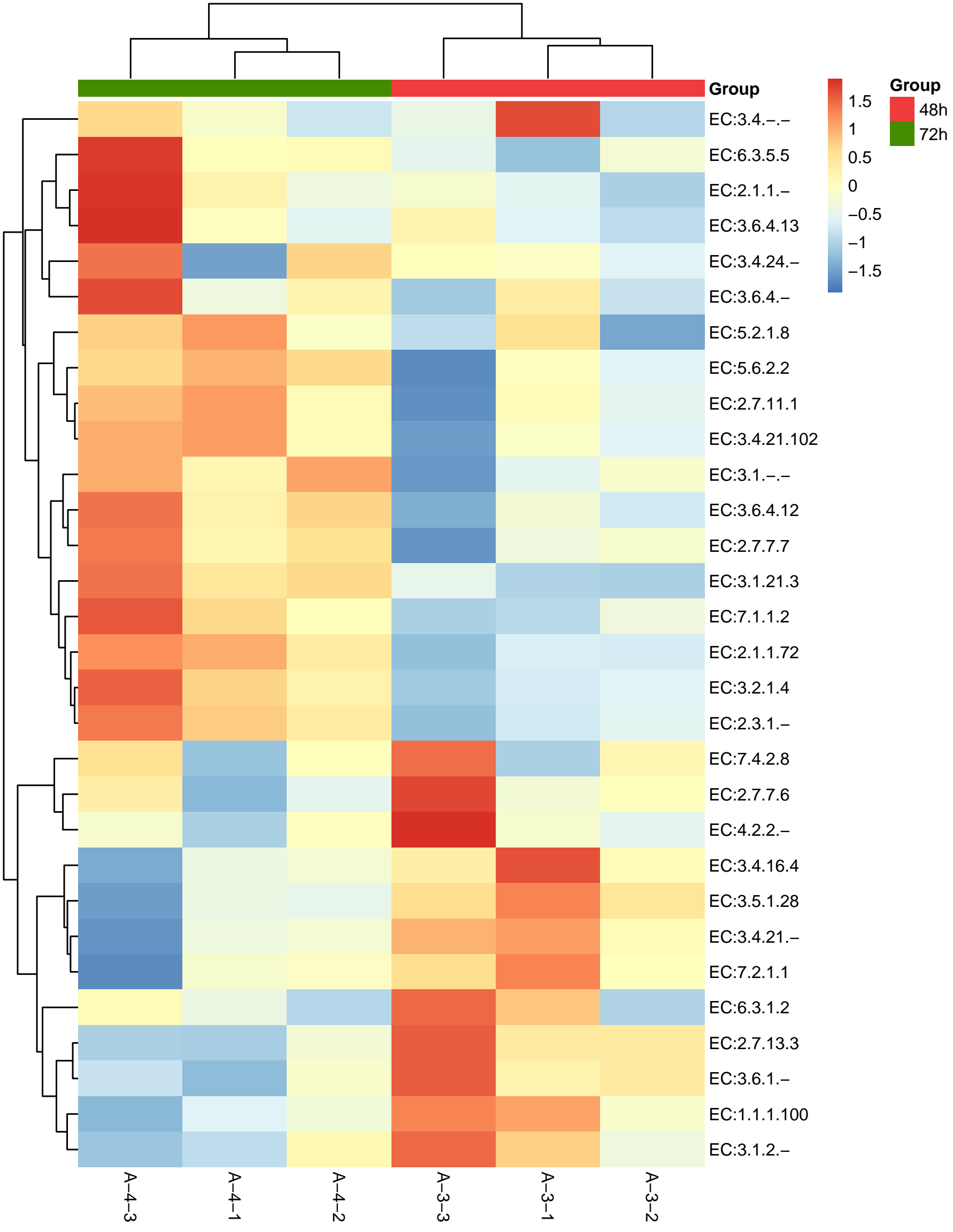

Supplement: Supplemental Information 3 [file peerj-13-20386-s003.zip › Raw data 3 Structural of microbial communities/Sample.KEGG.enzyme.TPM.top30.heatmap.pdf]

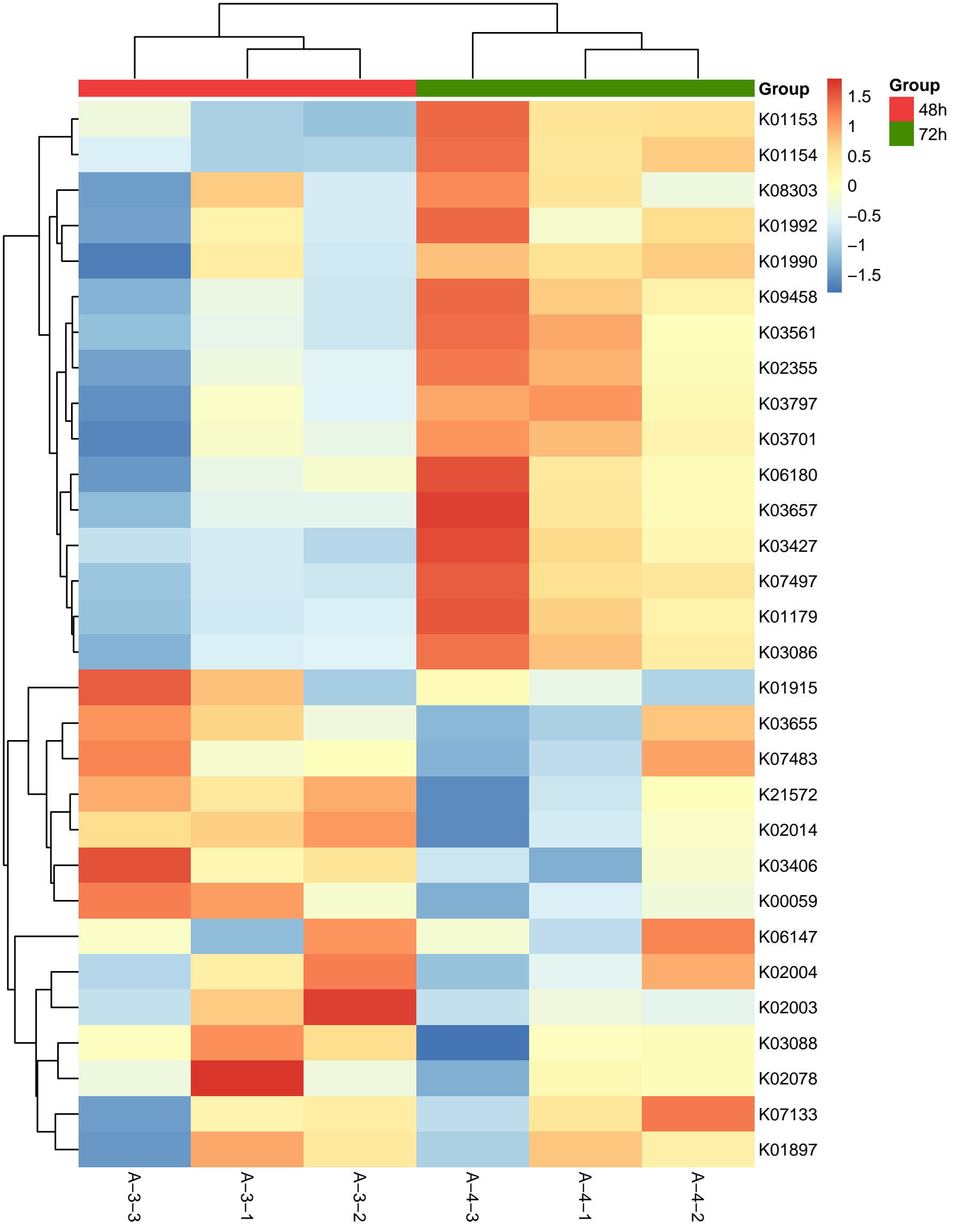

Supplement: Supplemental Information 3 [file peerj-13-20386-s003.zip › Raw data 3 Structural of microbial communities/Sample.KEGG.KO.TPM.top30.heatmap.pdf]

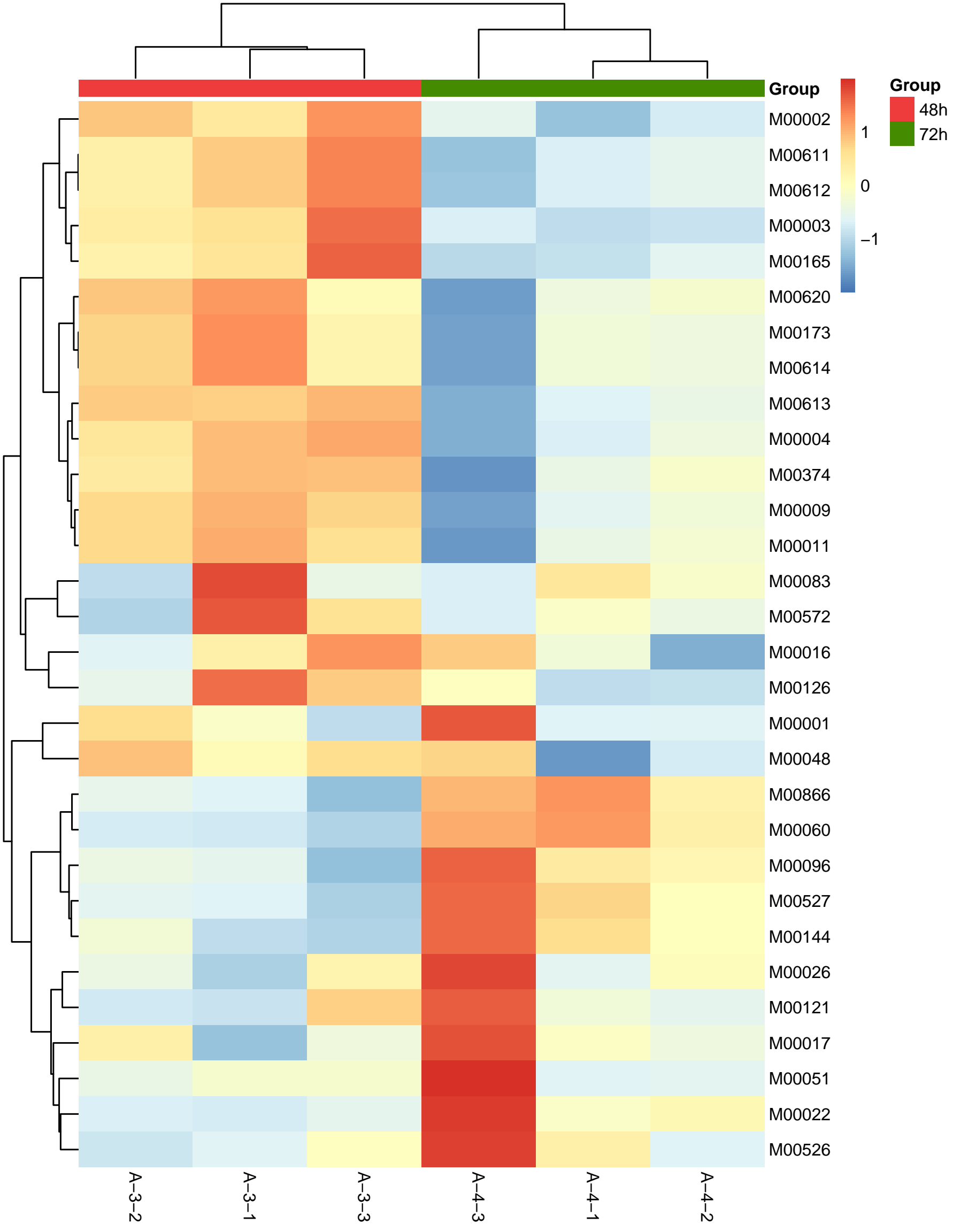

Supplement: Supplemental Information 3 [file peerj-13-20386-s003.zip › Raw data 3 Structural of microbial communities/Sample.KEGG.module.TPM.top30.heatmap.pdf]

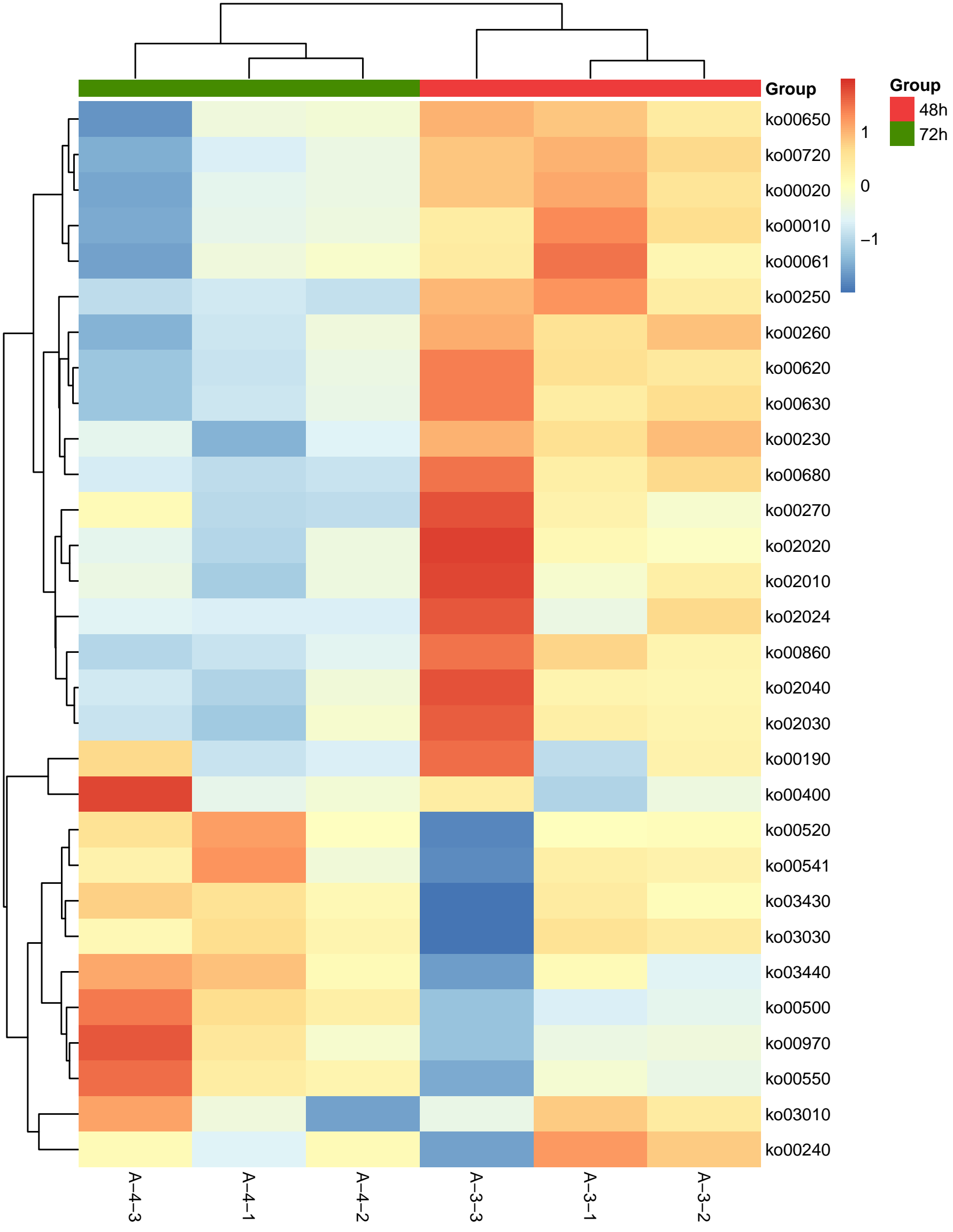

Supplement: Supplemental Information 3 [file peerj-13-20386-s003.zip › Raw data 3 Structural of microbial communities/Sample.KEGG.pathway.TPM.top30.heatmap.pdf]

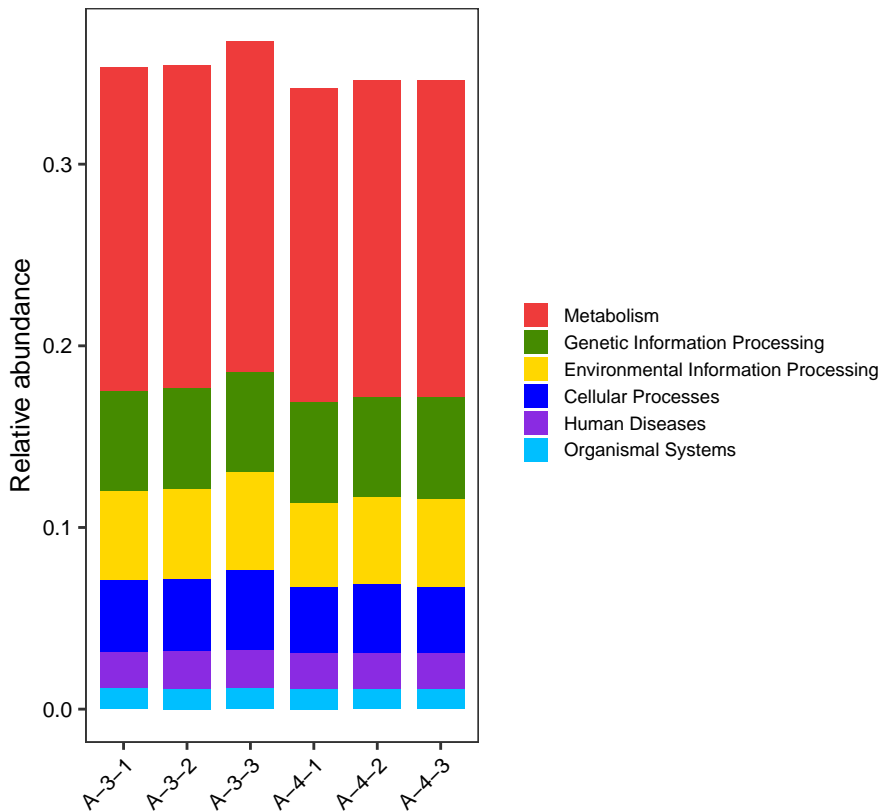

Supplement: Supplemental Information 3 [file peerj-13-20386-s003.zip › Raw data 3 Structural of microbial communities/Sample.KEGG.pathway_hierarchy1.percentage.all.histogram.pdf]

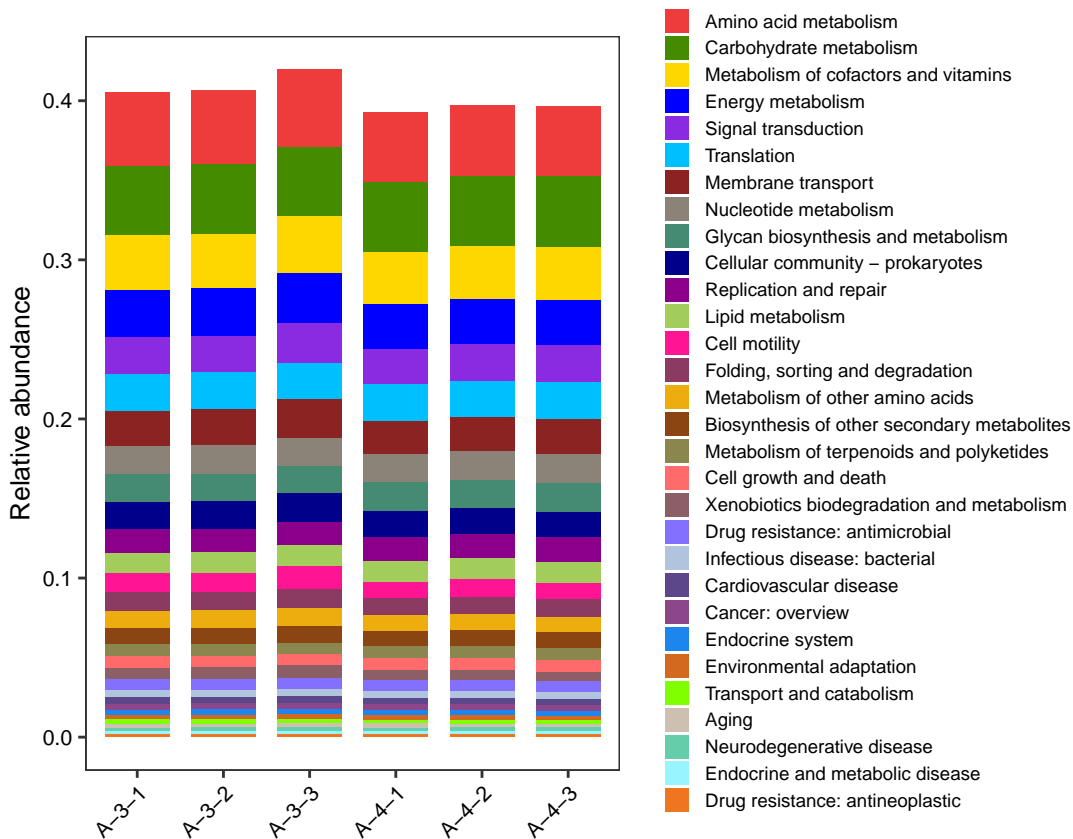

Supplement: Supplemental Information 3 [file peerj-13-20386-s003.zip › Raw data 3 Structural of microbial communities/Sample.KEGG.pathway_hierarchy2.percentage.top30.histogram.pdf]

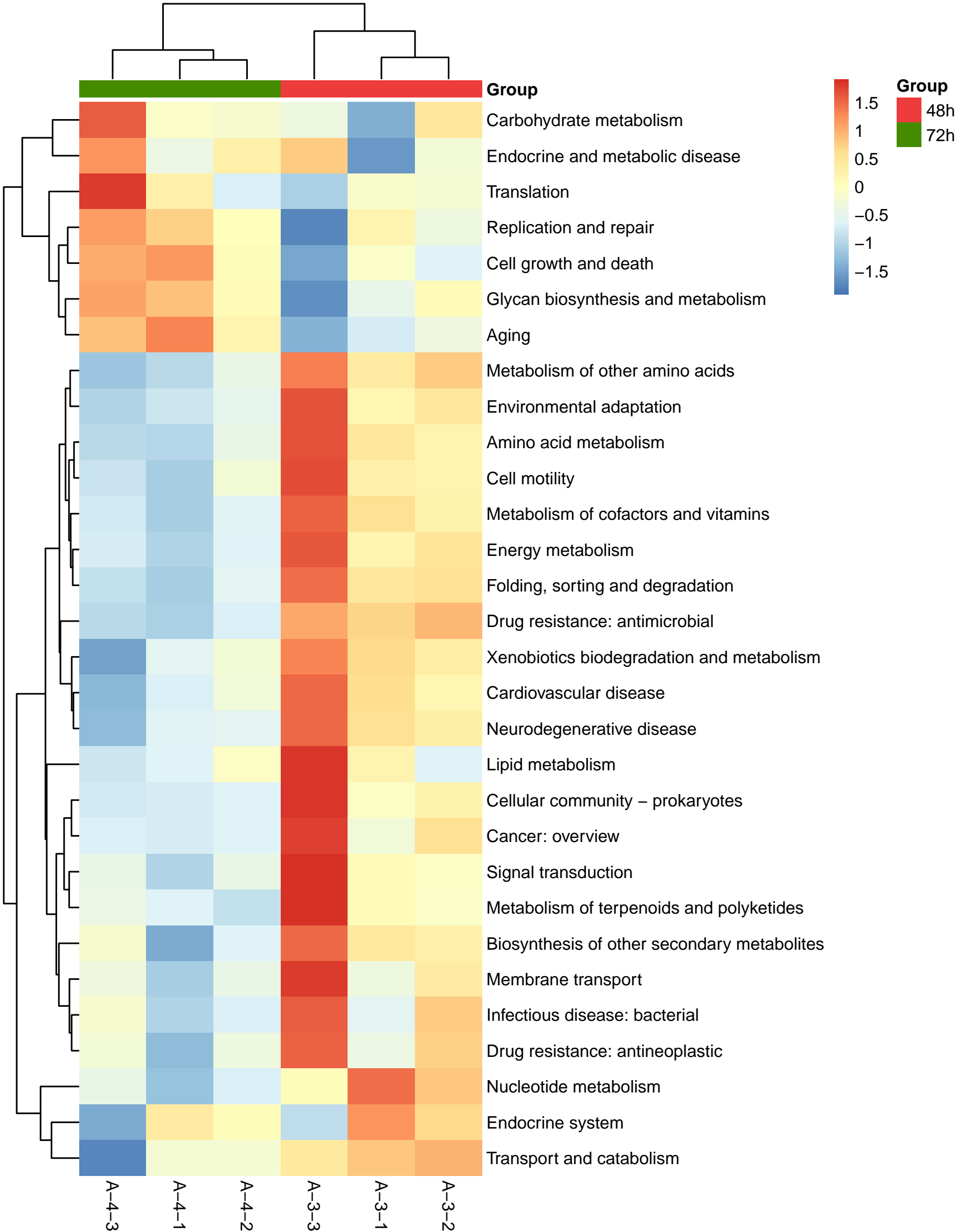

Supplement: Supplemental Information 3 [file peerj-13-20386-s003.zip › Raw data 3 Structural of microbial communities/Sample.KEGG.pathway_hierarchy2.TPM.top30.heatmap.pdf]

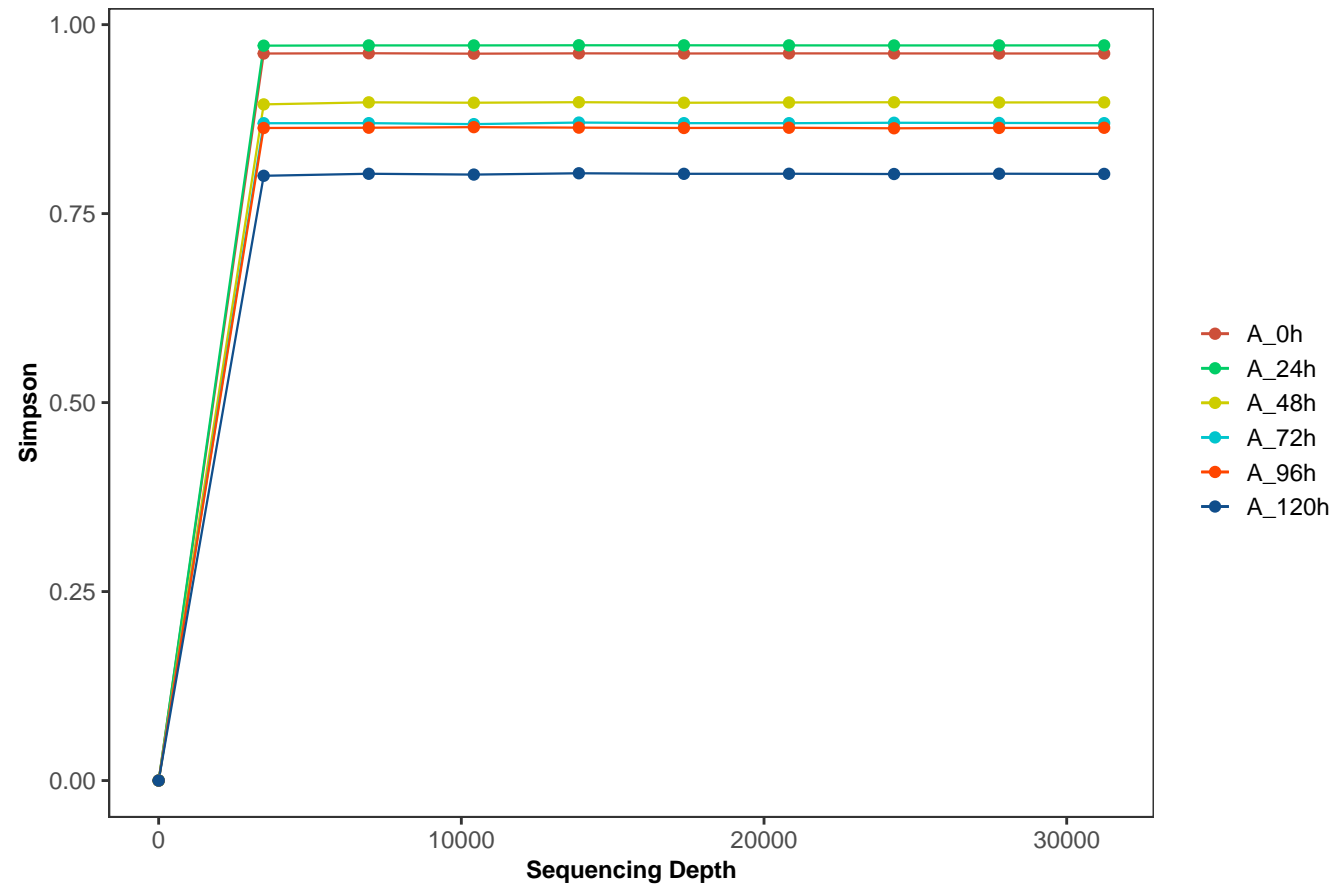

Supplement: Supplemental Information 3 [file peerj-13-20386-s003.zip › Raw data 3 Structural of microbial communities/Simpson_rare_curve_errorbar.group.pdf]

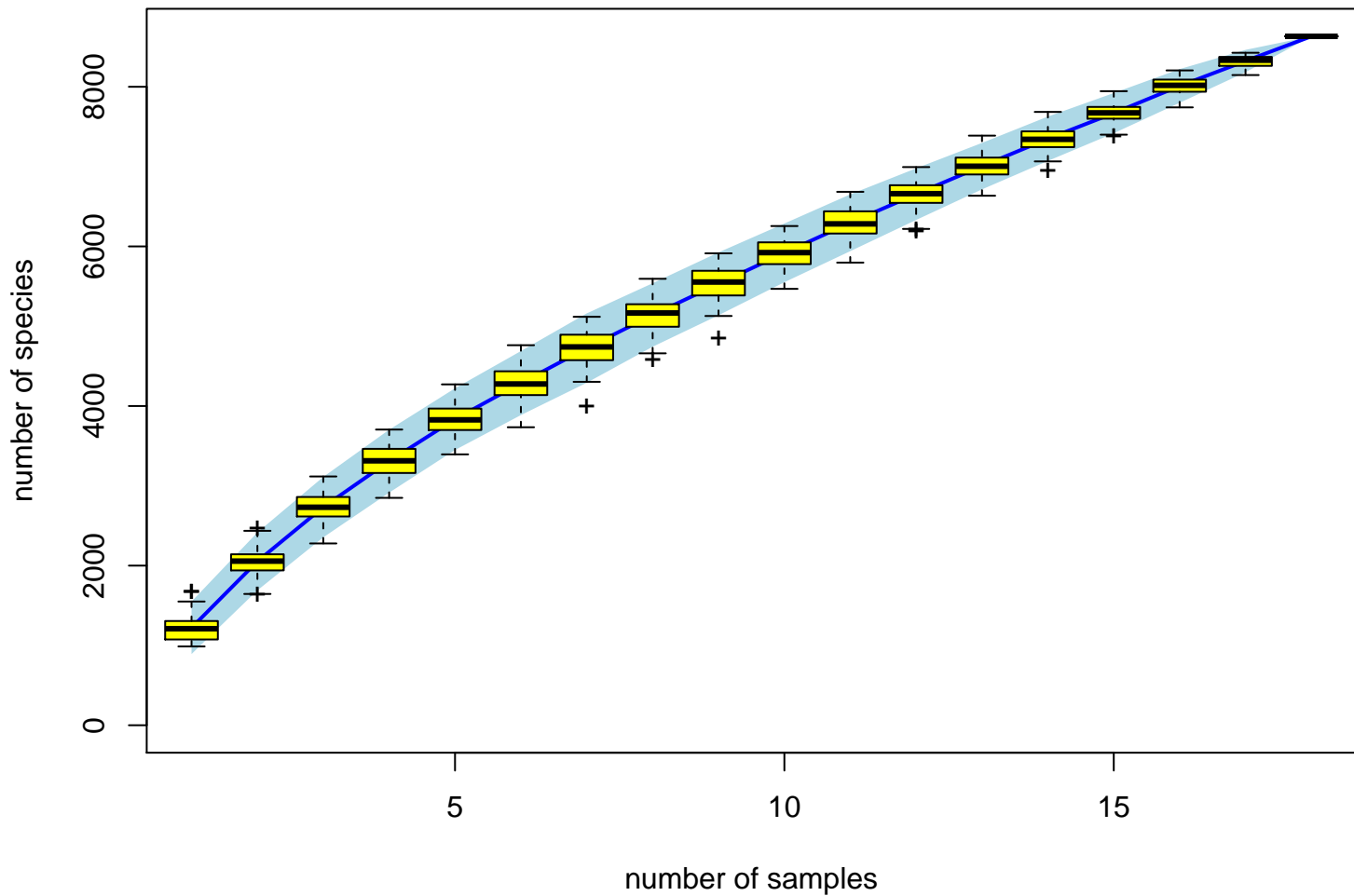

Supplement: Supplemental Information 3 [file peerj-13-20386-s003.zip › Raw data 3 Structural of microbial communities/specaccum.pdf]

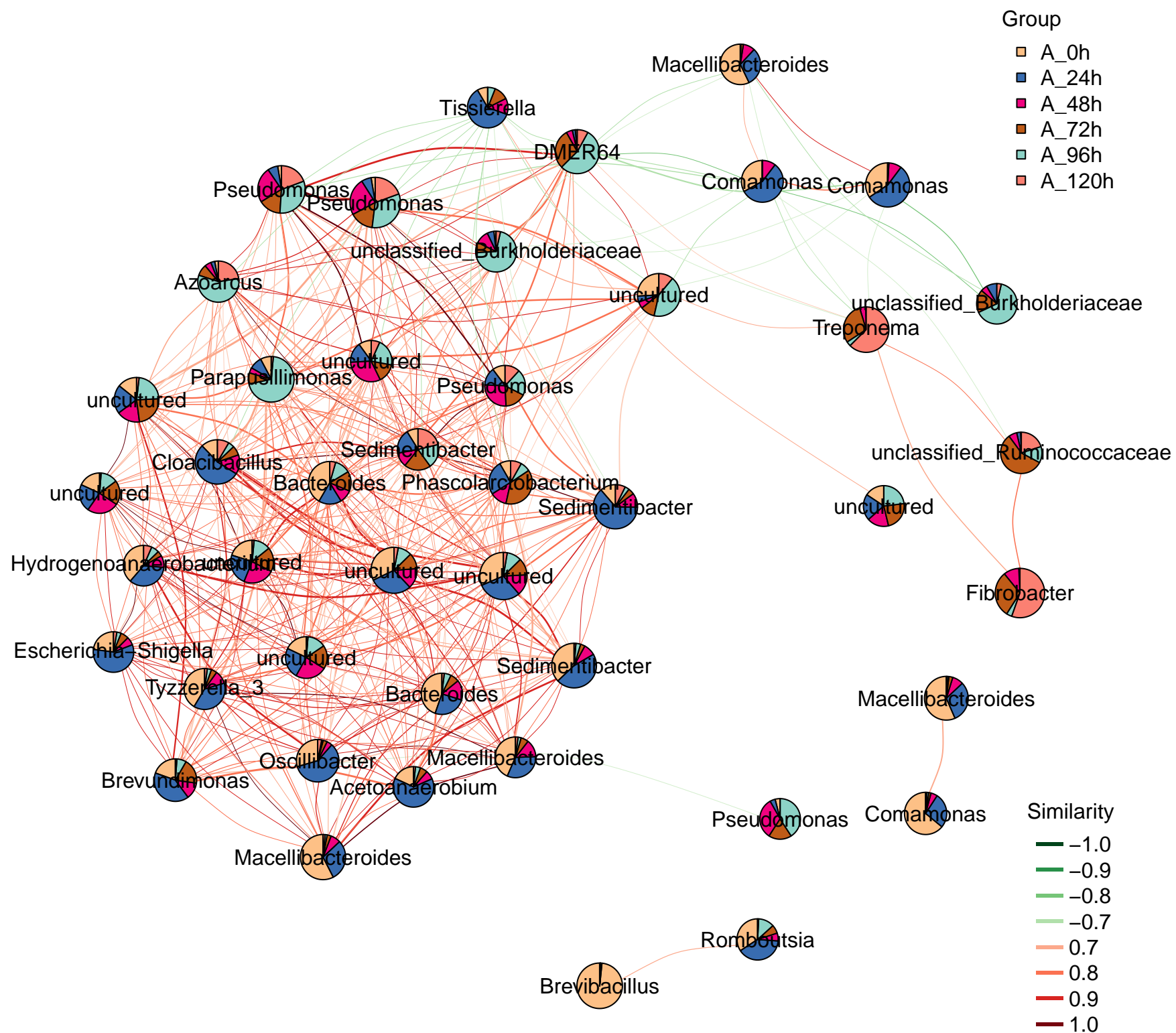

Supplement: Supplemental Information 3 [file peerj-13-20386-s003.zip › Raw data 3 Structural of microbial communities/subnetwork_group_top50.pdf]

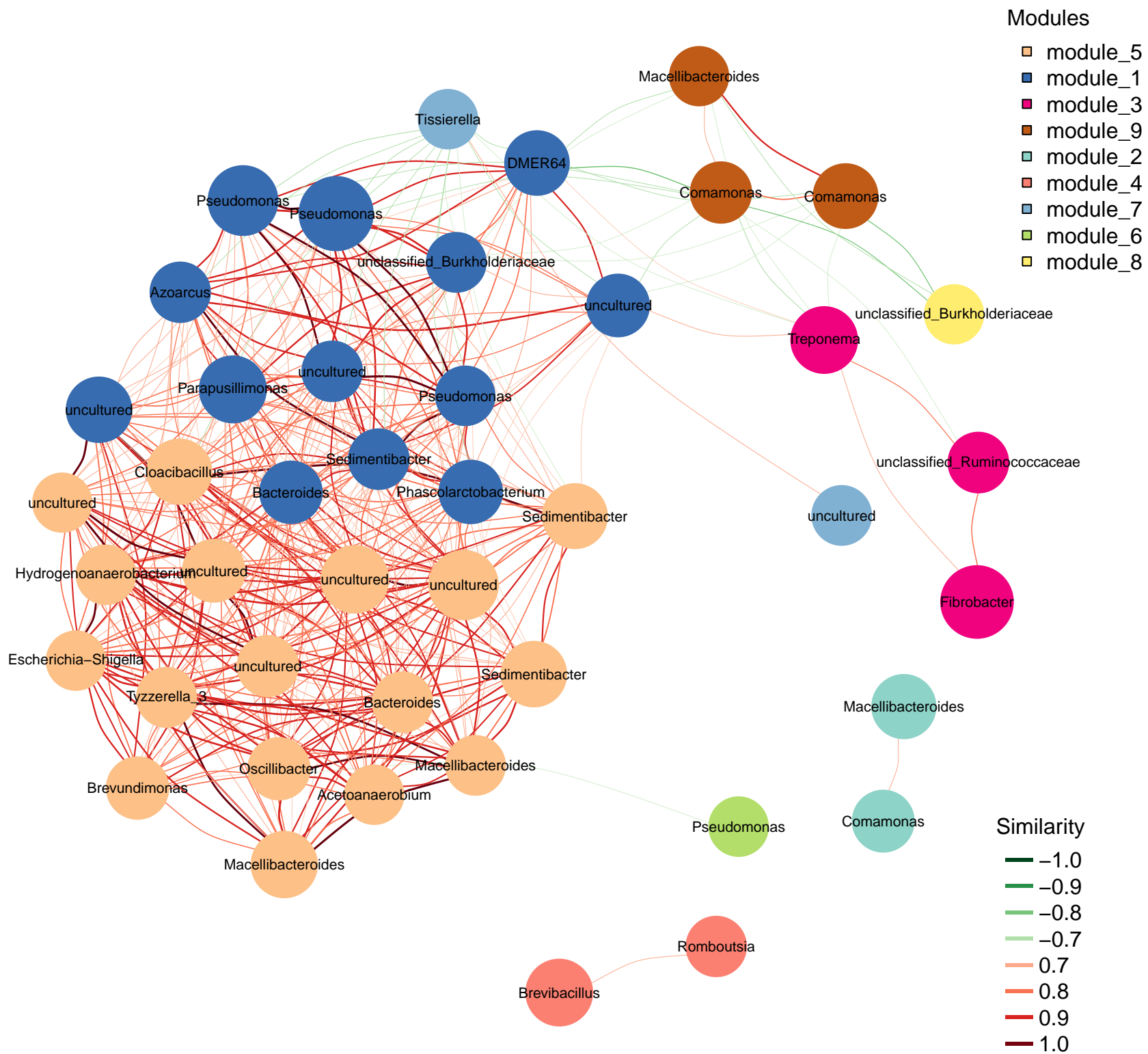

Supplement: Supplemental Information 3 [file peerj-13-20386-s003.zip › Raw data 3 Structural of microbial communities/subnetwork_module_top50.pdf]

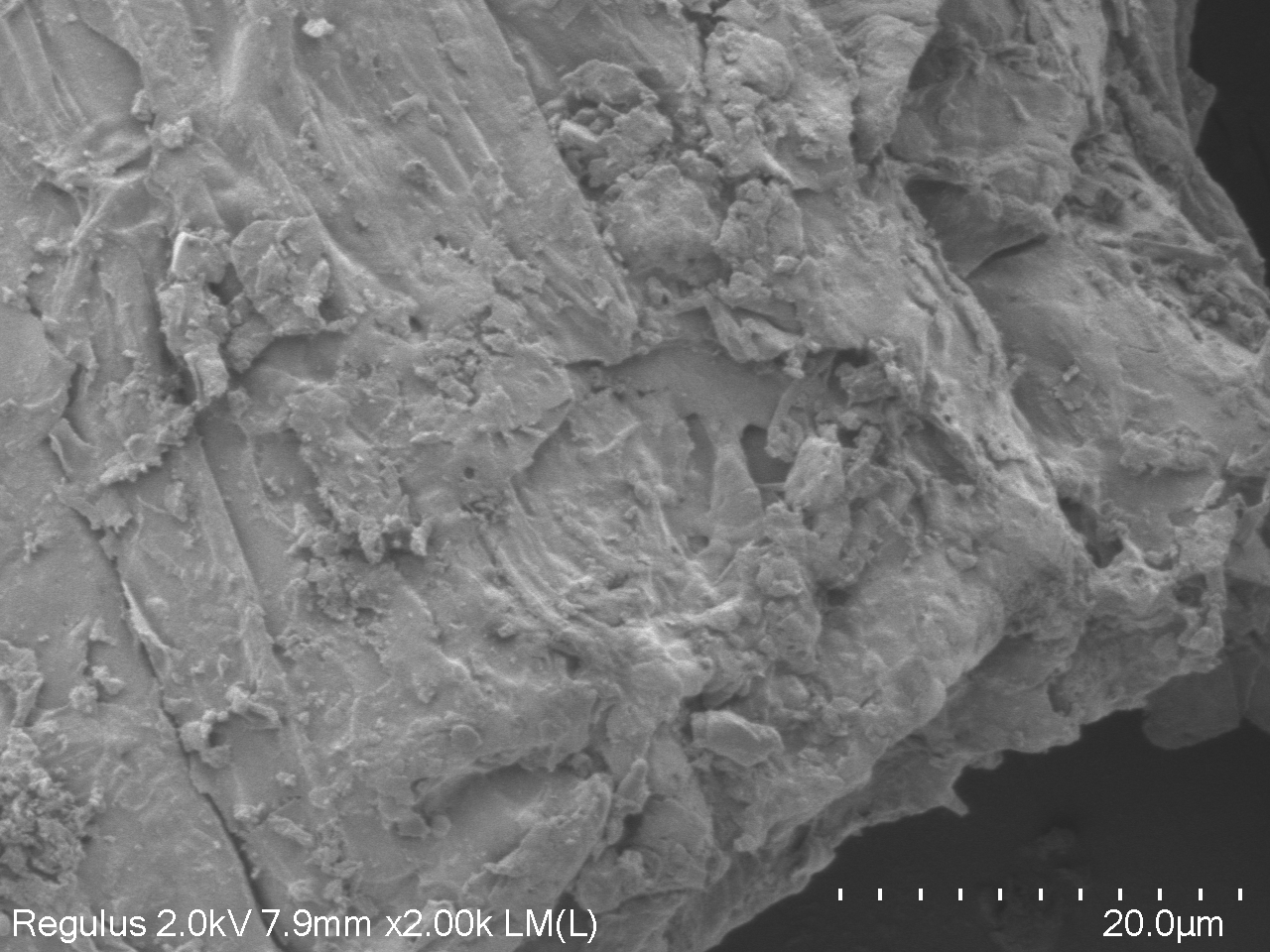

Supplement: Supplemental Information 4 [file peerj-13-20386-s004.zip › 3D/3D-1.tif]

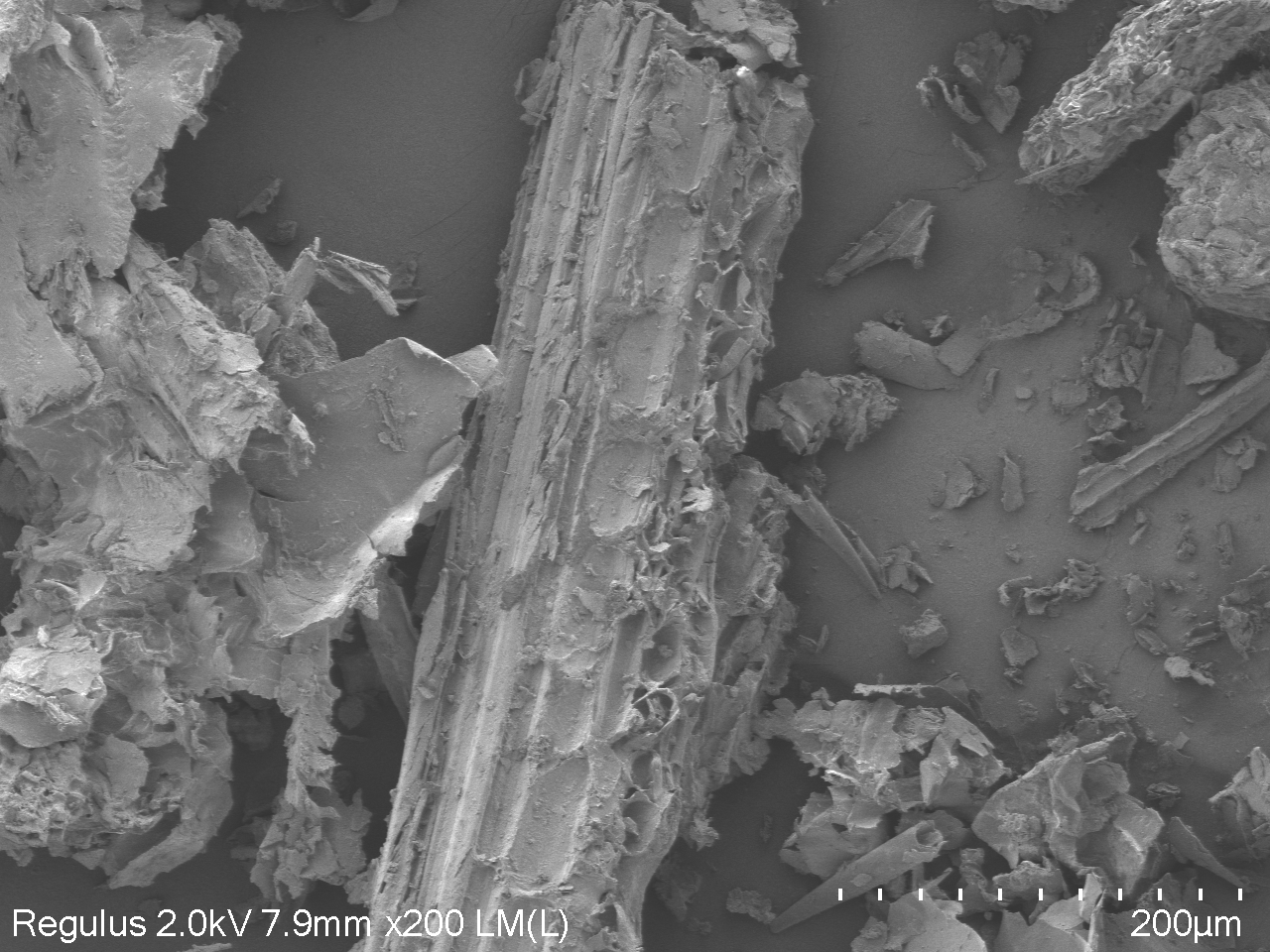

Supplement: Supplemental Information 4 [file peerj-13-20386-s004.zip › 3D/3D-10.tif]

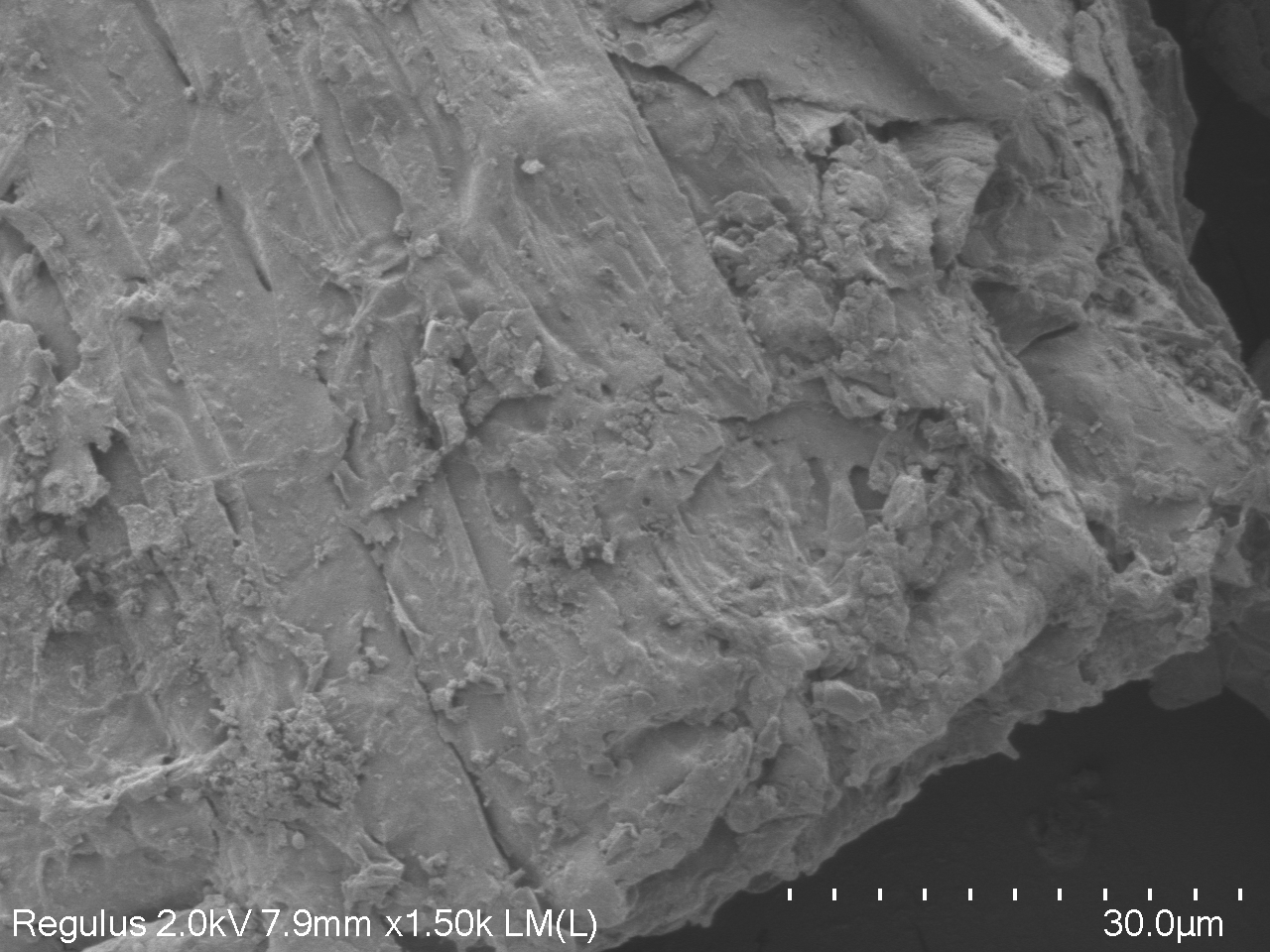

Supplement: Supplemental Information 4 [file peerj-13-20386-s004.zip › 3D/3D-2.tif]

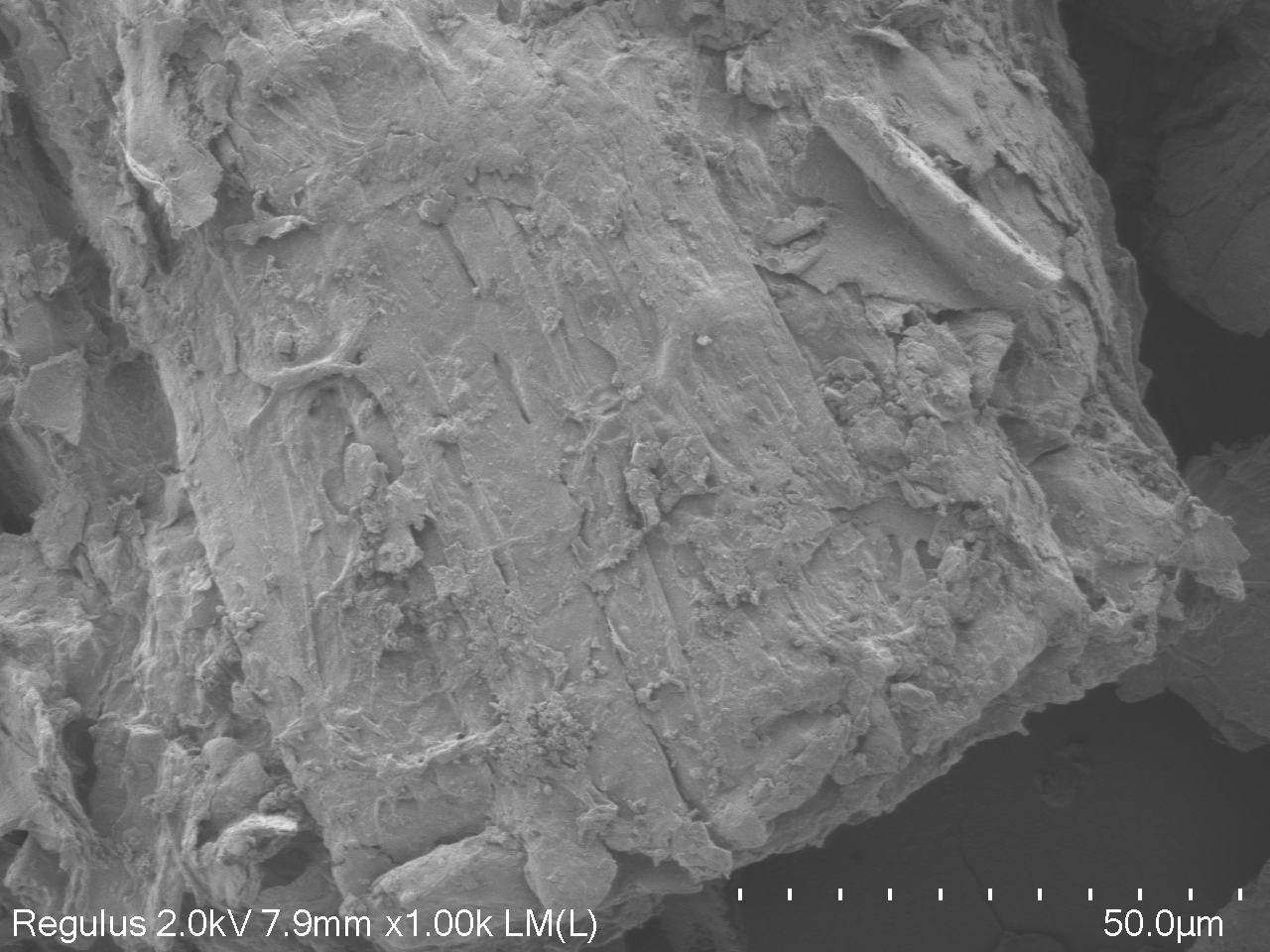

Supplement: Supplemental Information 4 [file peerj-13-20386-s004.zip › 3D/3D-3.tif]

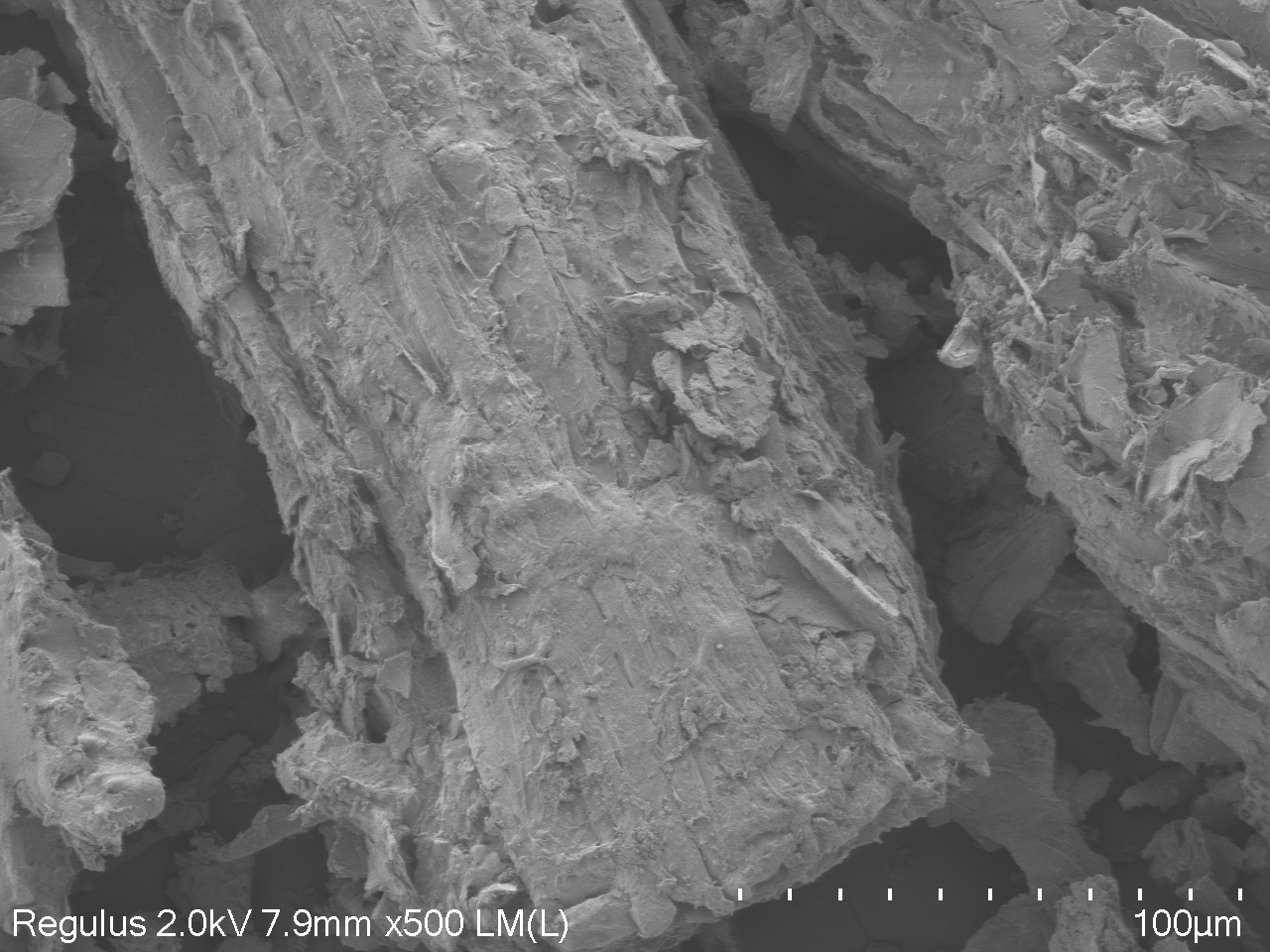

Supplement: Supplemental Information 4 [file peerj-13-20386-s004.zip › 3D/3D-4.tif]

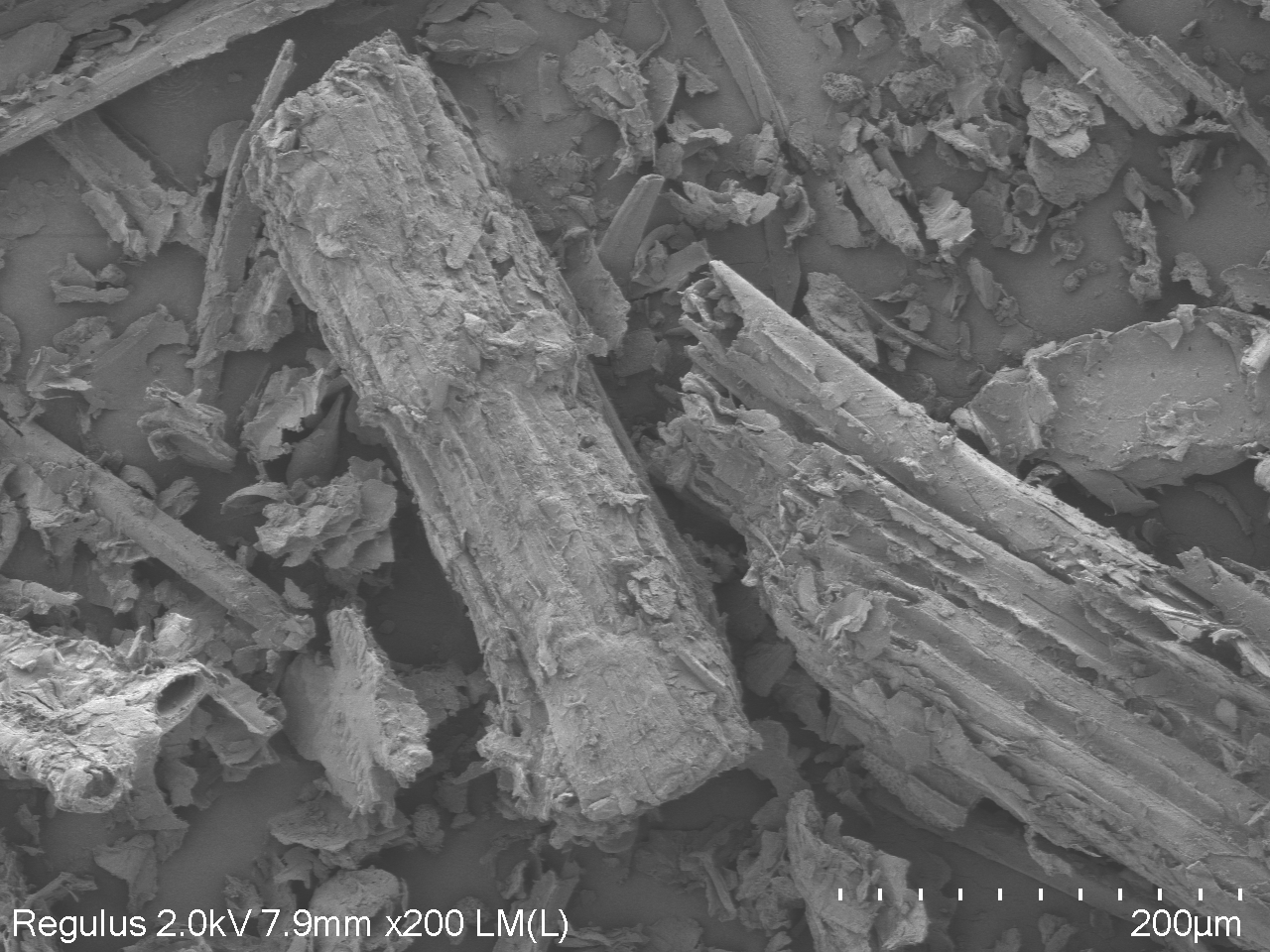

Supplement: Supplemental Information 4 [file peerj-13-20386-s004.zip › 3D/3D-5.tif]

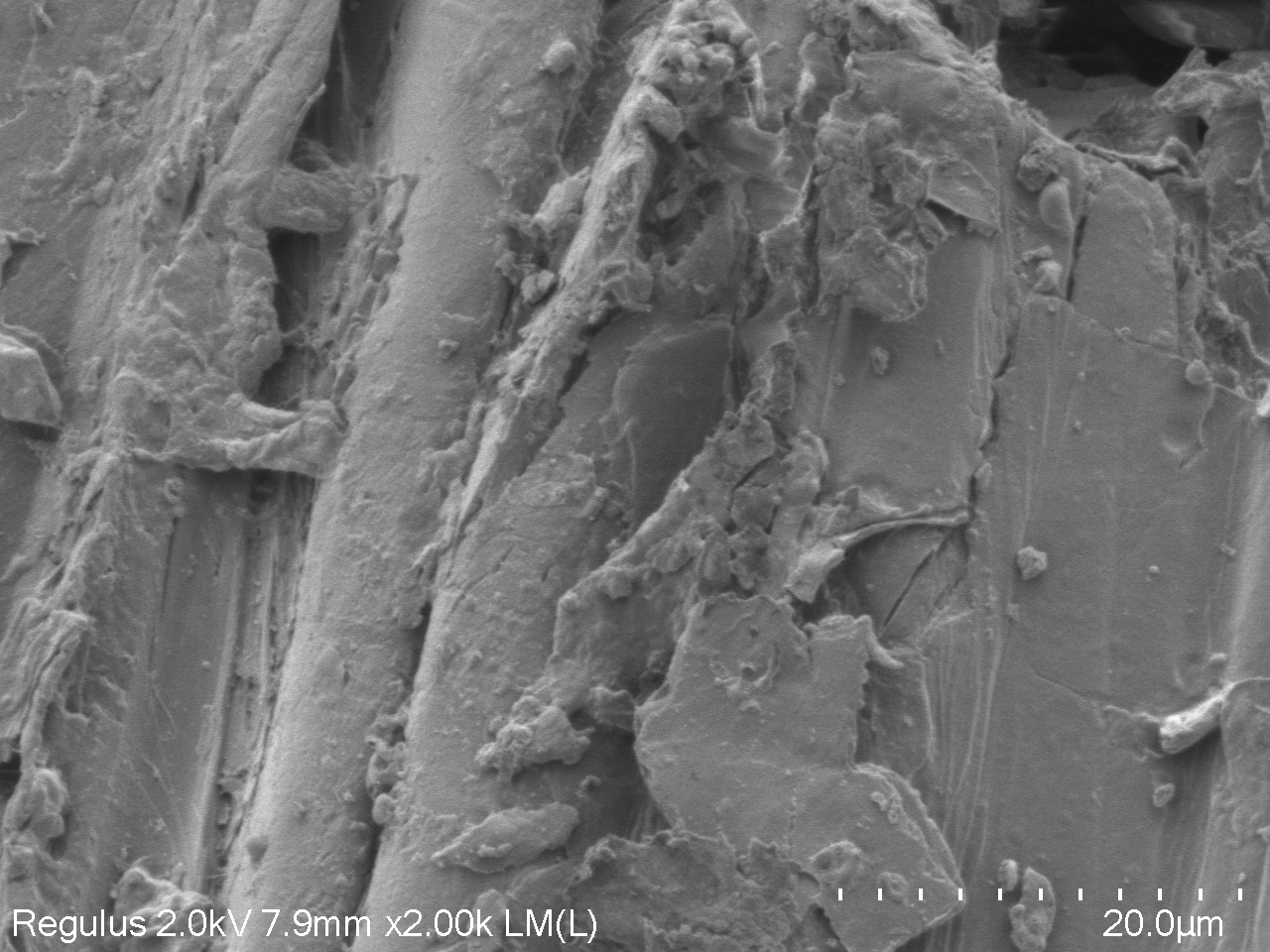

Supplement: Supplemental Information 4 [file peerj-13-20386-s004.zip › 3D/3D-6.tif]

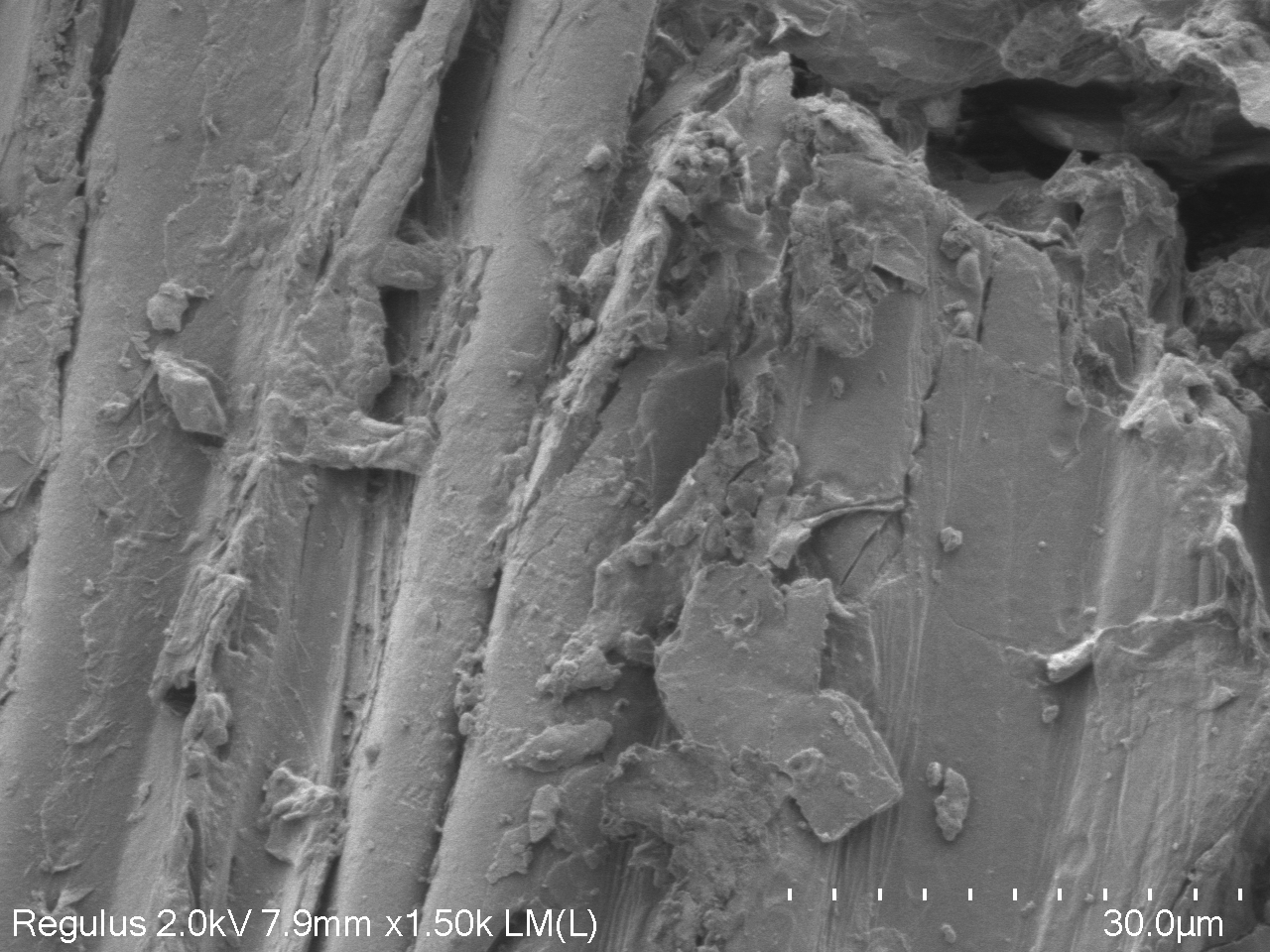

Supplement: Supplemental Information 4 [file peerj-13-20386-s004.zip › 3D/3D-7.tif]

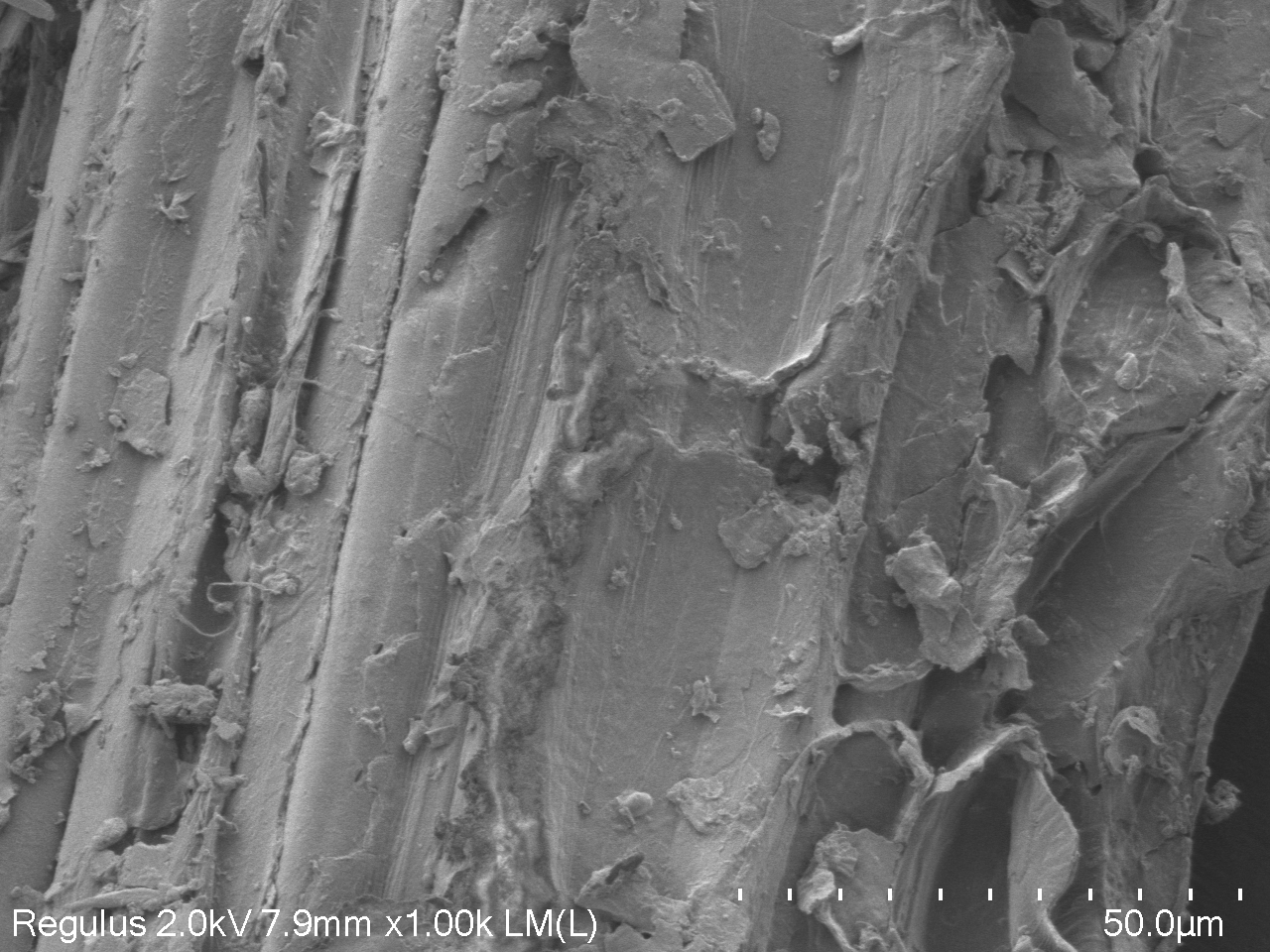

Supplement: Supplemental Information 4 [file peerj-13-20386-s004.zip › 3D/3D-8.tif]

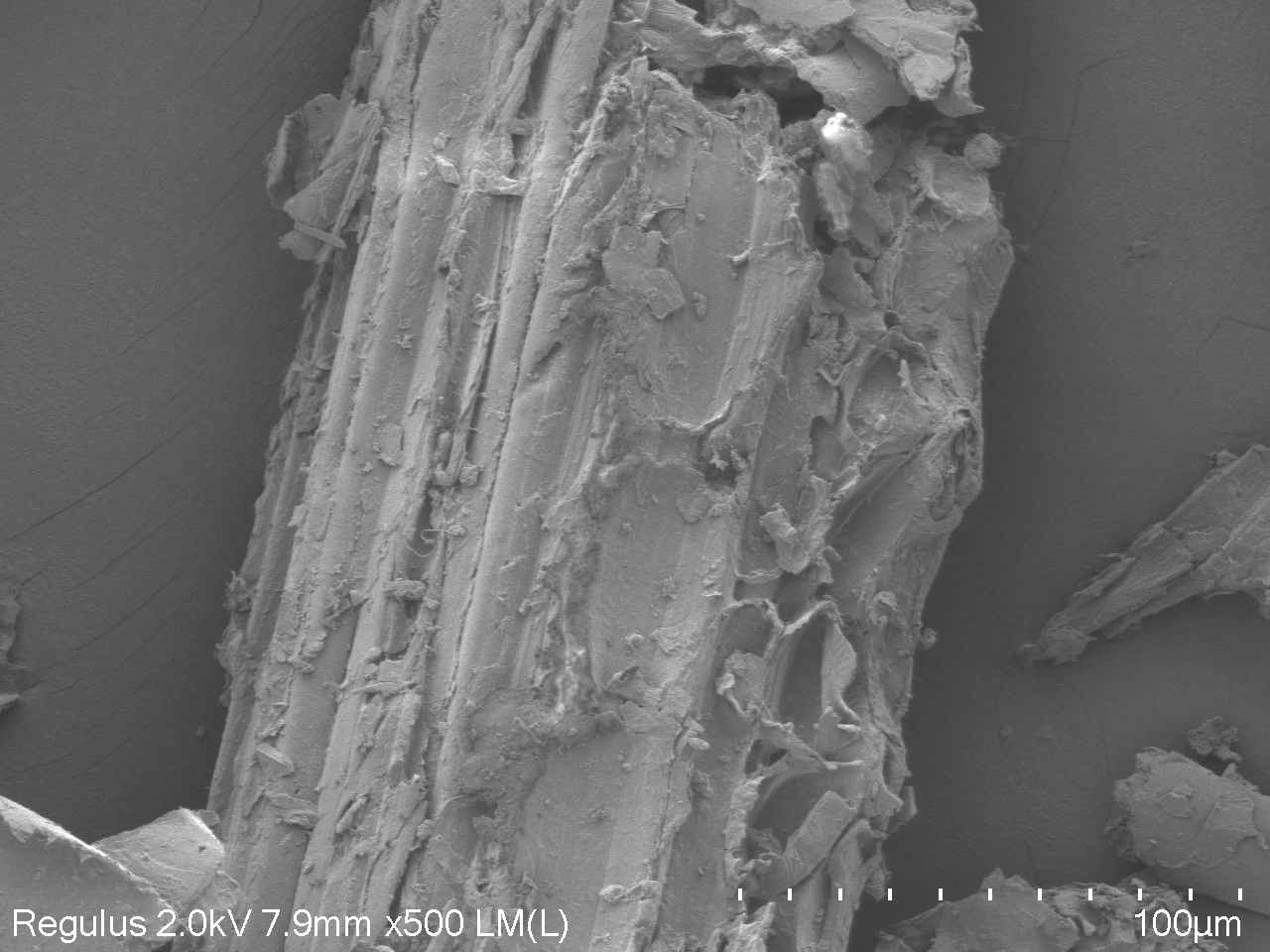

Supplement: Supplemental Information 4 [file peerj-13-20386-s004.zip › 3D/3D-9.tif]

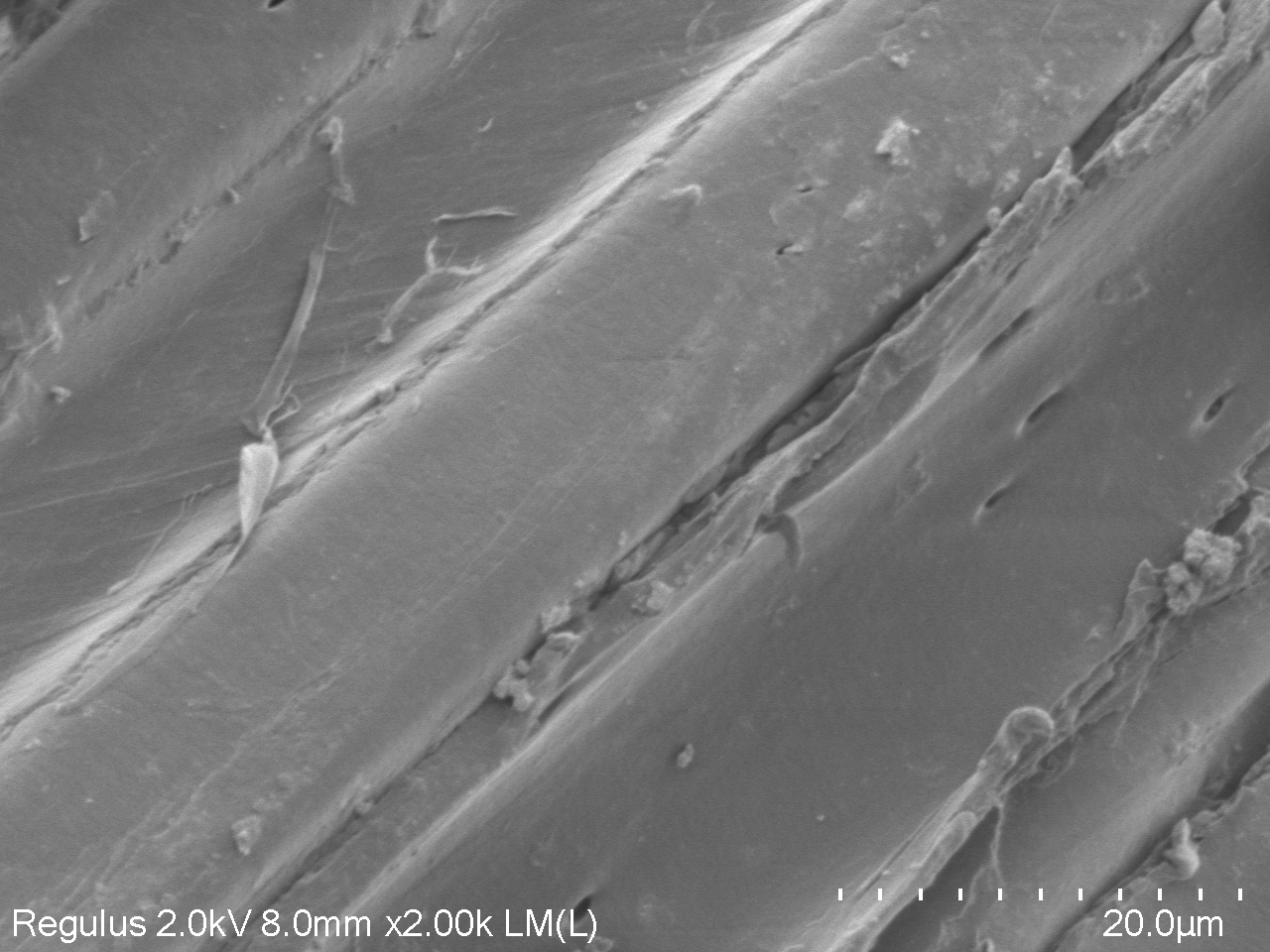

Supplement: Supplemental Information 5 [file peerj-13-20386-s005.zip › 1SEM1124/1-1.tif]

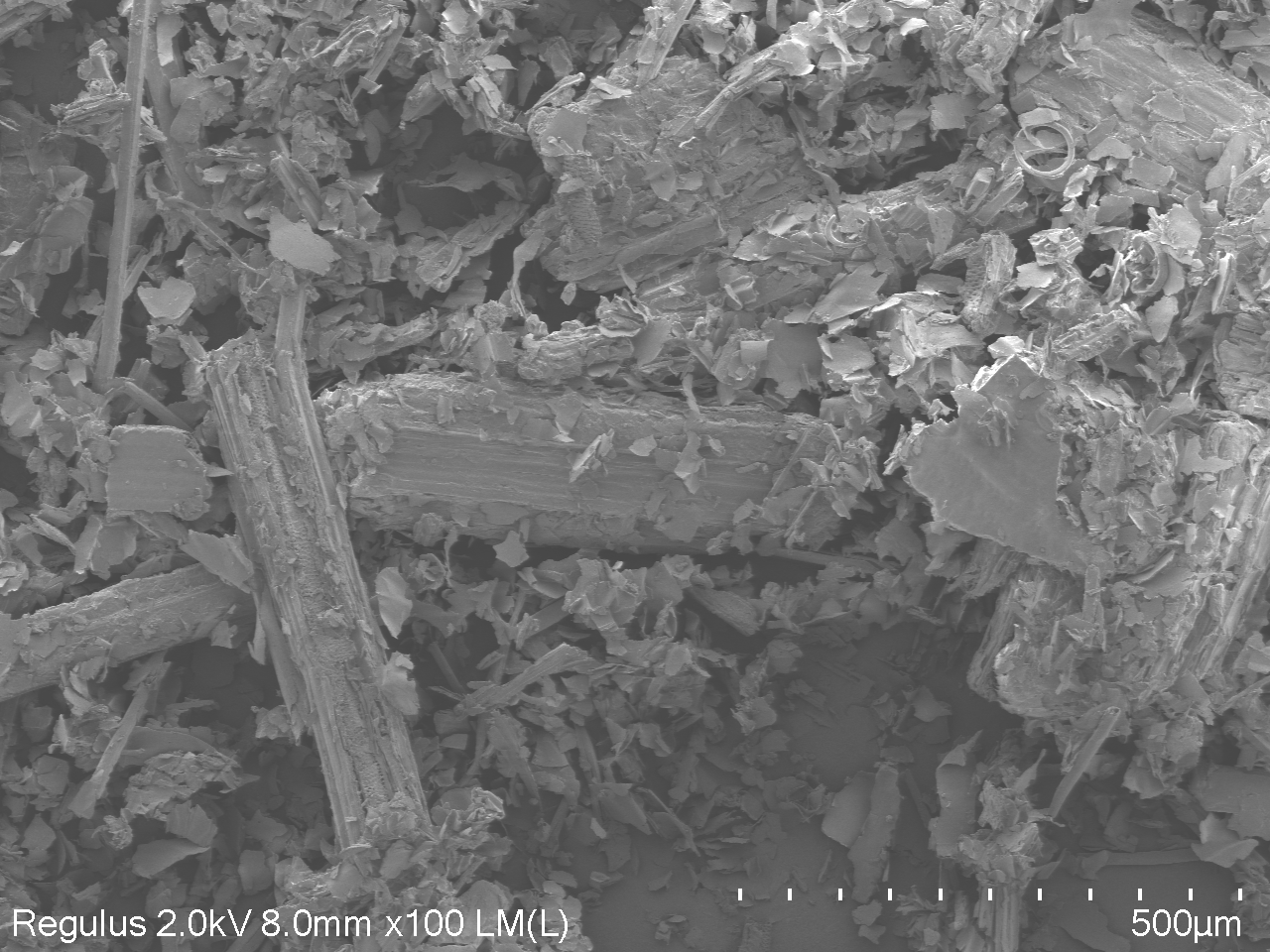

Supplement: Supplemental Information 5 [file peerj-13-20386-s005.zip › 1SEM1124/1-10.tif]

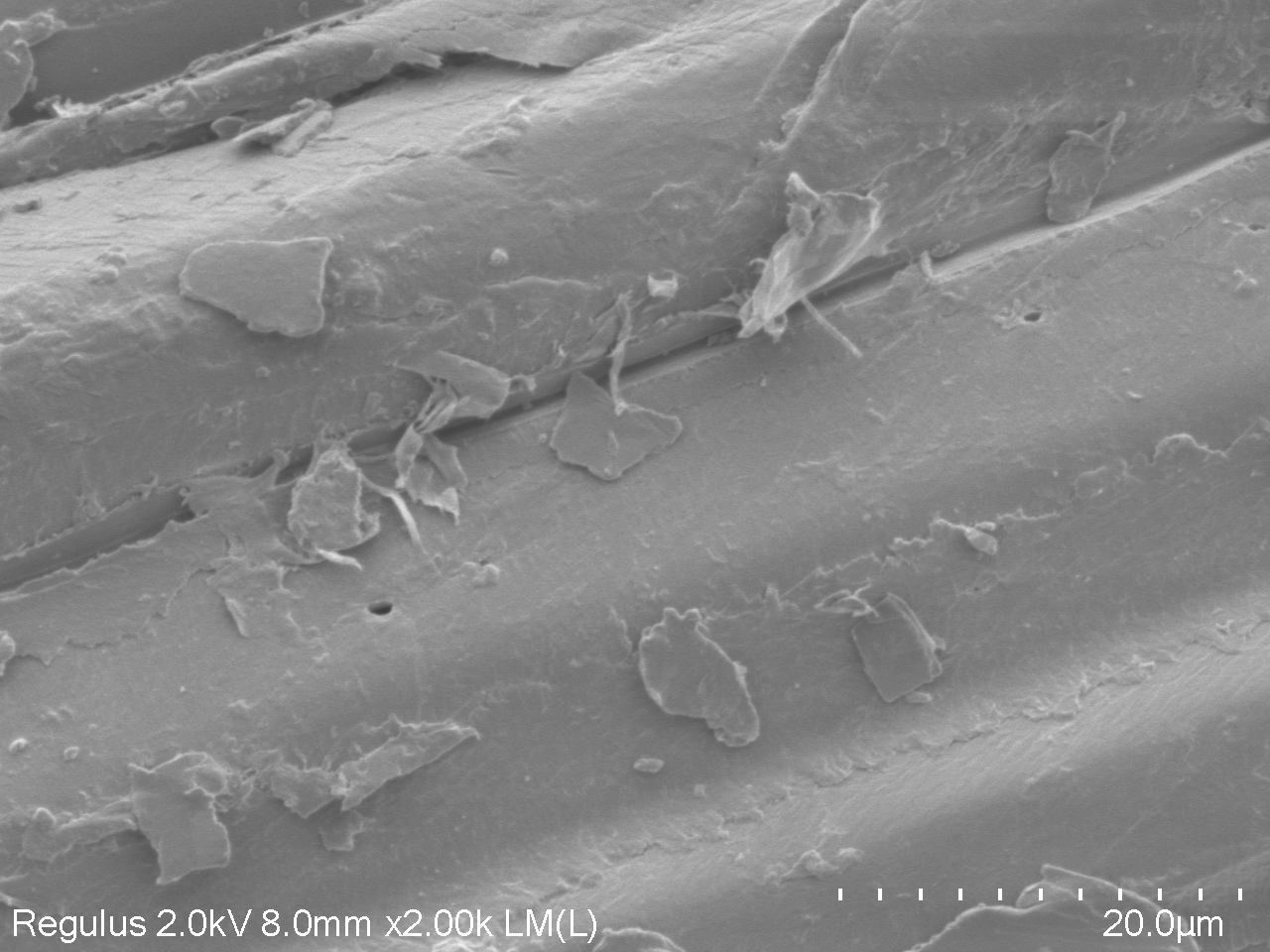

Supplement: Supplemental Information 5 [file peerj-13-20386-s005.zip › 1SEM1124/1-11.tif]

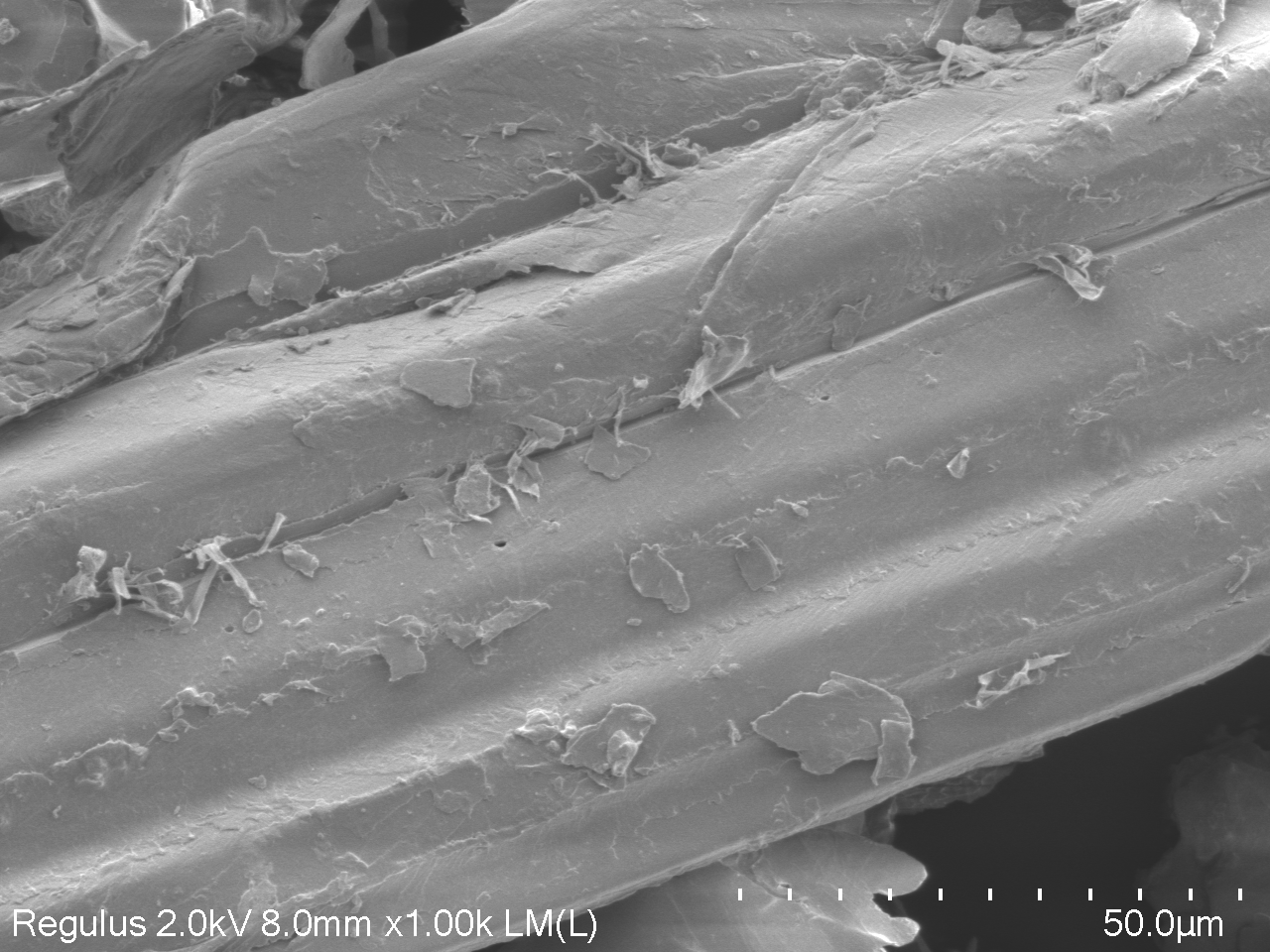

Supplement: Supplemental Information 5 [file peerj-13-20386-s005.zip › 1SEM1124/1-12.tif]

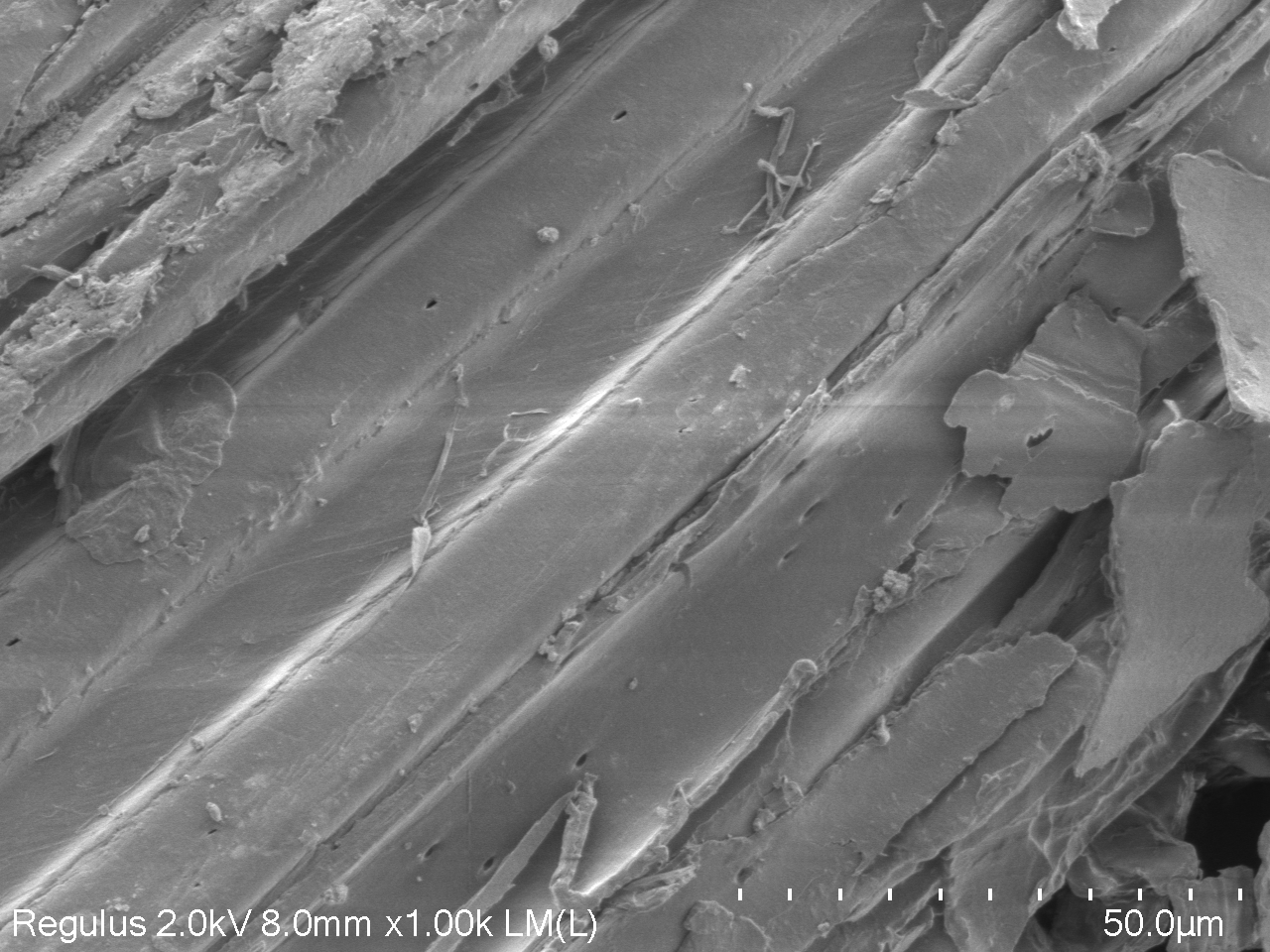

Supplement: Supplemental Information 5 [file peerj-13-20386-s005.zip › 1SEM1124/1-2.tif]

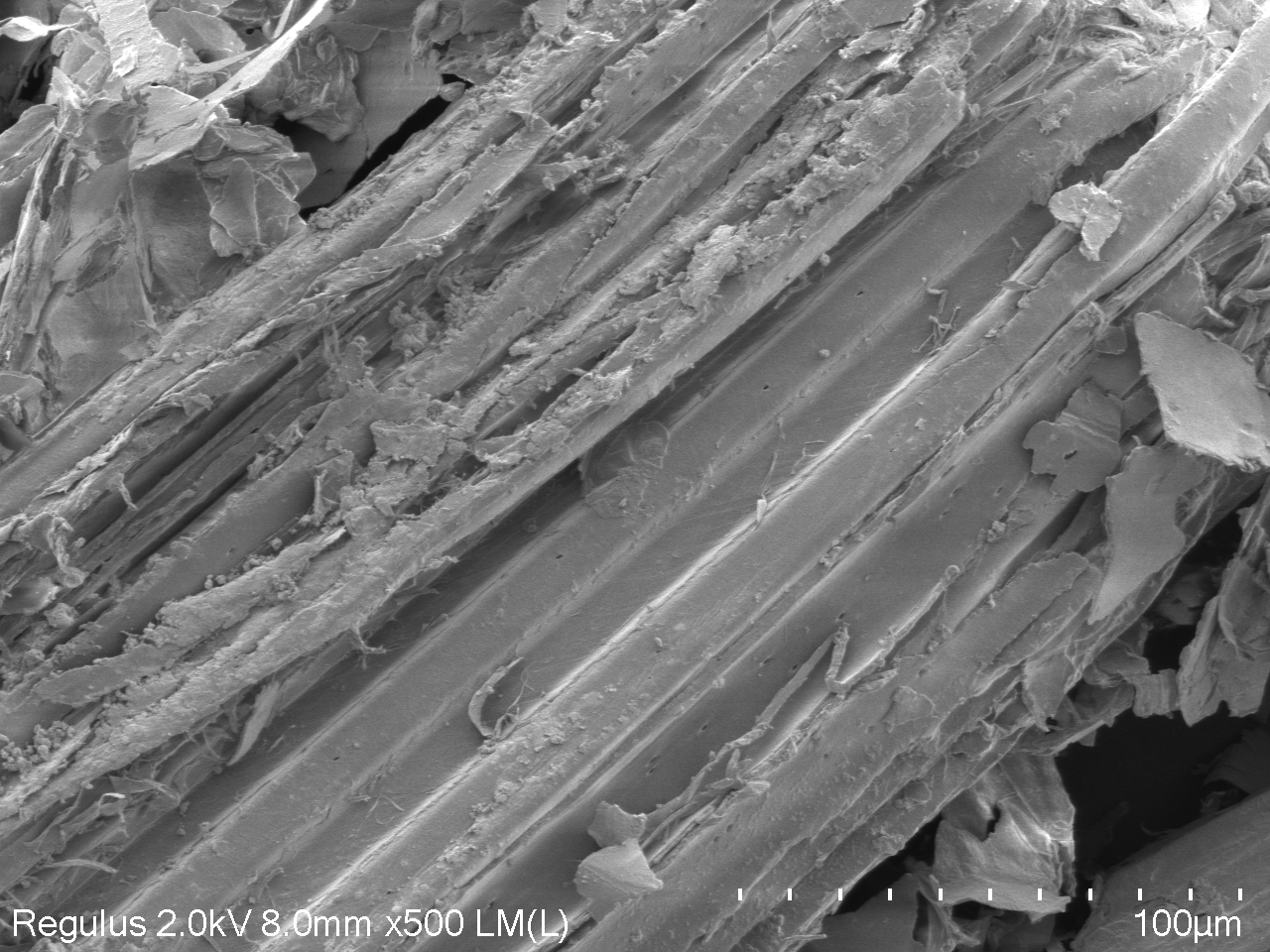

Supplement: Supplemental Information 5 [file peerj-13-20386-s005.zip › 1SEM1124/1-3.tif]

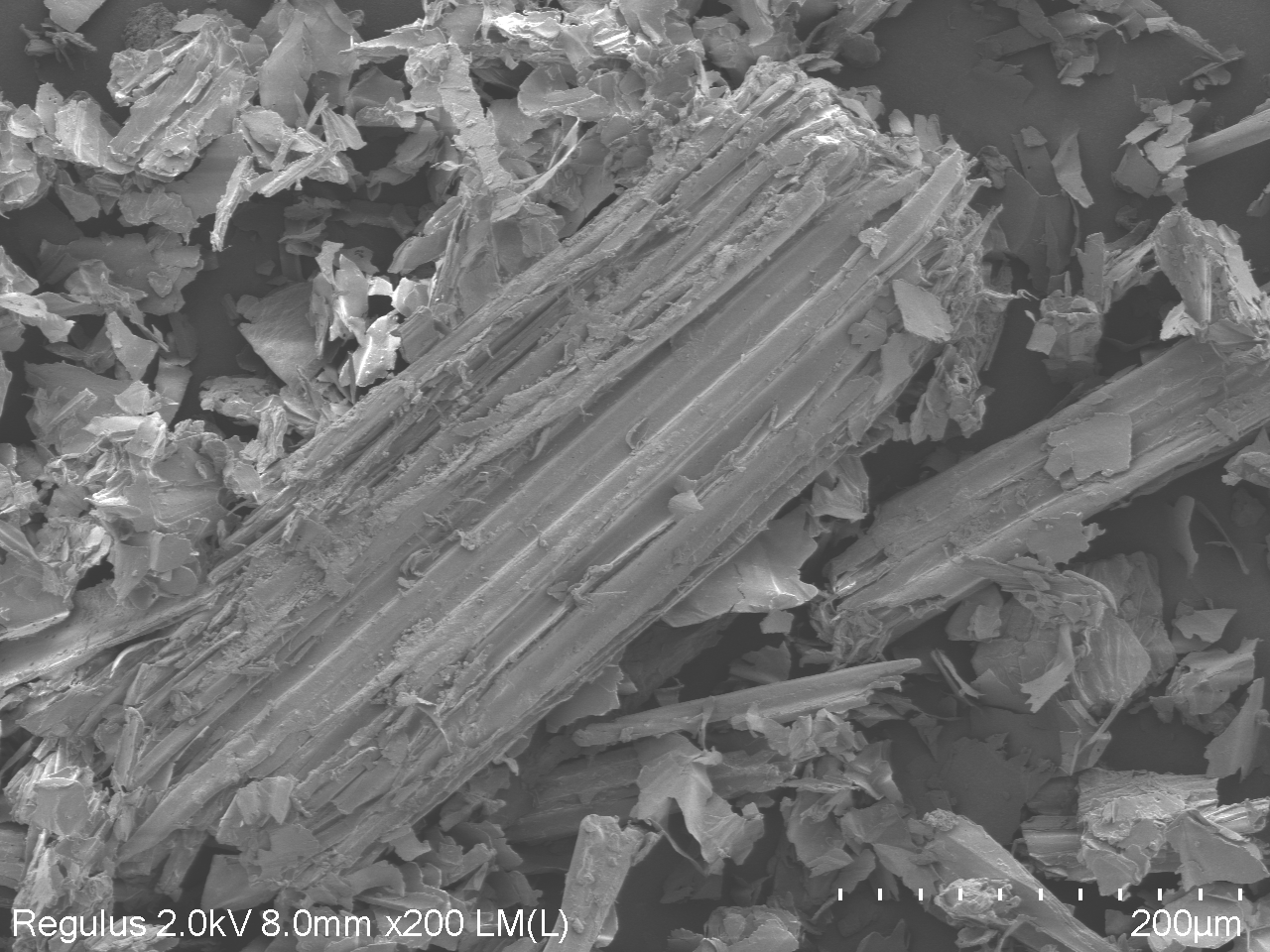

Supplement: Supplemental Information 5 [file peerj-13-20386-s005.zip › 1SEM1124/1-4.tif]

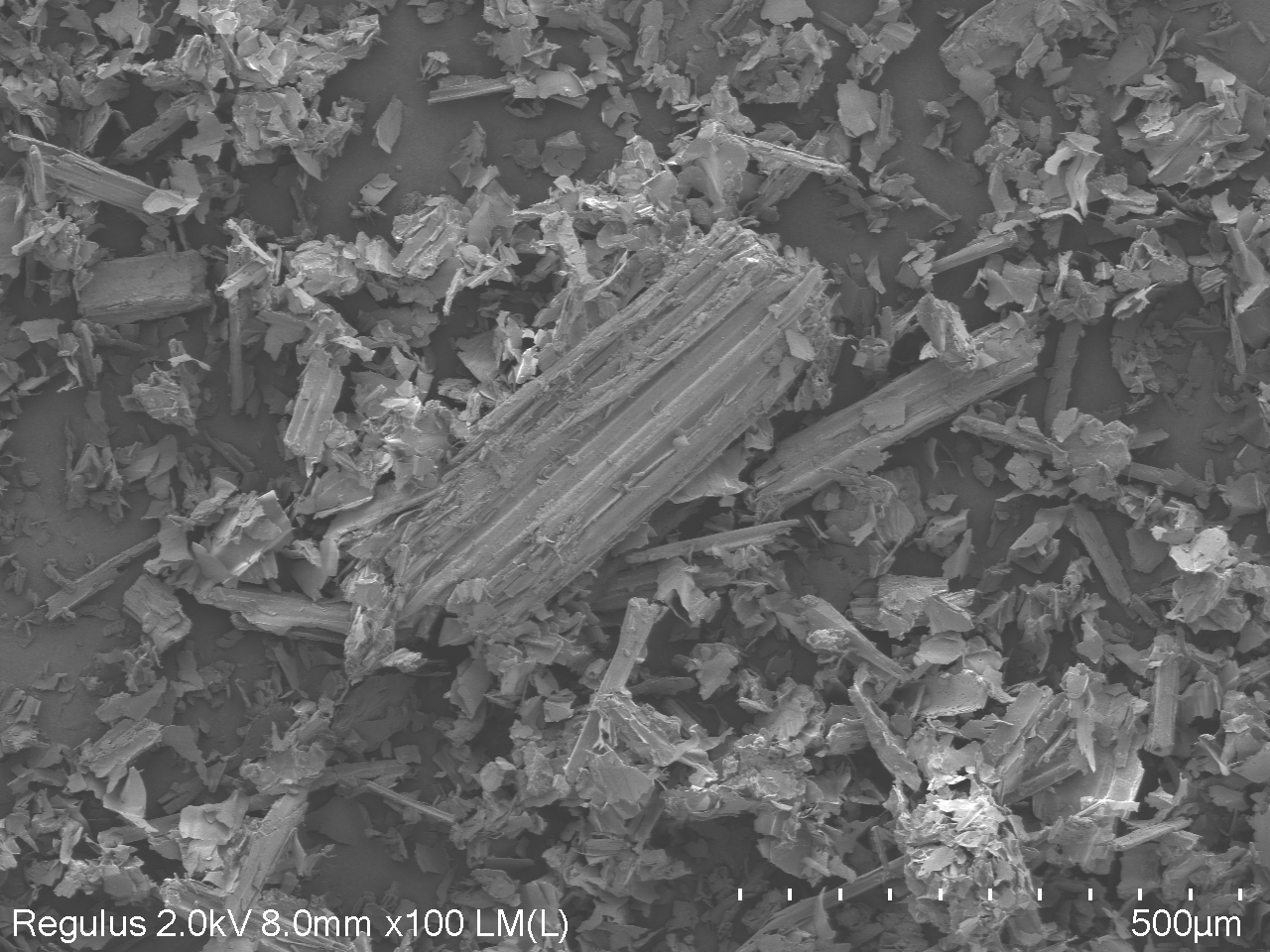

Supplement: Supplemental Information 5 [file peerj-13-20386-s005.zip › 1SEM1124/1-5.tif]

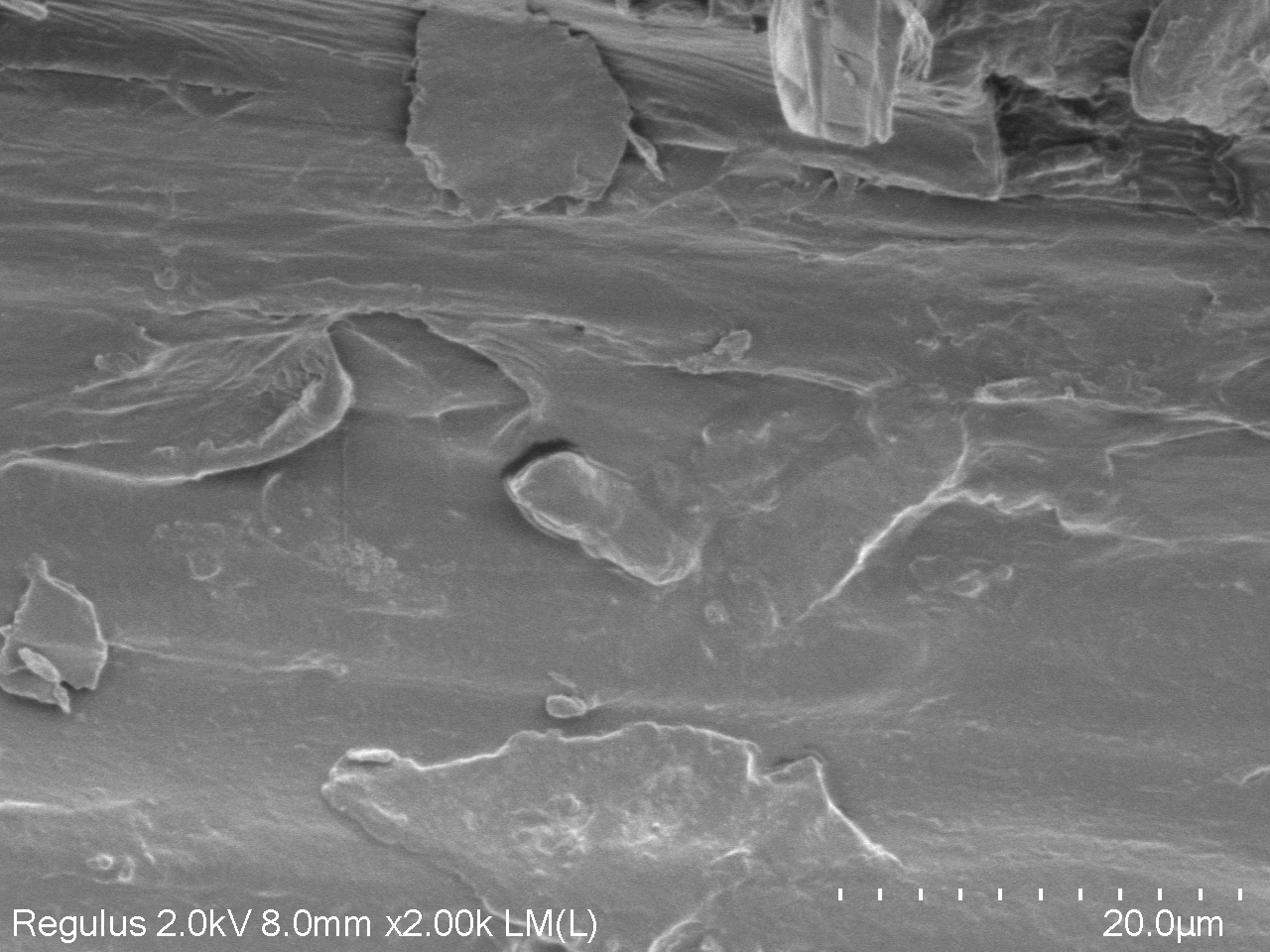

Supplement: Supplemental Information 5 [file peerj-13-20386-s005.zip › 1SEM1124/1-6.tif]

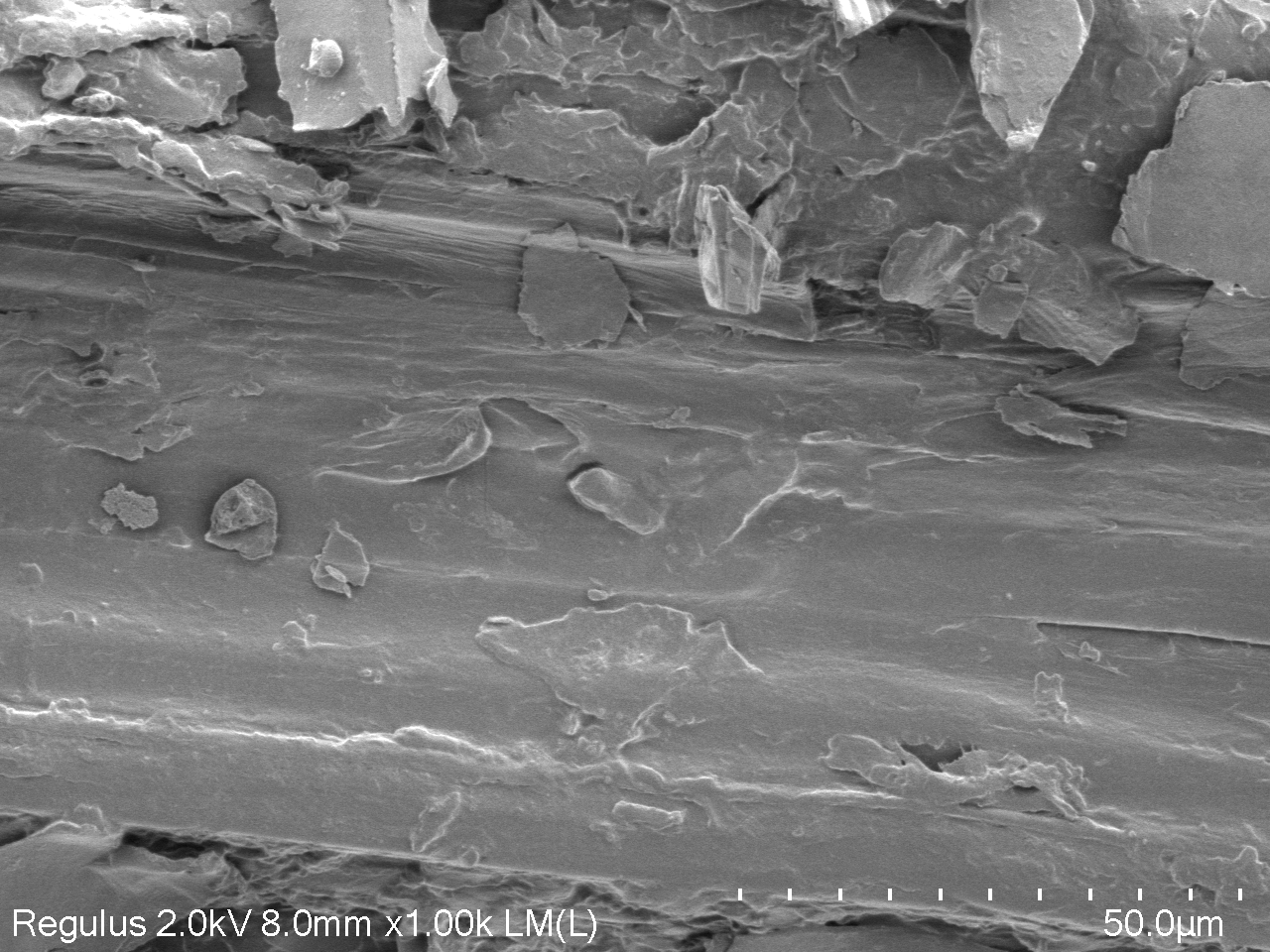

Supplement: Supplemental Information 5 [file peerj-13-20386-s005.zip › 1SEM1124/1-7.tif]

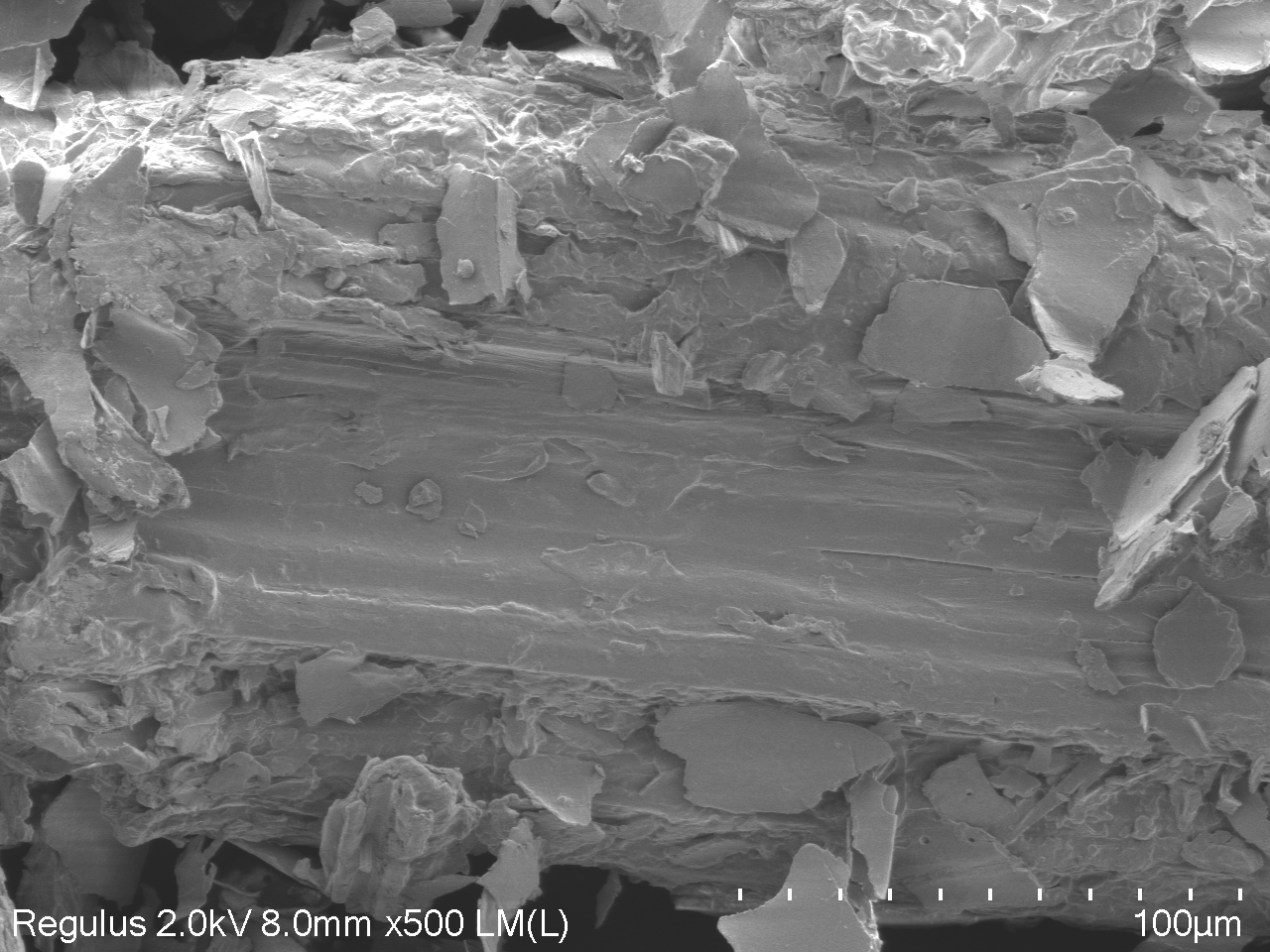

Supplement: Supplemental Information 5 [file peerj-13-20386-s005.zip › 1SEM1124/1-8.tif]

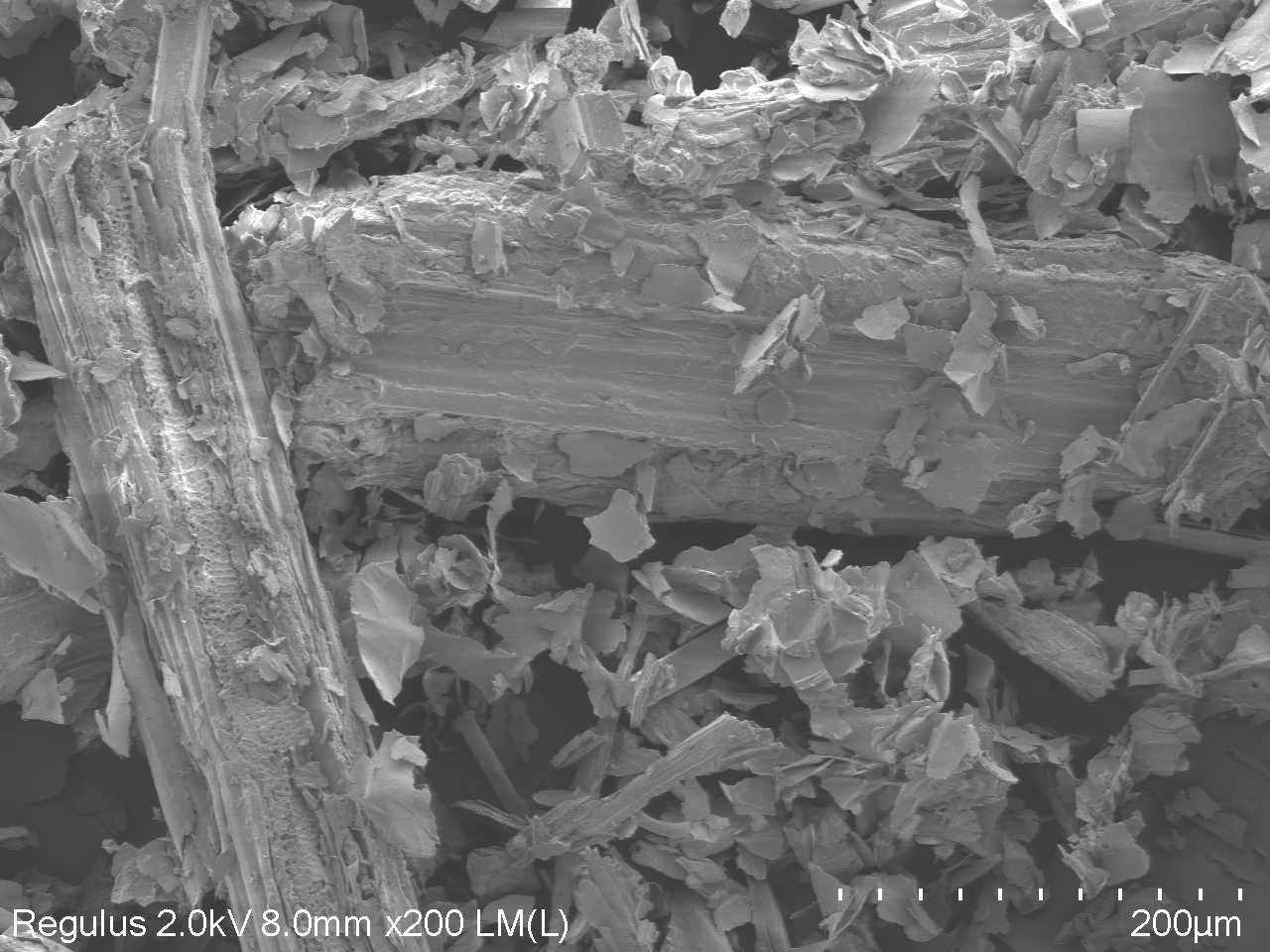

Supplement: Supplemental Information 5 [file peerj-13-20386-s005.zip › 1SEM1124/1-9.tif]

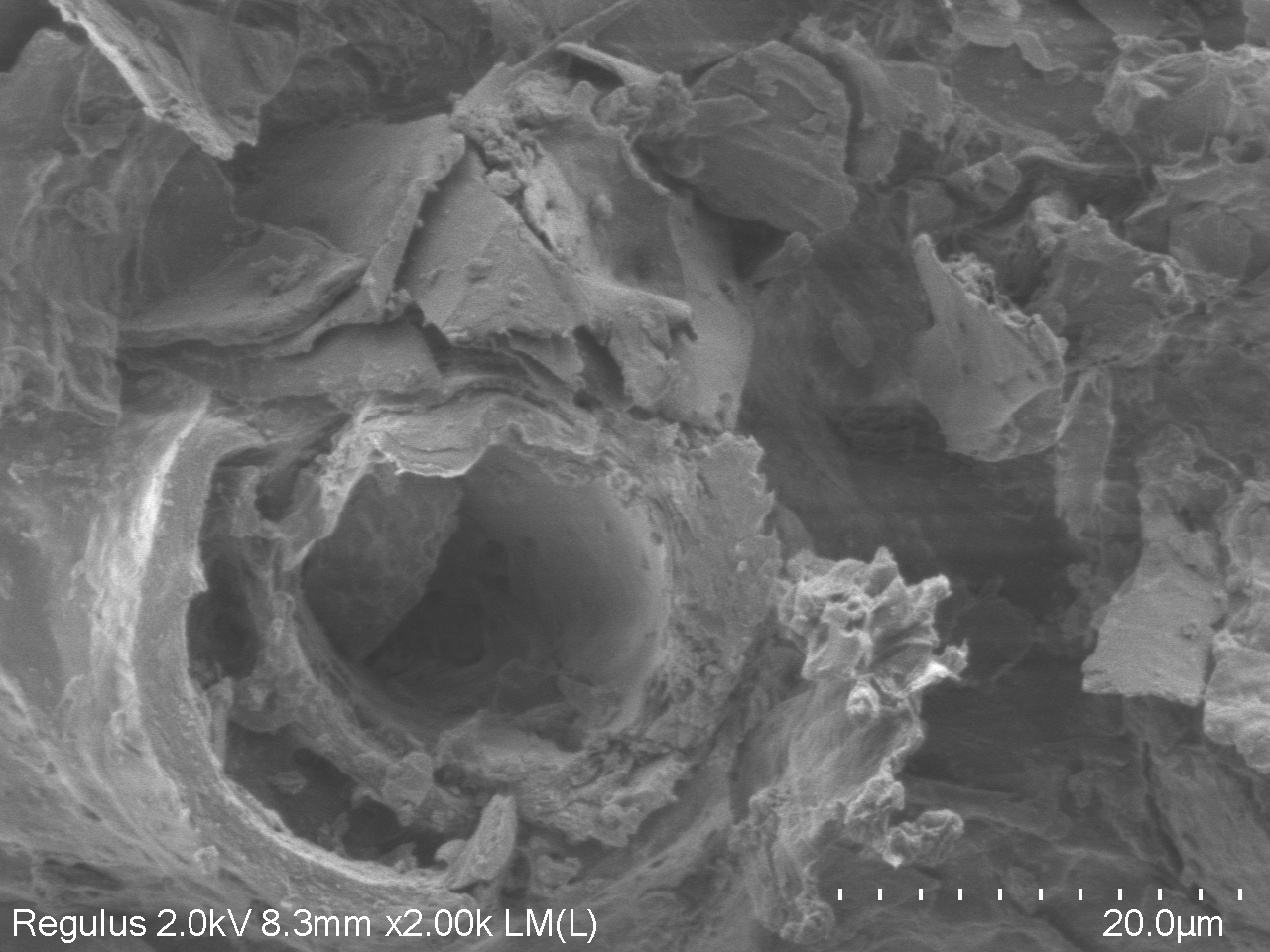

Supplement: Supplemental Information 6 [file peerj-13-20386-s006.zip › 3SEM1112/3d/2-1.tif]

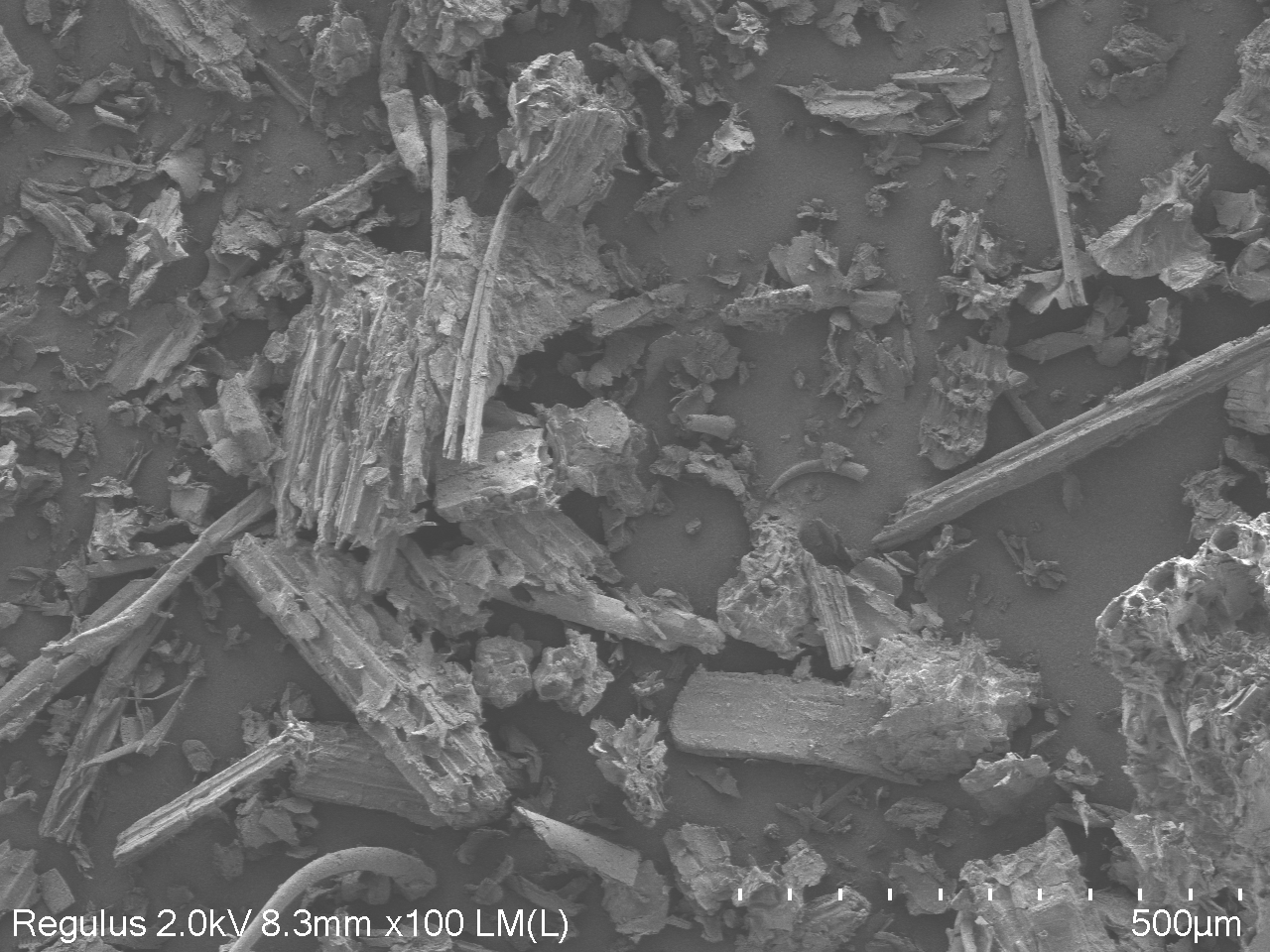

Supplement: Supplemental Information 6 [file peerj-13-20386-s006.zip › 3SEM1112/3d/2-10.tif]

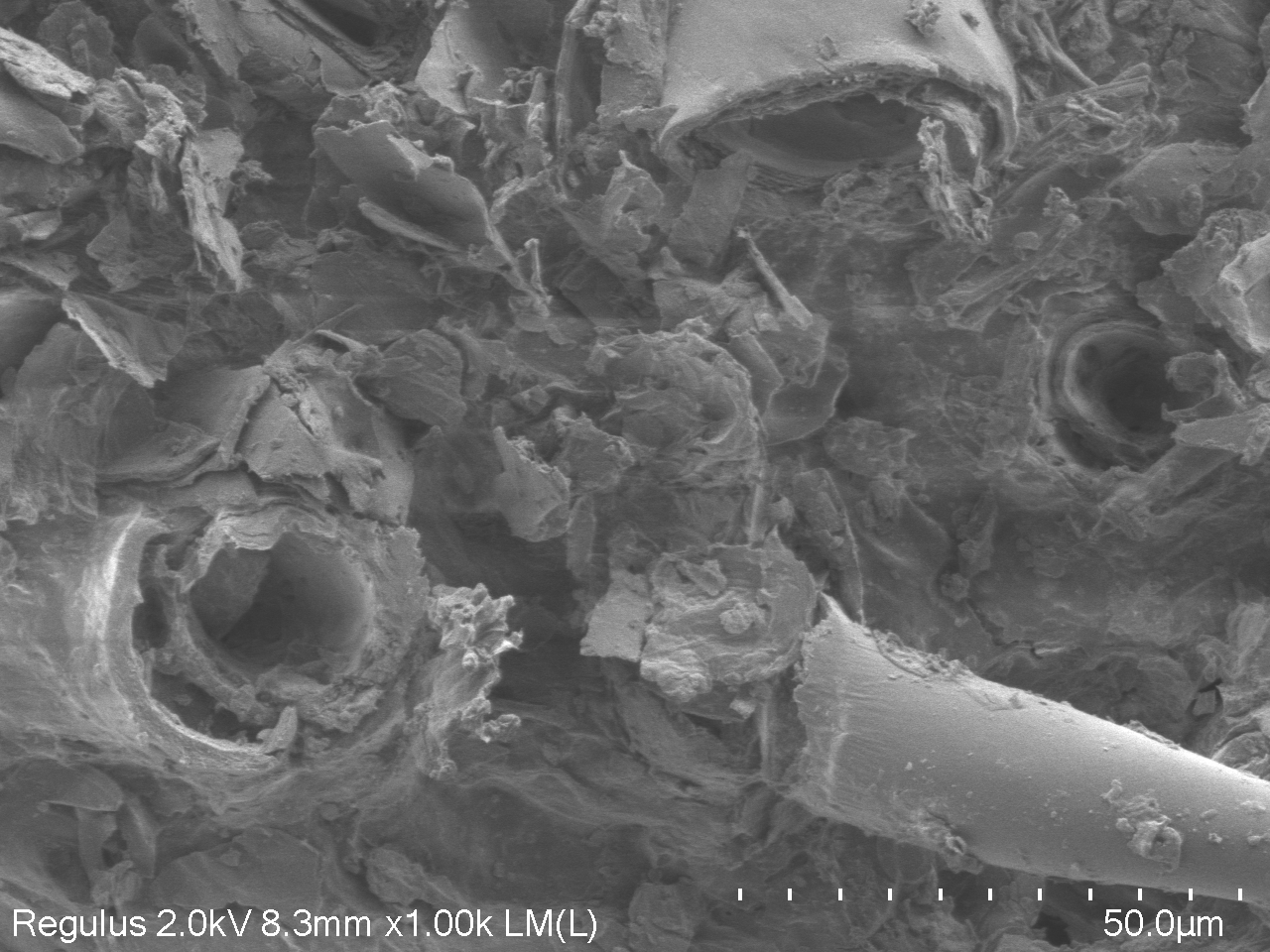

Supplement: Supplemental Information 6 [file peerj-13-20386-s006.zip › 3SEM1112/3d/2-2.tif]

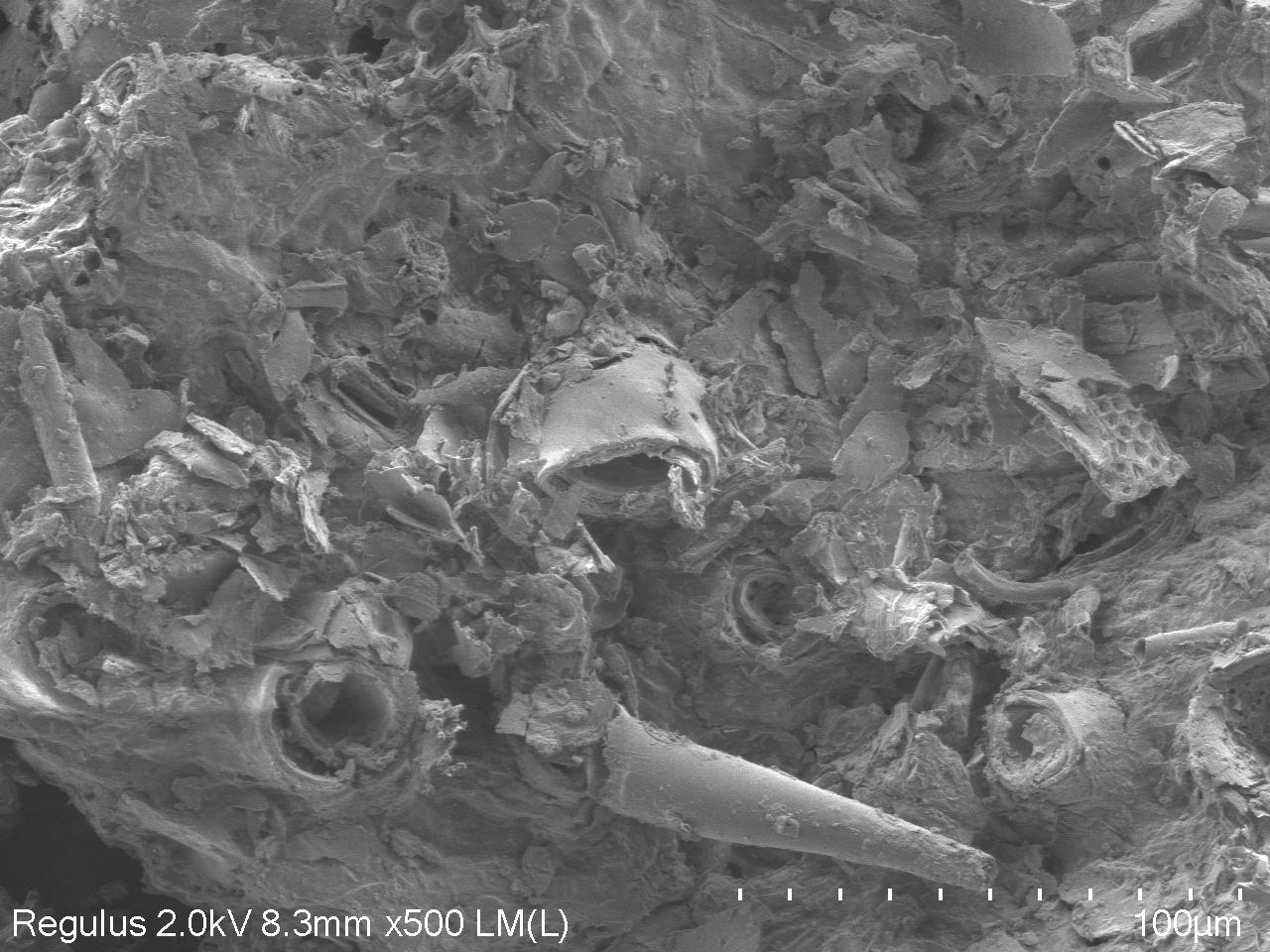

Supplement: Supplemental Information 6 [file peerj-13-20386-s006.zip › 3SEM1112/3d/2-3.tif]

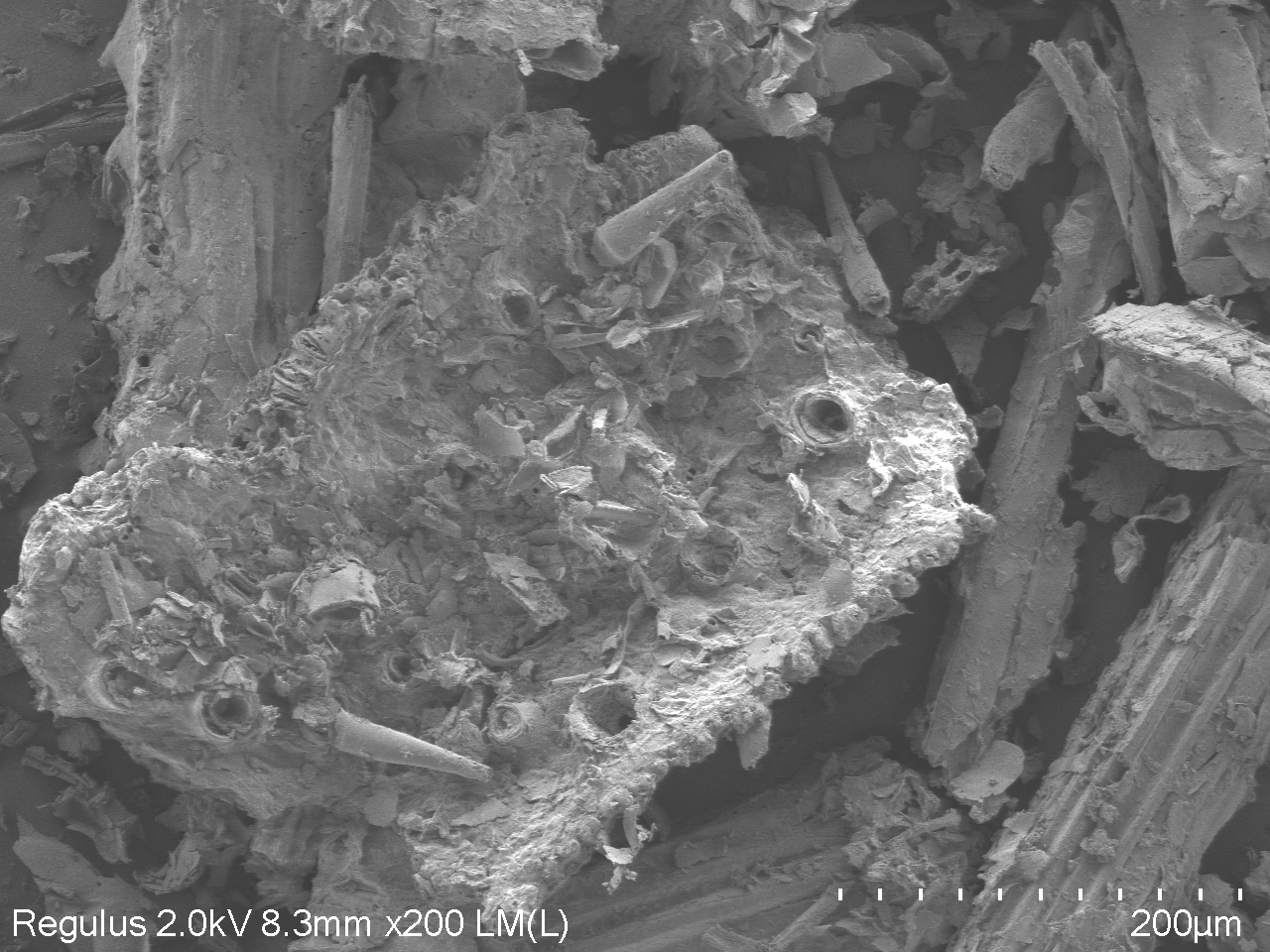

Supplement: Supplemental Information 6 [file peerj-13-20386-s006.zip › 3SEM1112/3d/2-4.tif]

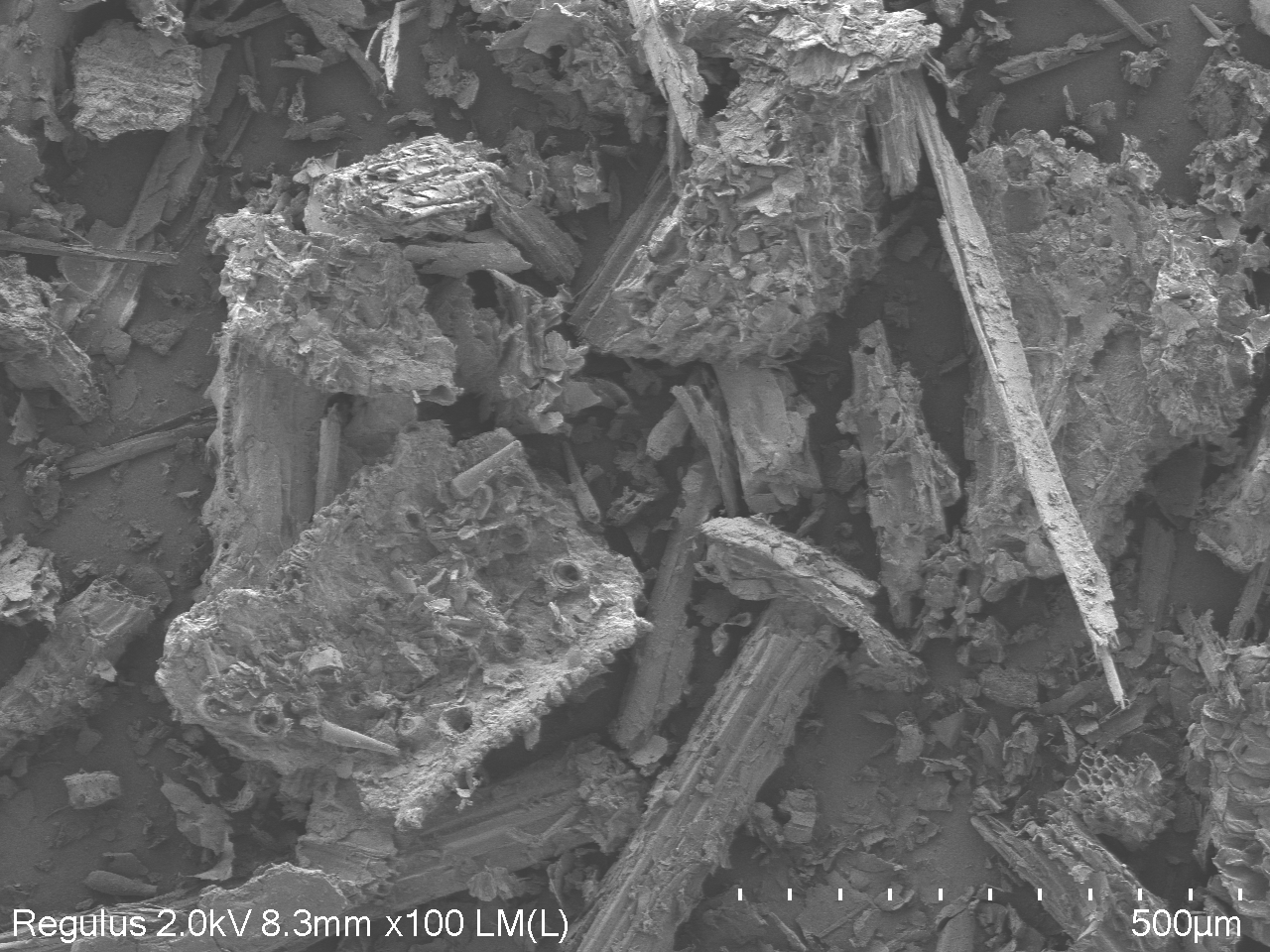

Supplement: Supplemental Information 6 [file peerj-13-20386-s006.zip › 3SEM1112/3d/2-5.tif]

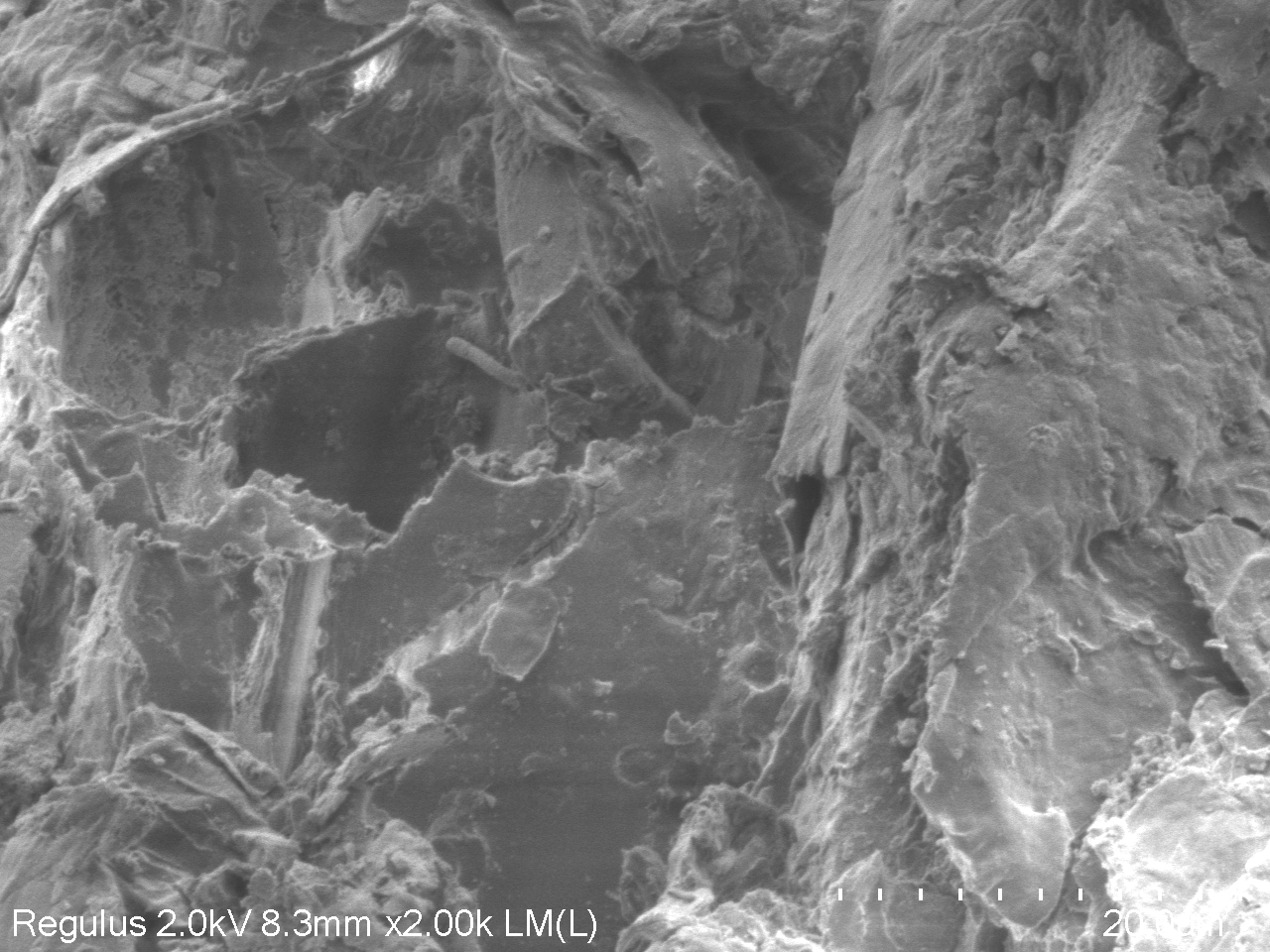

Supplement: Supplemental Information 6 [file peerj-13-20386-s006.zip › 3SEM1112/3d/2-6.tif]

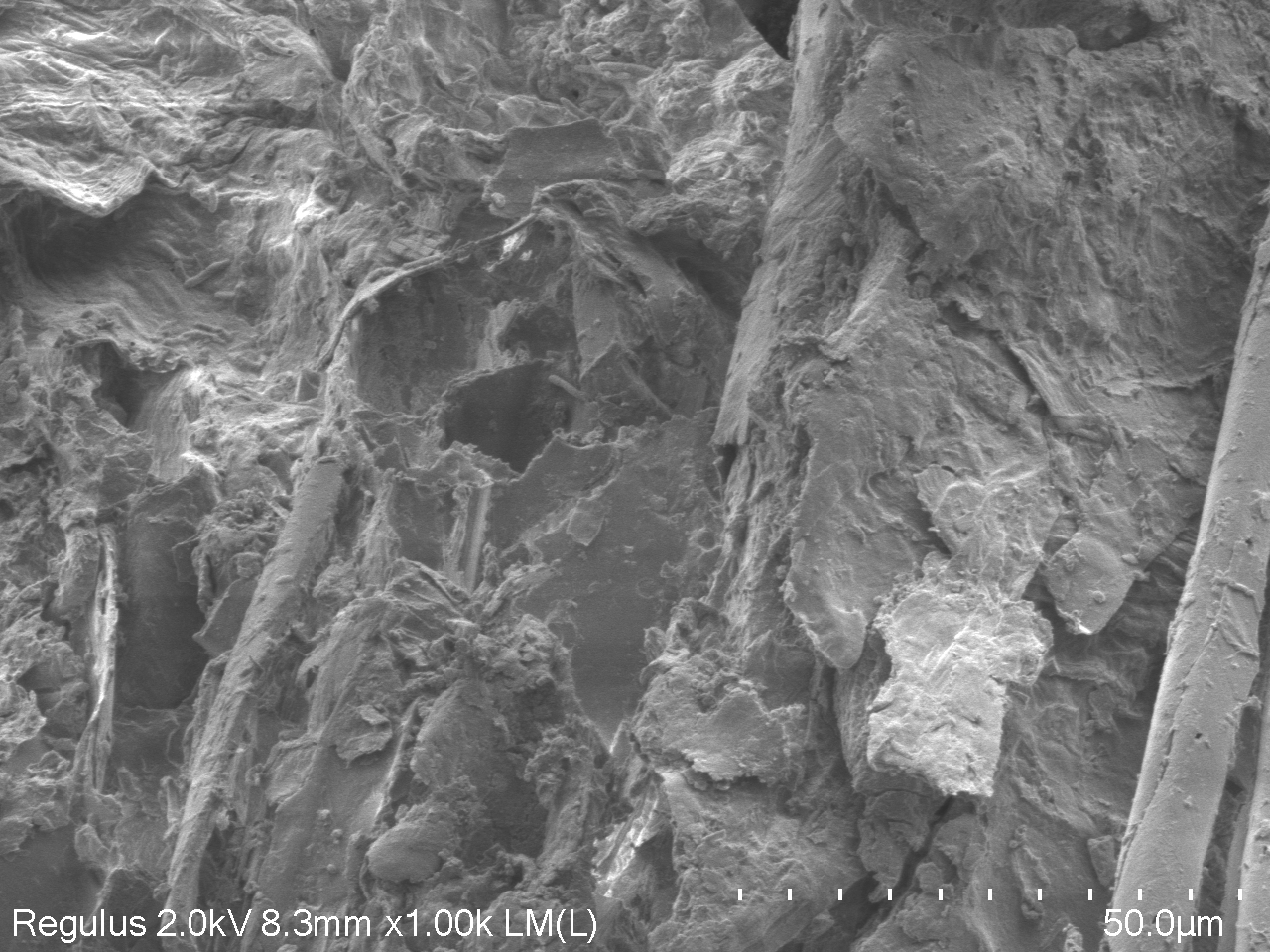

Supplement: Supplemental Information 6 [file peerj-13-20386-s006.zip › 3SEM1112/3d/2-7.tif]

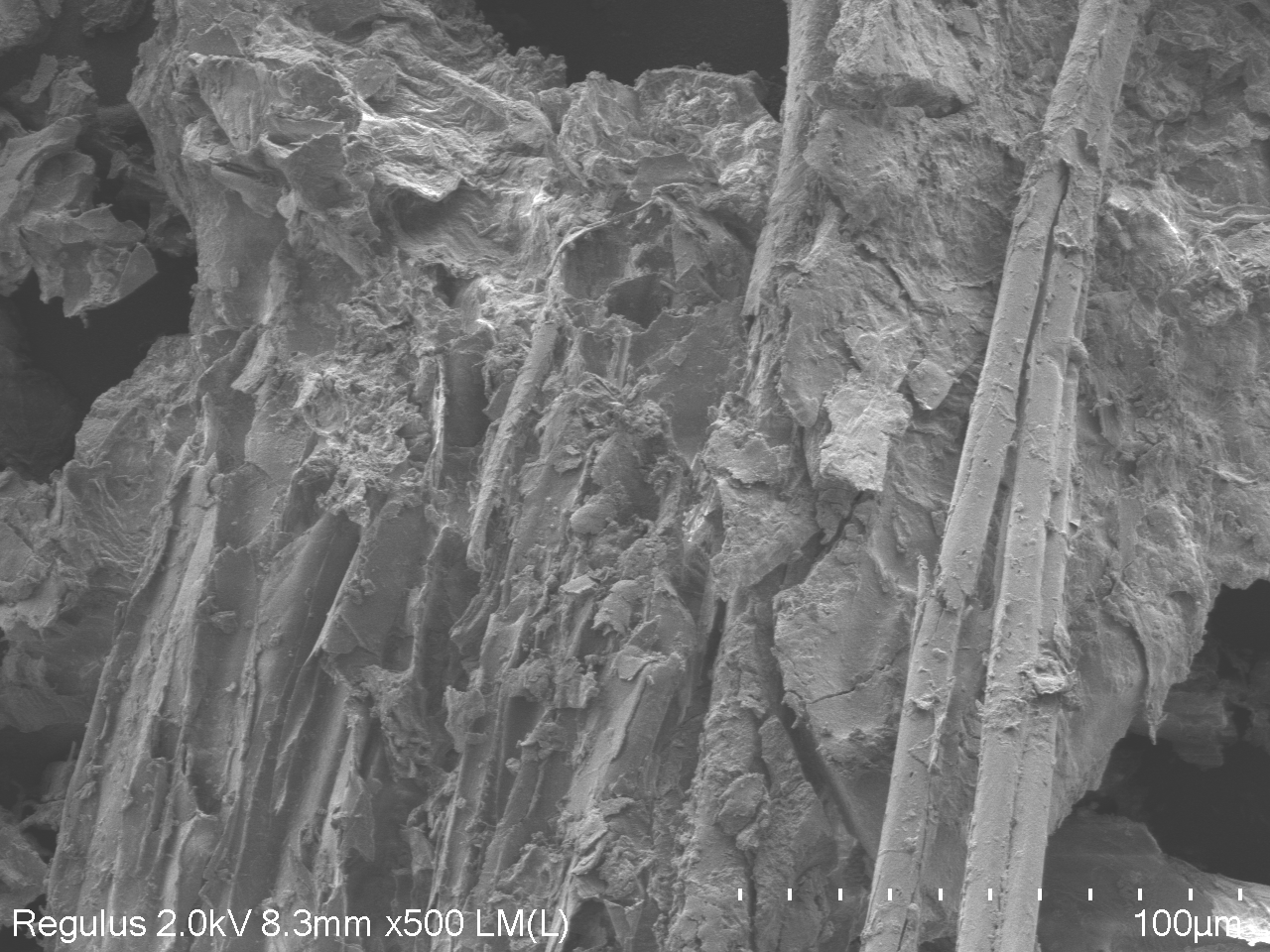

Supplement: Supplemental Information 6 [file peerj-13-20386-s006.zip › 3SEM1112/3d/2-8.tif]

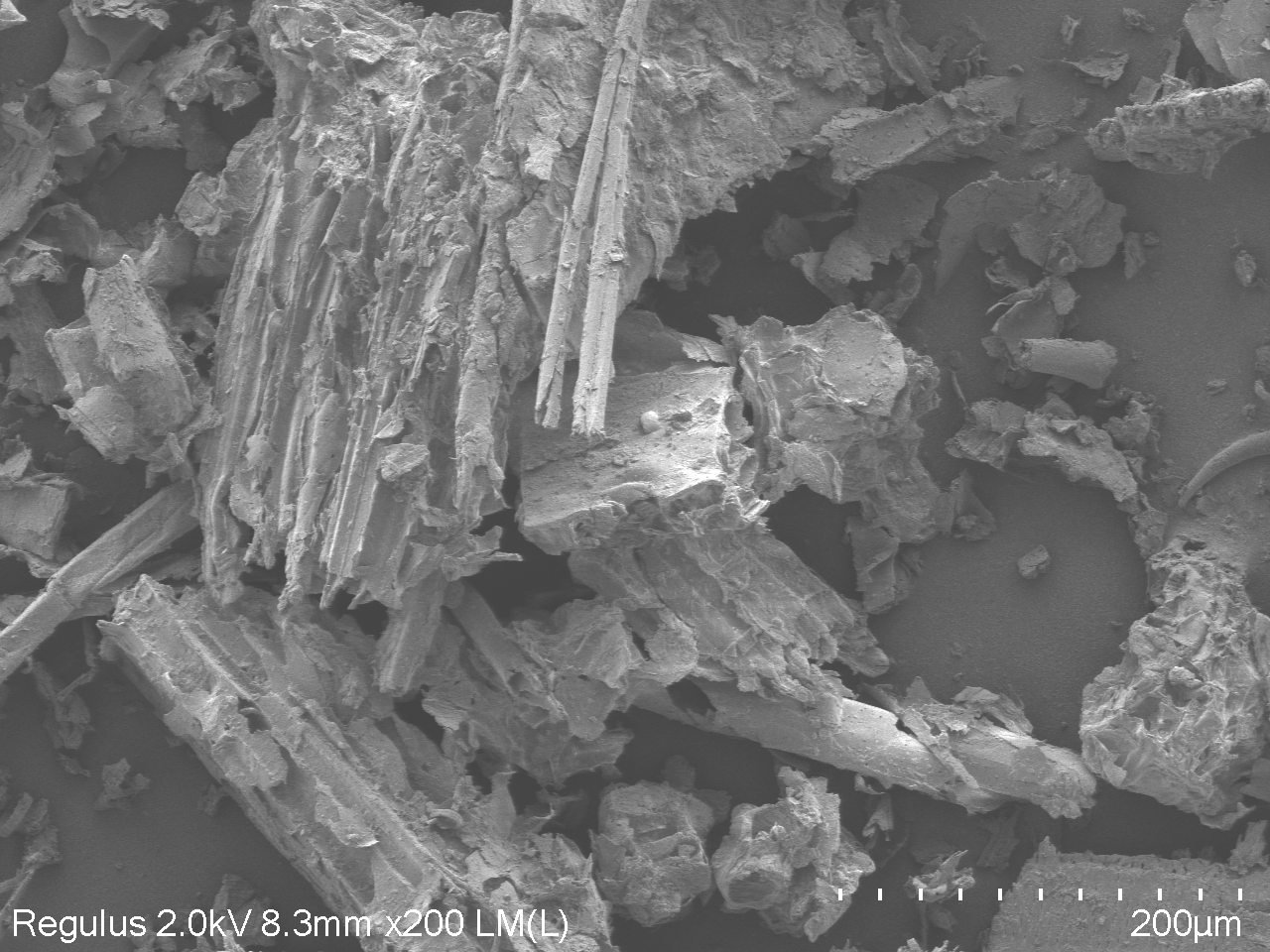

Supplement: Supplemental Information 6 [file peerj-13-20386-s006.zip › 3SEM1112/3d/2-9.tif]

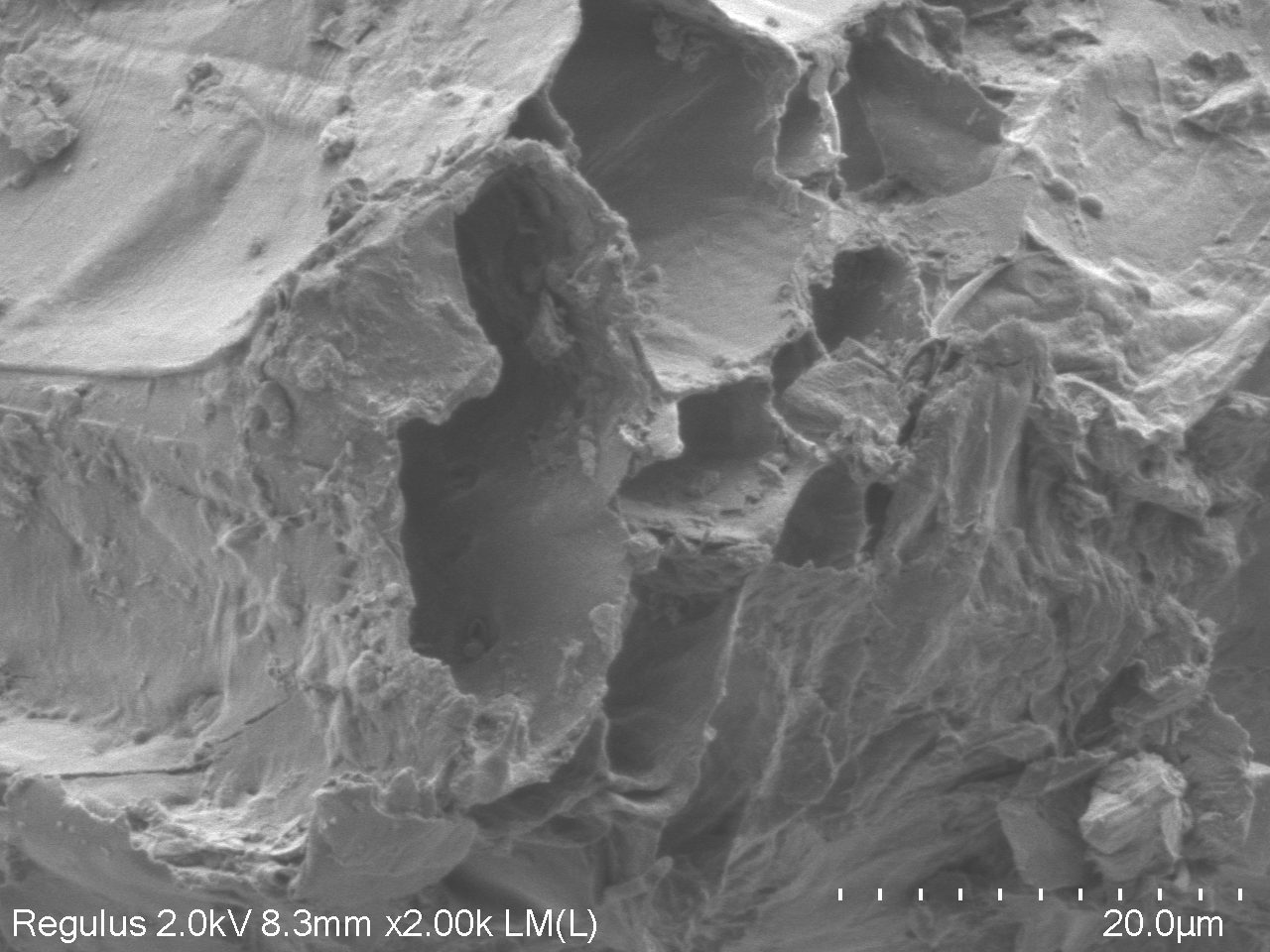

Supplement: Supplemental Information 6 [file peerj-13-20386-s006.zip › 3SEM1112/5d/3-1.tif]

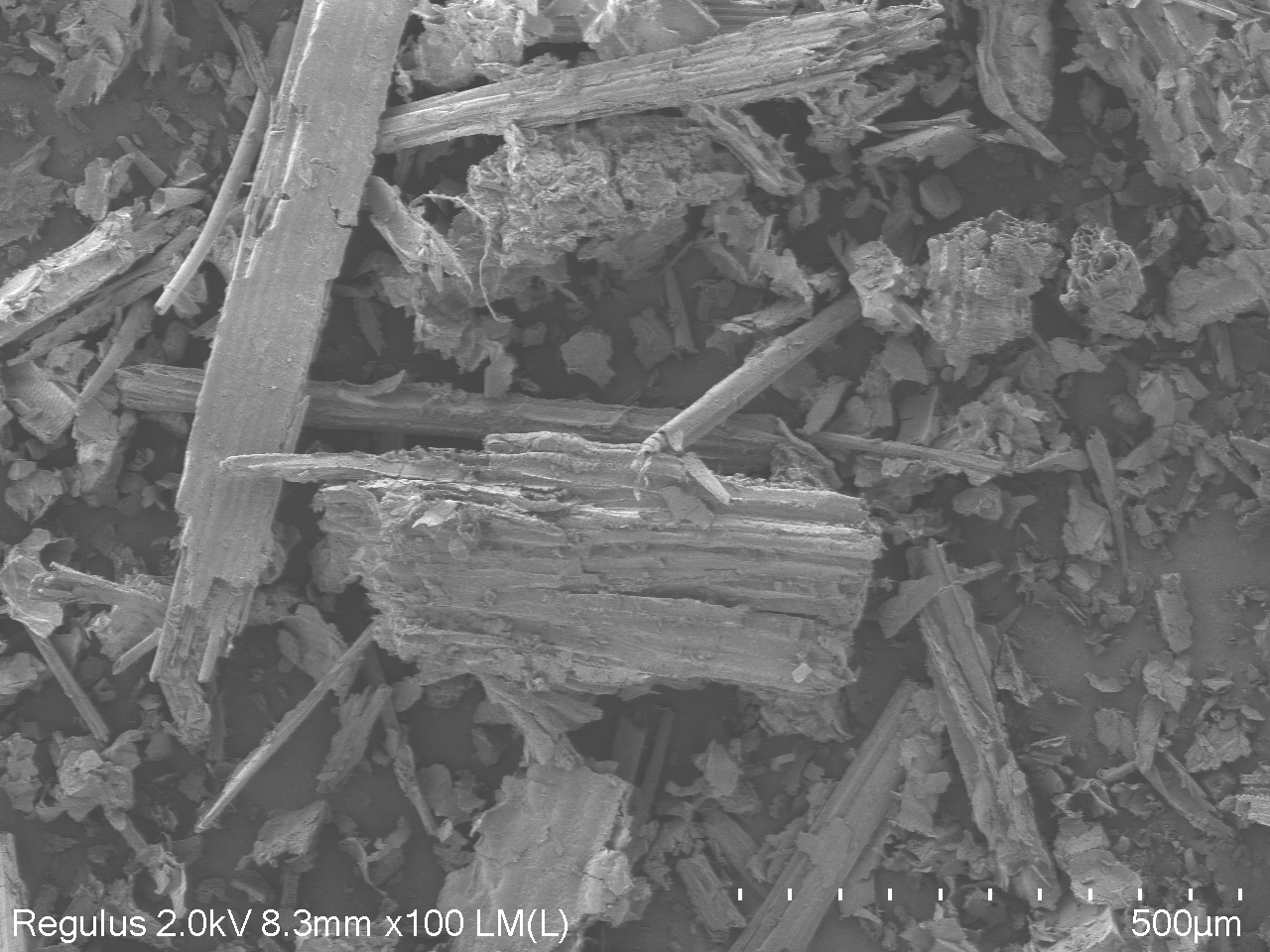

Supplement: Supplemental Information 6 [file peerj-13-20386-s006.zip › 3SEM1112/5d/3-10.tif]

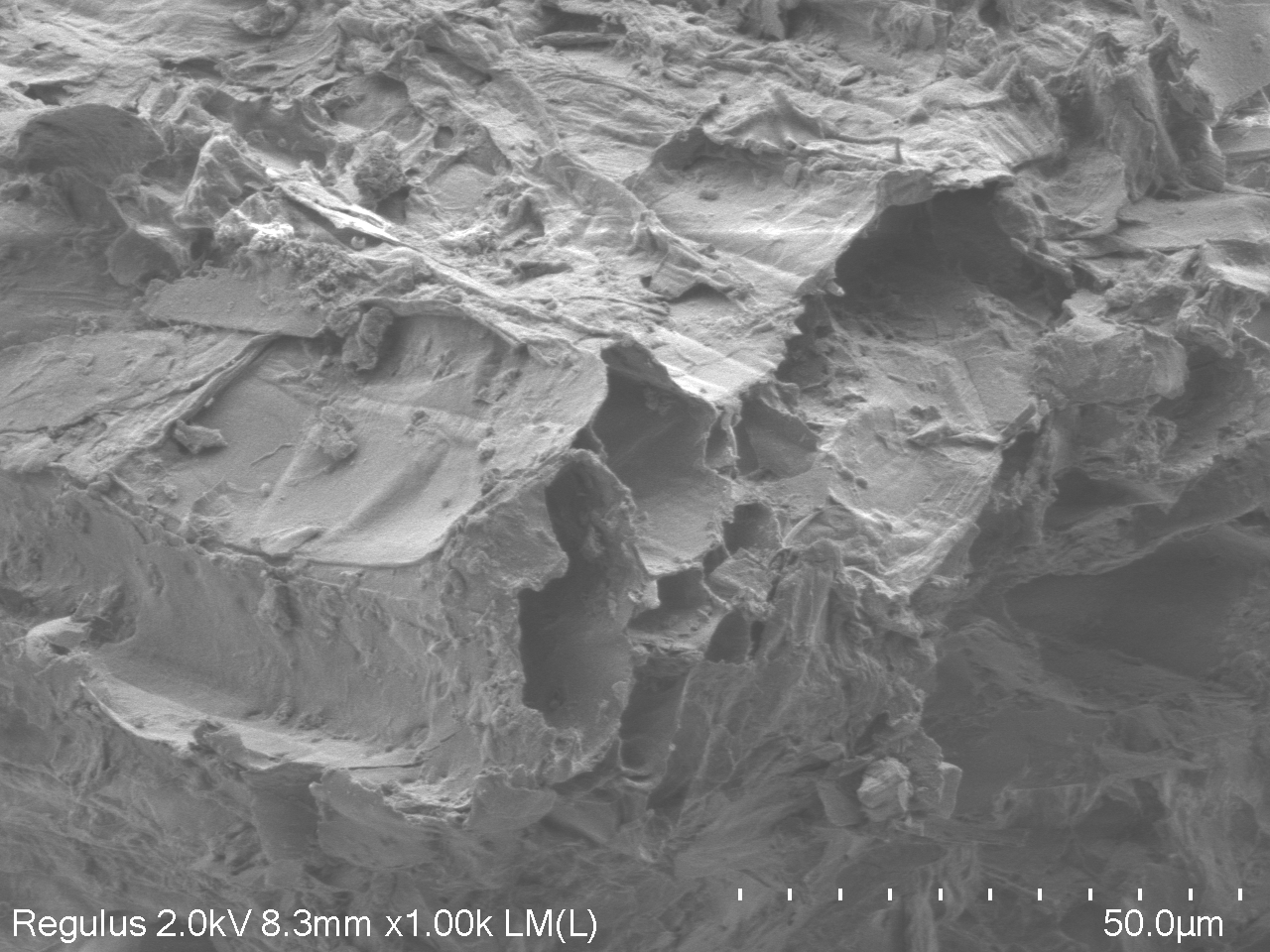

Supplement: Supplemental Information 6 [file peerj-13-20386-s006.zip › 3SEM1112/5d/3-2.tif]

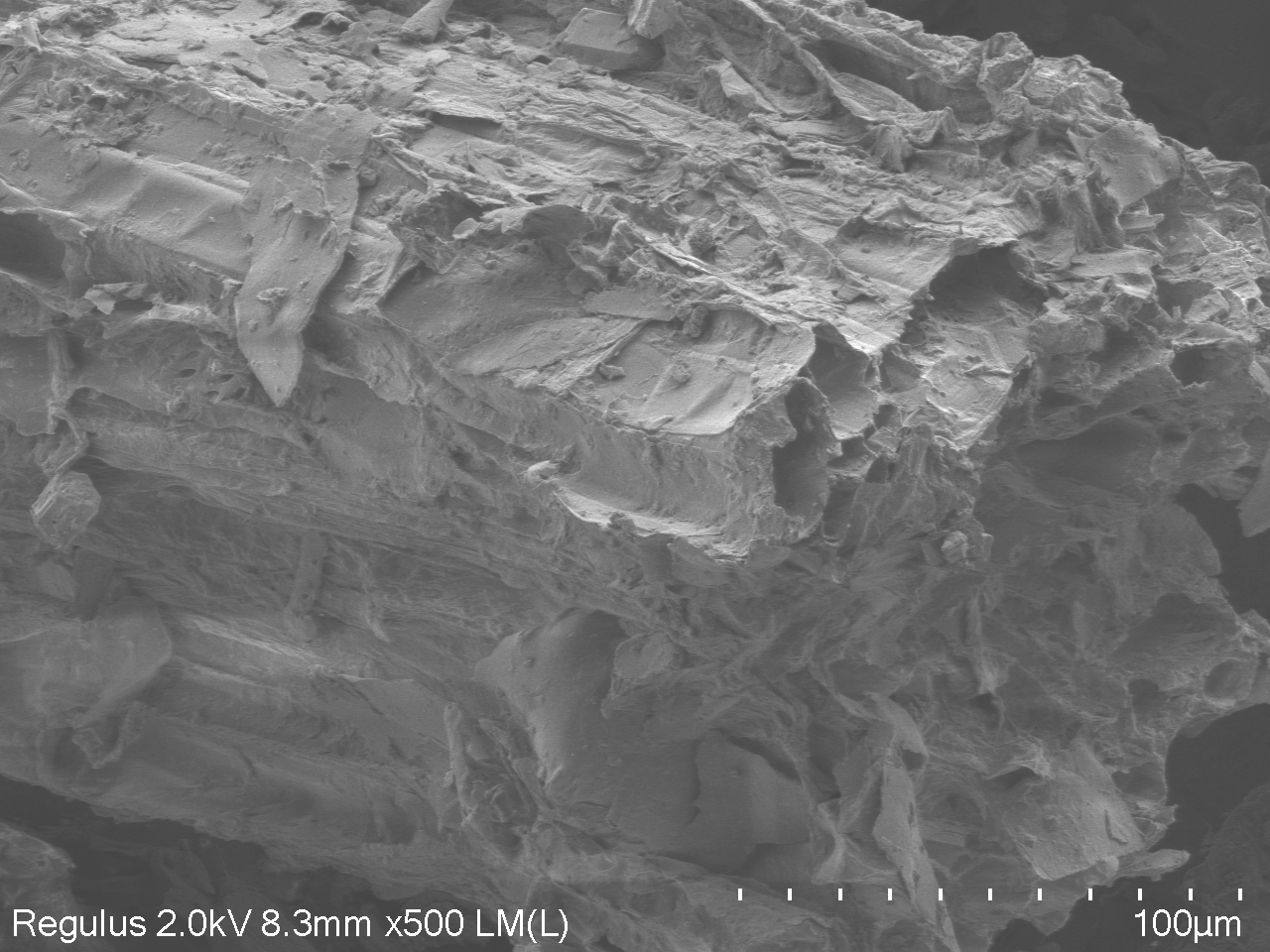

Supplement: Supplemental Information 6 [file peerj-13-20386-s006.zip › 3SEM1112/5d/3-3.tif]

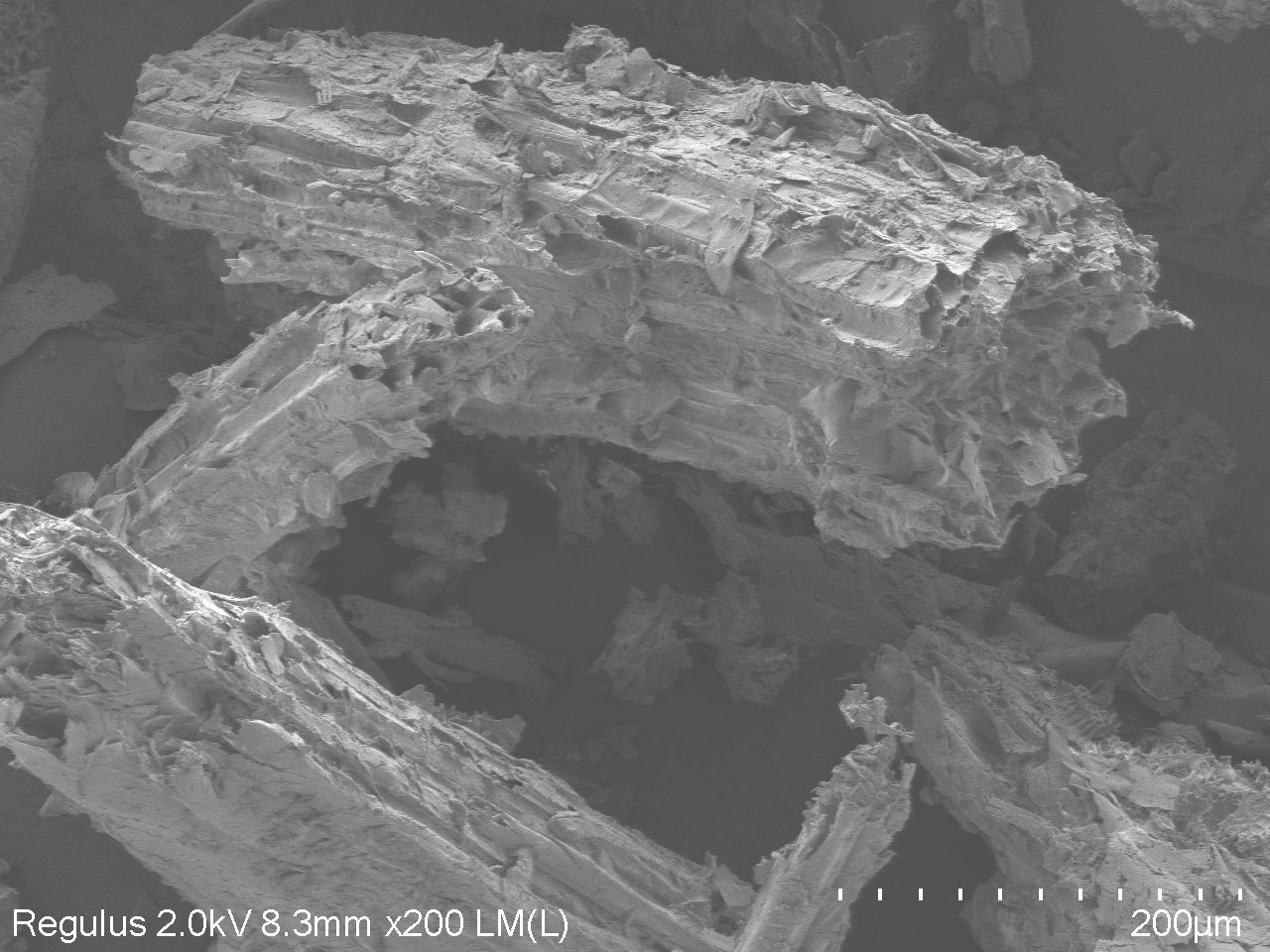

Supplement: Supplemental Information 6 [file peerj-13-20386-s006.zip › 3SEM1112/5d/3-4.tif]

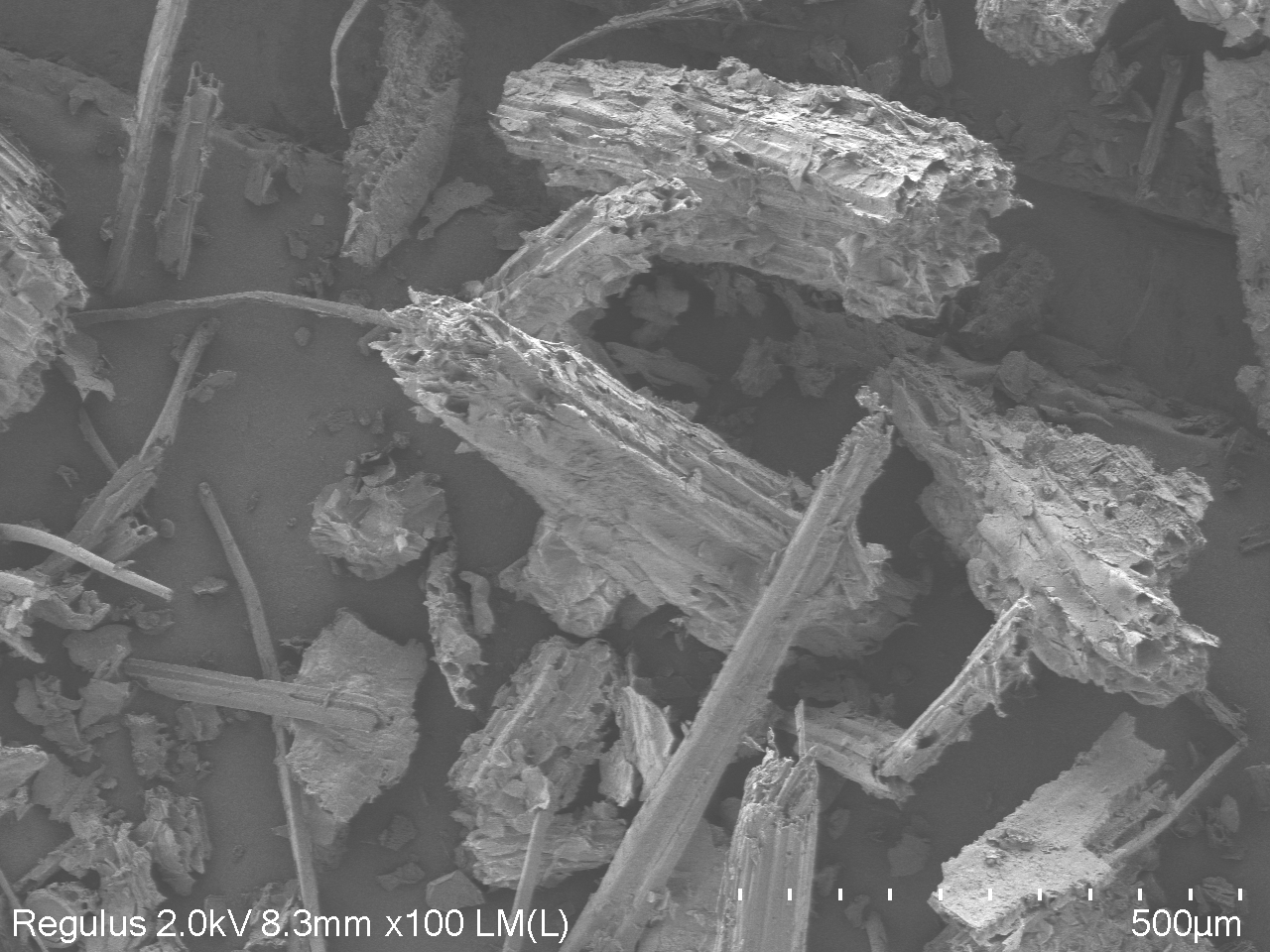

Supplement: Supplemental Information 6 [file peerj-13-20386-s006.zip › 3SEM1112/5d/3-5.tif]

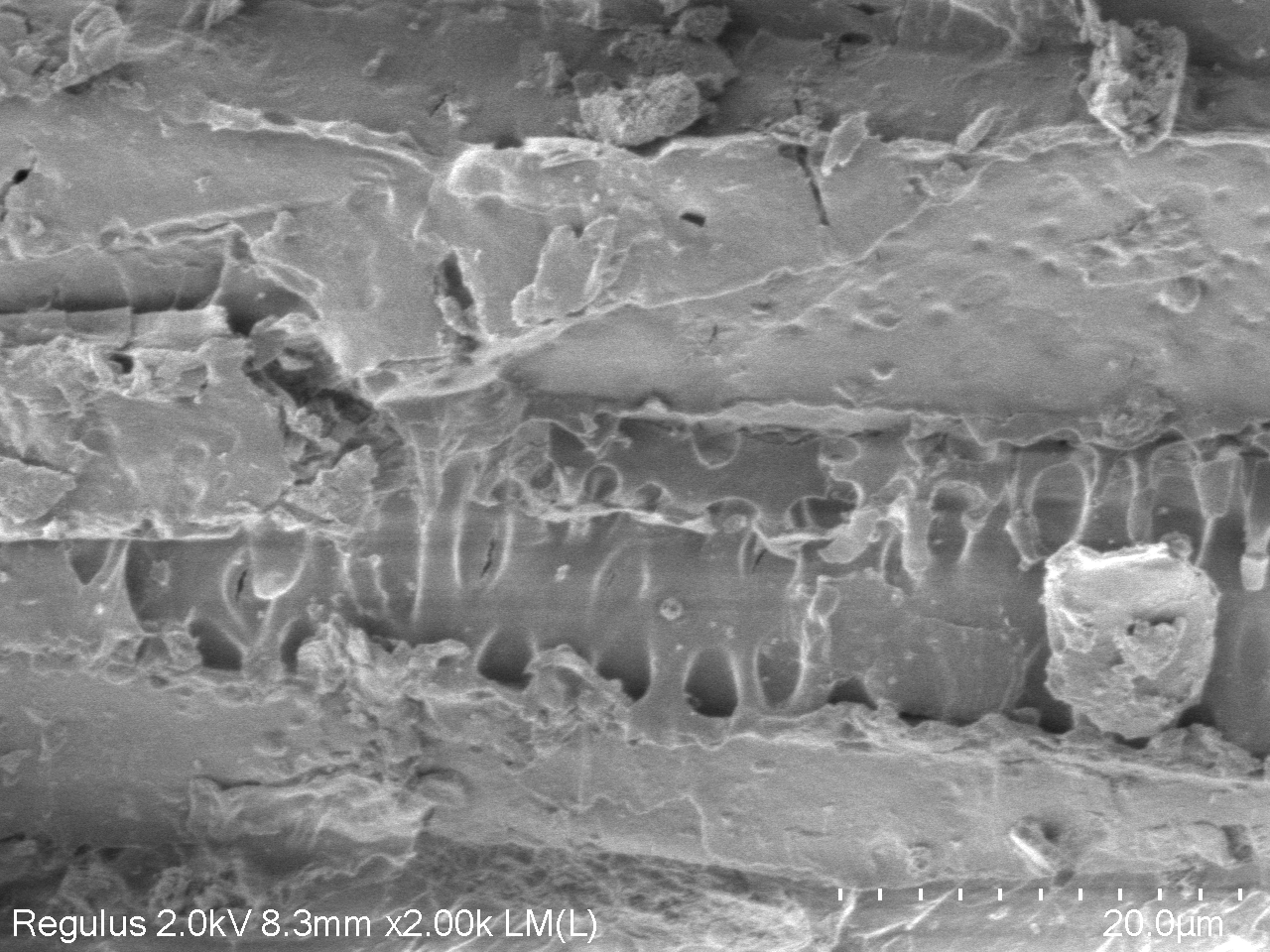

Supplement: Supplemental Information 6 [file peerj-13-20386-s006.zip › 3SEM1112/5d/3-6.tif]
