# Supplementary material for: Acclimation and degradation characteristic of the microbial system in corn straw
Source: PeerJ. 2025 Dec 16;13:e20386. doi: 10.7717/peerj.20386 (PMC12716131; doi:10.7717/peerj.20386)
Supplement: Supplemental Information 3 [file peerj-13-20386-s003.zip › Raw data 3 Structural of microbial communities/Group.KEGG.enzyme.percentage.top30.histogram.pdf]

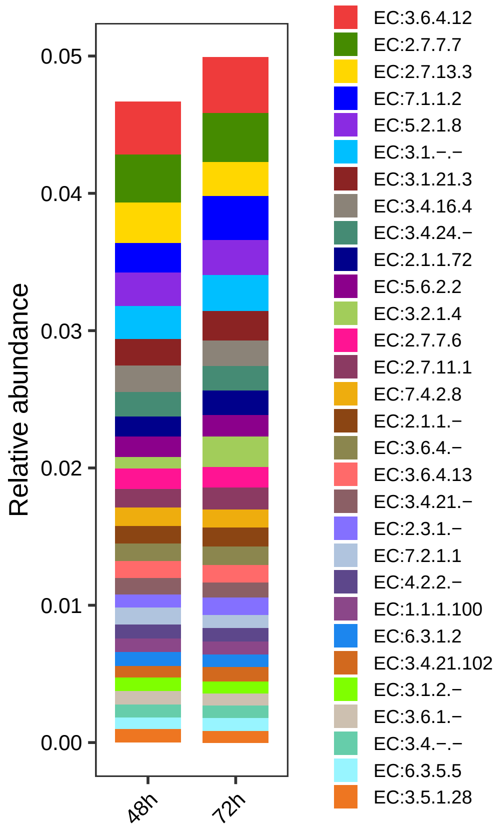

Relative abundance
0.00
0.01
0.02
0.03
0.04
0.05
48h
72h
EC:3.6.4.12
EC:2.7.7.7
EC:2.7.13.3
EC:7.1.1.2
EC:5.2.1.8
EC:3.1.−.−
EC:3.1.21.3
EC:3.4.16.4
EC:3.4.24.−
EC:2.1.1.72
EC:5.6.2.2
EC:3.2.1.4
EC:2.7.7.6
EC:2.7.11.1
EC:7.4.2.8
EC:2.1.1.−
EC:3.6.4.−
EC:3.6.4.13
EC:3.4.21.−
EC:2.3.1.−
EC:7.2.1.1
EC:4.2.2.−
EC:1.1.1.100
EC:6.3.1.2
EC:3.4.21.102
EC:3.1.2.−
EC:3.6.1.−
EC:3.4.−.−
EC:6.3.5.5
EC:3.5.1.28
