# Supplementary material for: Acclimation and degradation characteristic of the microbial system in corn straw
Source: PeerJ. 2025 Dec 16;13:e20386. doi: 10.7717/peerj.20386 (PMC12716131; doi:10.7717/peerj.20386)
Supplement: Supplemental Information 3 [file peerj-13-20386-s003.zip › Raw data 3 Structural of microbial communities/Group.KEGG.pathway_hierarchy2.percentage.top10.pie.all.pdf]

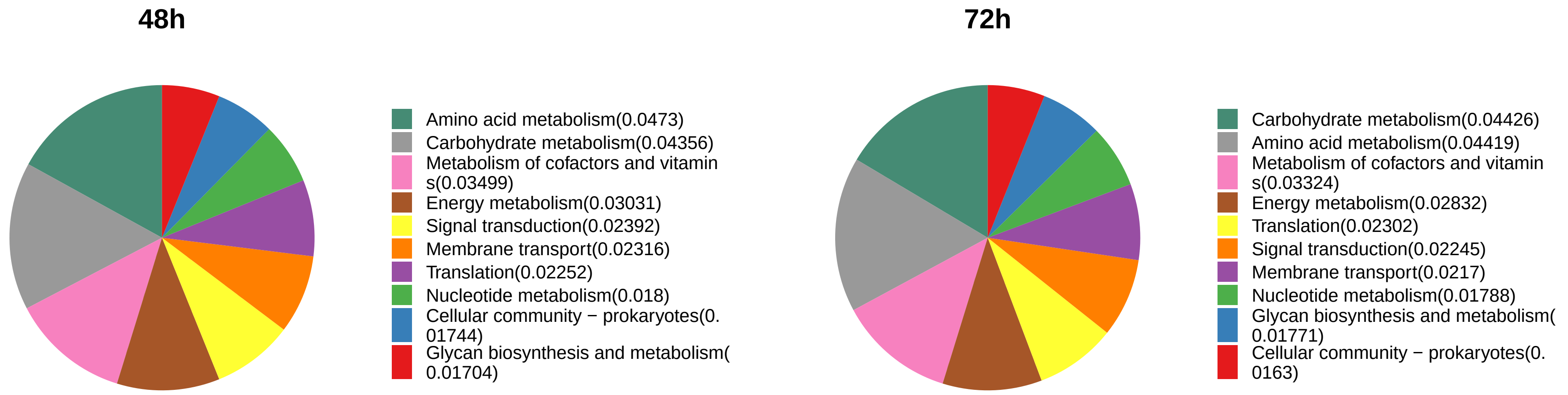
48h
Amino acid metabolism(0.0473)
Carbohydrate metabolism(0.04356)
Metabolism of cofactors and vitamin s(0.03499)
Energy metabolism(0.03031)
Signal transduction(0.02392)
Membrane transport(0.02316)
Translation(0.02252)
Nucleotide metabolism(0.018)
Cellular community − prokaryotes(0. 01744)
Glycan biosynthesis and metabolism(0.01704)
72h
Carbohydrate metabolism(0.04426)
Amino acid metabolism(0.04419)
Metabolism of cofactors and vitamin s(0.03324)
Energy metabolism(0.02832)
Translation(0.02302)
Signal transduction(0.02245)
Membrane transport(0.0217)
Nucleotide metabolism(0.01788)
Glycan biosynthesis and metabolism(0.01771)
Cellular community − prokaryotes(0. 0163)
