# Supplementary material for: Acclimation and degradation characteristic of the microbial system in corn straw
Source: PeerJ. 2025 Dec 16;13:e20386. doi: 10.7717/peerj.20386 (PMC12716131; doi:10.7717/peerj.20386)
Supplement: Supplemental Information 3 [file peerj-13-20386-s003.zip › Raw data 3 Structural of microbial communities/KEGG.pathway top10 48h.pdf]

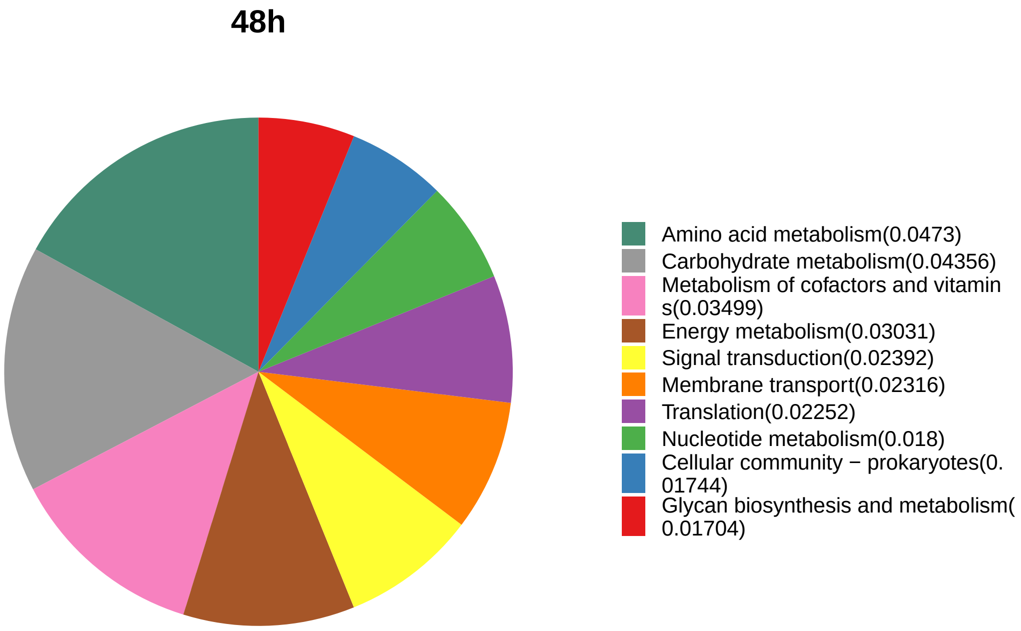
48h
Amino acid metabolism(0.0473)
Carbohydrate metabolism(0.04356)
Metabolism of cofactors and vitamins(0.03499)
Energy metabolism(0.03031)
Signal transduction(0.02392)
Membrane transport(0.02316)
Translation(0.02252)
Nucleotide metabolism(0.018)
Cellular community − prokaryotes(0.01744)
Glycan biosynthesis and metabolism(0.01704)
