# Supplementary material for: Acclimation and degradation characteristic of the microbial system in corn straw
Source: PeerJ. 2025 Dec 16;13:e20386. doi: 10.7717/peerj.20386 (PMC12716131; doi:10.7717/peerj.20386)
Supplement: Supplemental Information 3 [file peerj-13-20386-s003.zip › Raw data 3 Structural of microbial communities/KEGG.pathway72h.pdf]

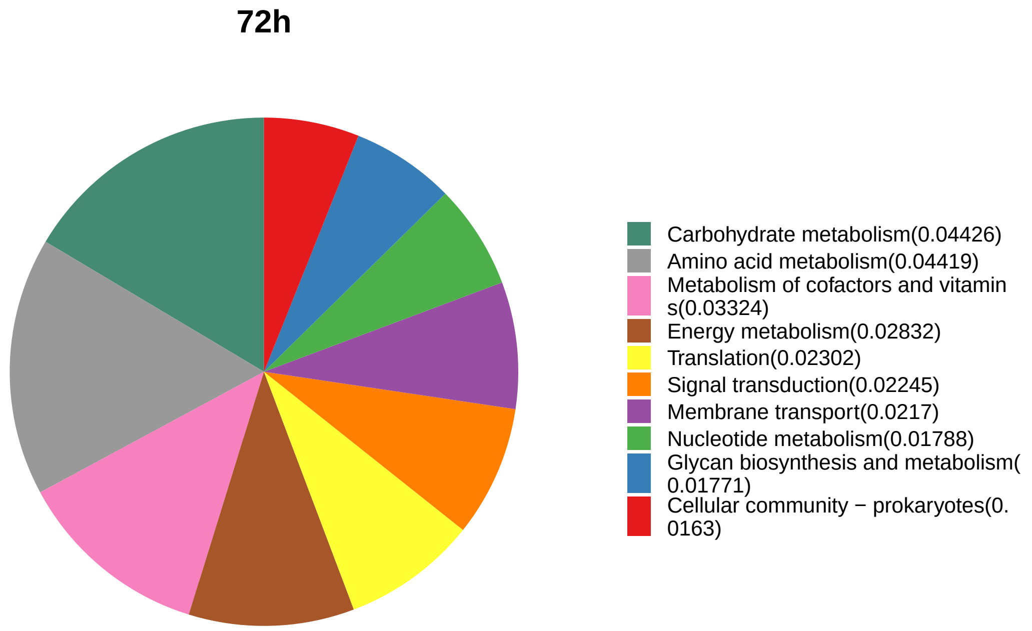
72h
Carbohydrate metabolism(0.04426)
Amino acid metabolism(0.04419)
Metabolism of cofactors and vitamins(0.03324)
Energy metabolism(0.02832)
Translation(0.02302)
Signal transduction(0.02245)
Membrane transport(0.0217)
Nucleotide metabolism(0.01788)
Glycan biosynthesis and metabolism(0.01771)
Cellular community − prokaryotes(0.0163)
