# Supplementary figures and images for: Acclimation and degradation characteristic of the microbial system in corn straw (part 2 of 2)
Source: PeerJ. 2025 Dec 16;13:e20386. doi: 10.7717/peerj.20386 (PMC12716131; doi:10.7717/peerj.20386)

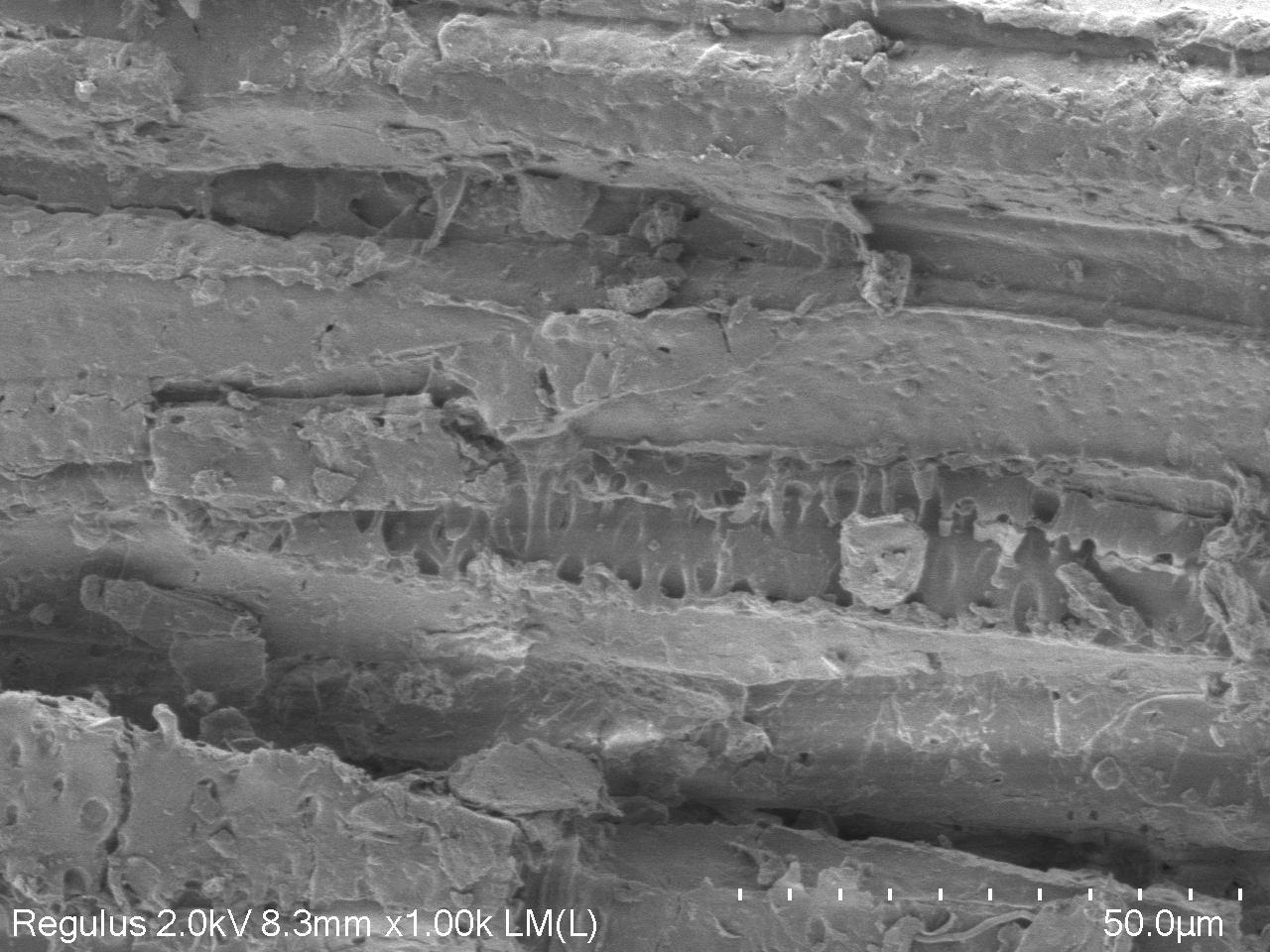

Supplement: Supplemental Information 6 [file peerj-13-20386-s006.zip › 3SEM1112/5d/3-7.tif]

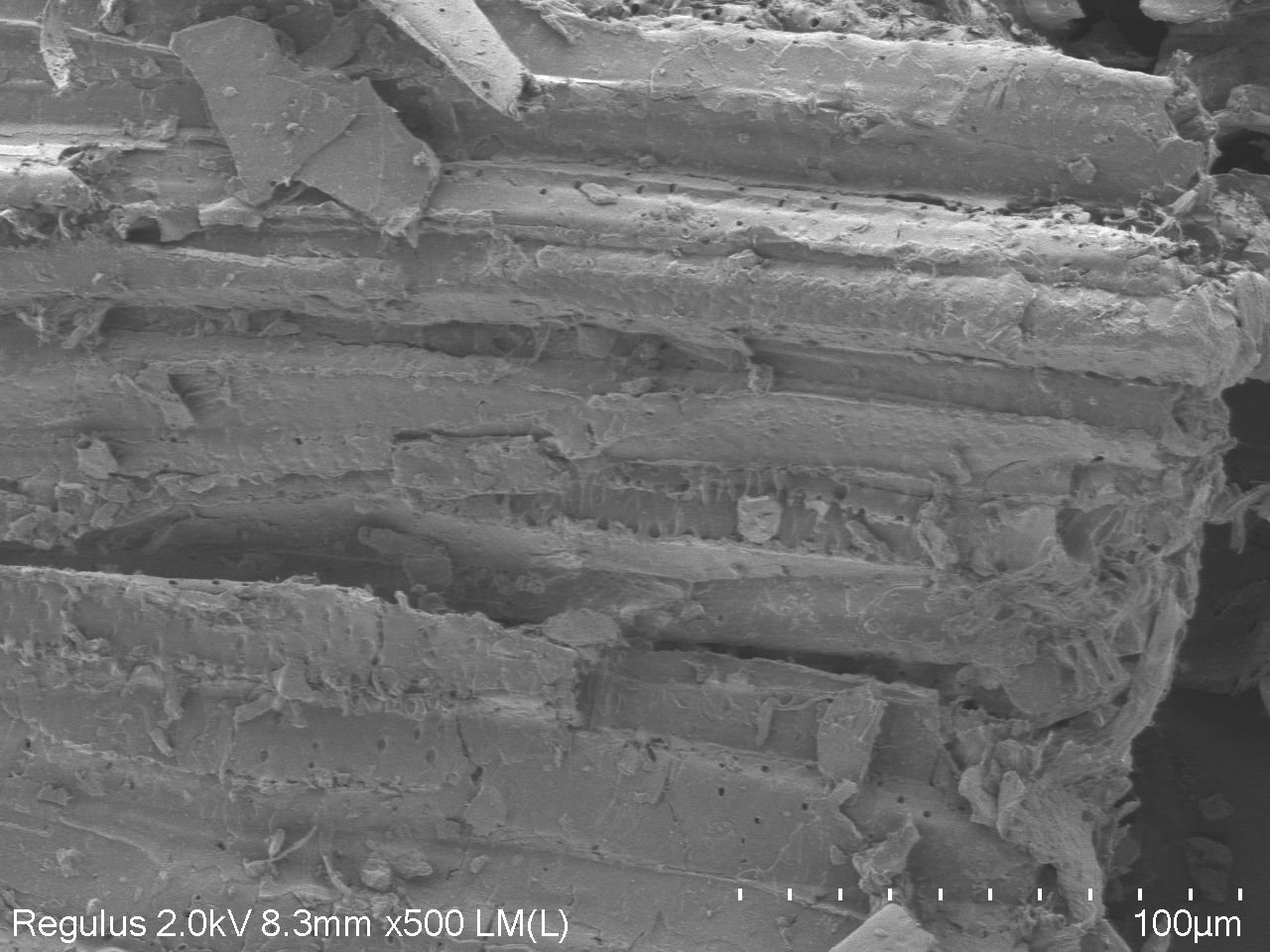

Supplement: Supplemental Information 6 [file peerj-13-20386-s006.zip › 3SEM1112/5d/3-8.tif]

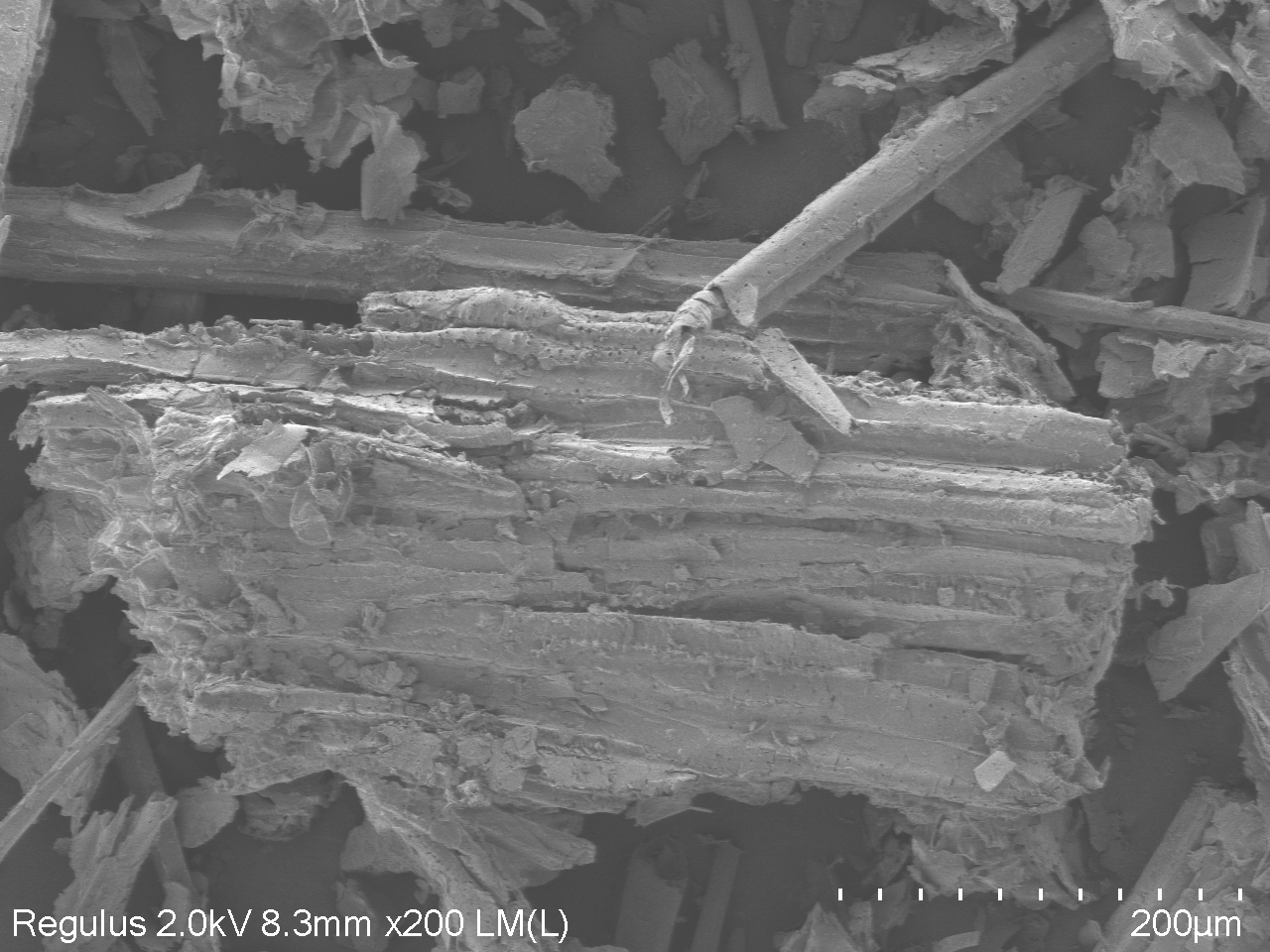

Supplement: Supplemental Information 6 [file peerj-13-20386-s006.zip › 3SEM1112/5d/3-9.tif]

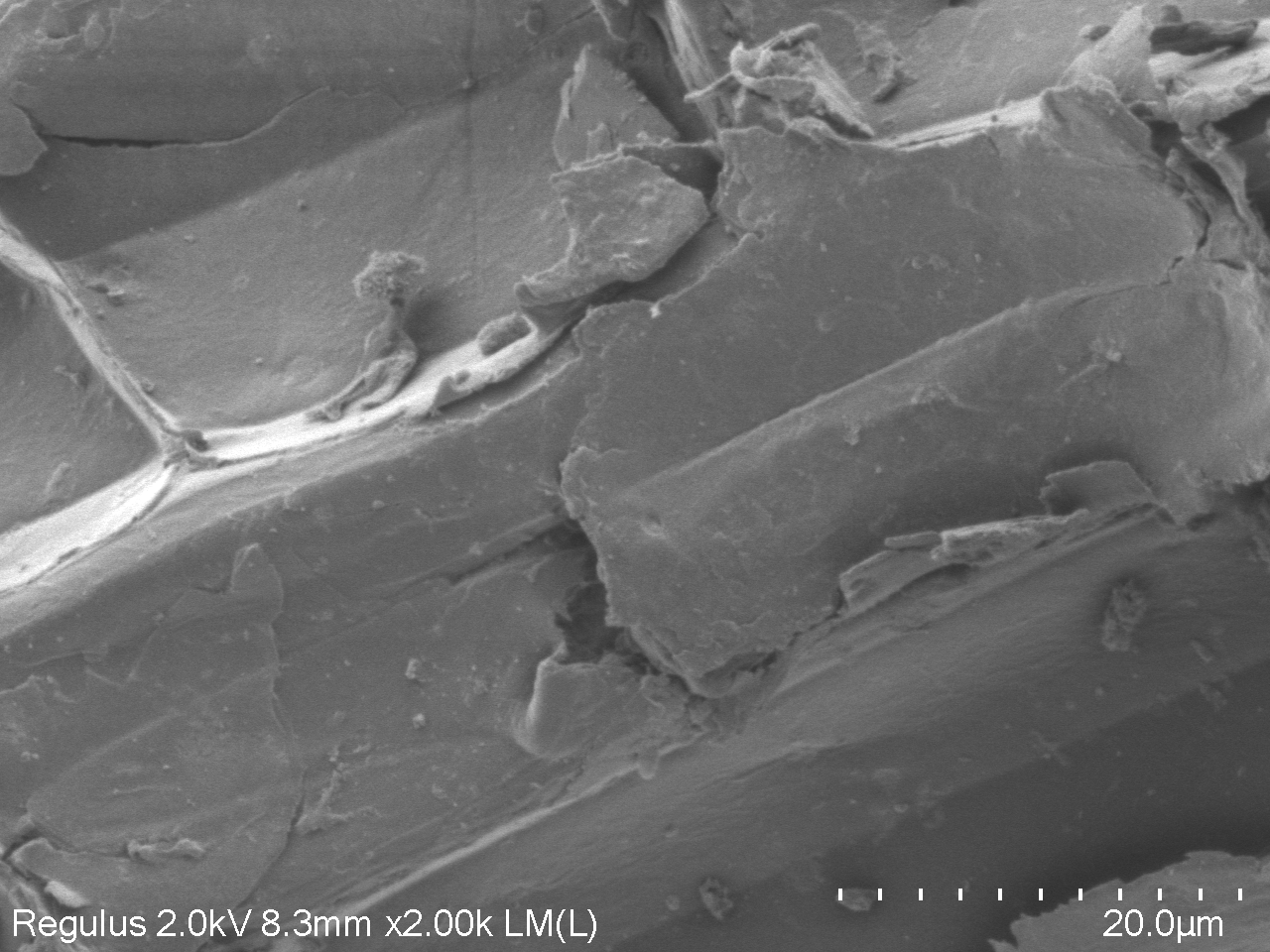

Supplement: Supplemental Information 6 [file peerj-13-20386-s006.zip › 3SEM1112/KB/1-1.tif]

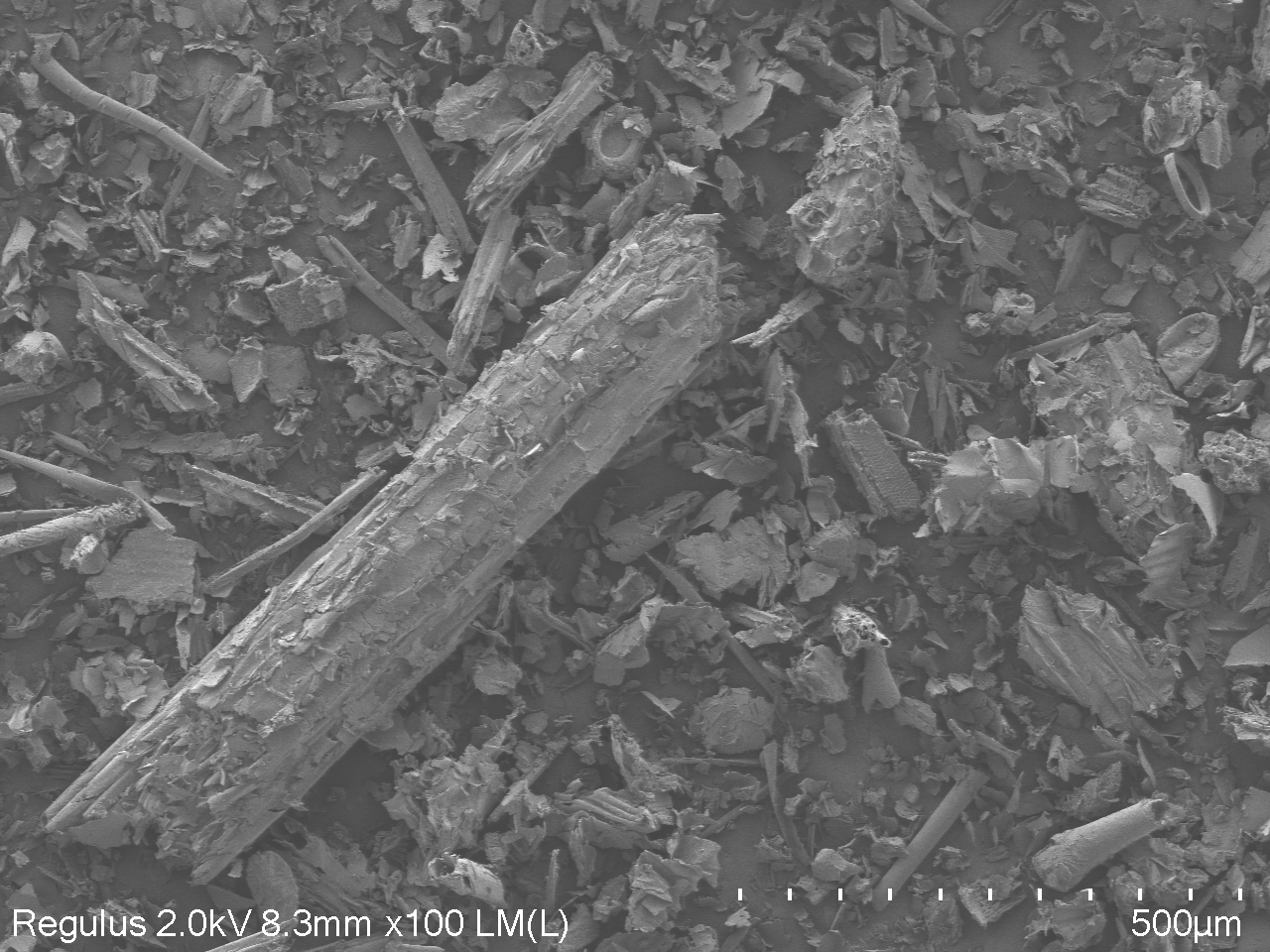

Supplement: Supplemental Information 6 [file peerj-13-20386-s006.zip › 3SEM1112/KB/1-10.tif]

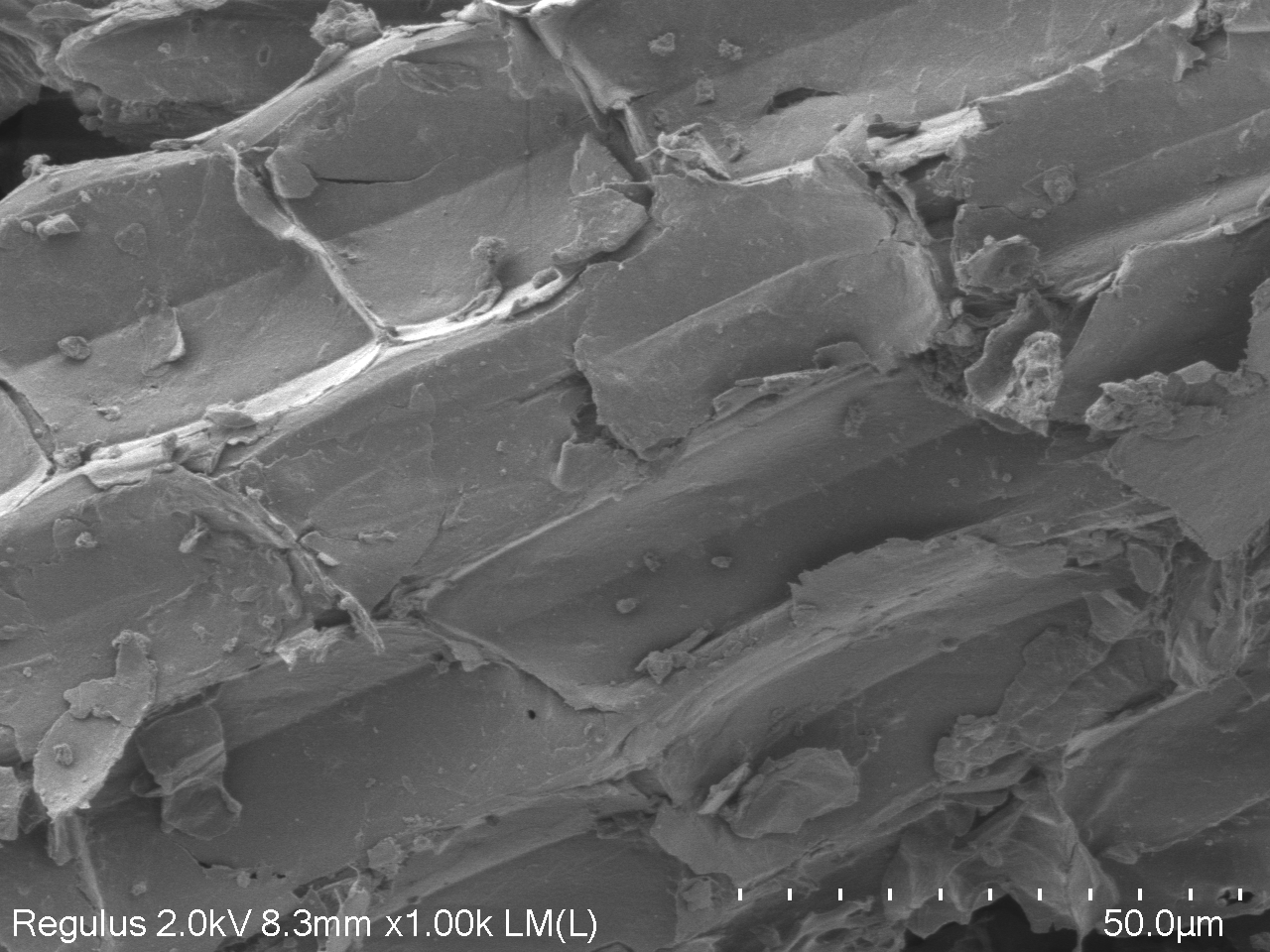

Supplement: Supplemental Information 6 [file peerj-13-20386-s006.zip › 3SEM1112/KB/1-2.tif]

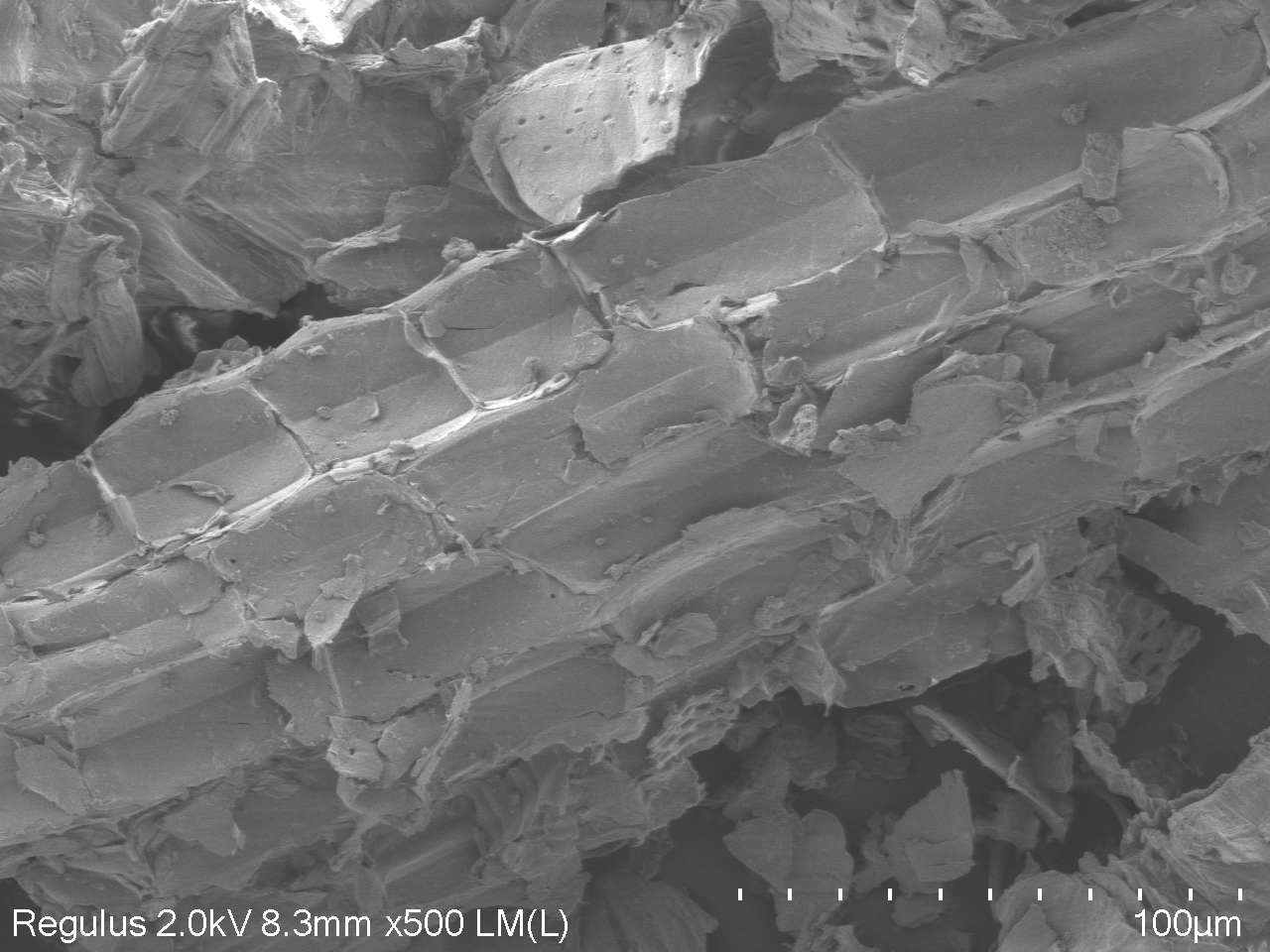

Supplement: Supplemental Information 6 [file peerj-13-20386-s006.zip › 3SEM1112/KB/1-3.tif]

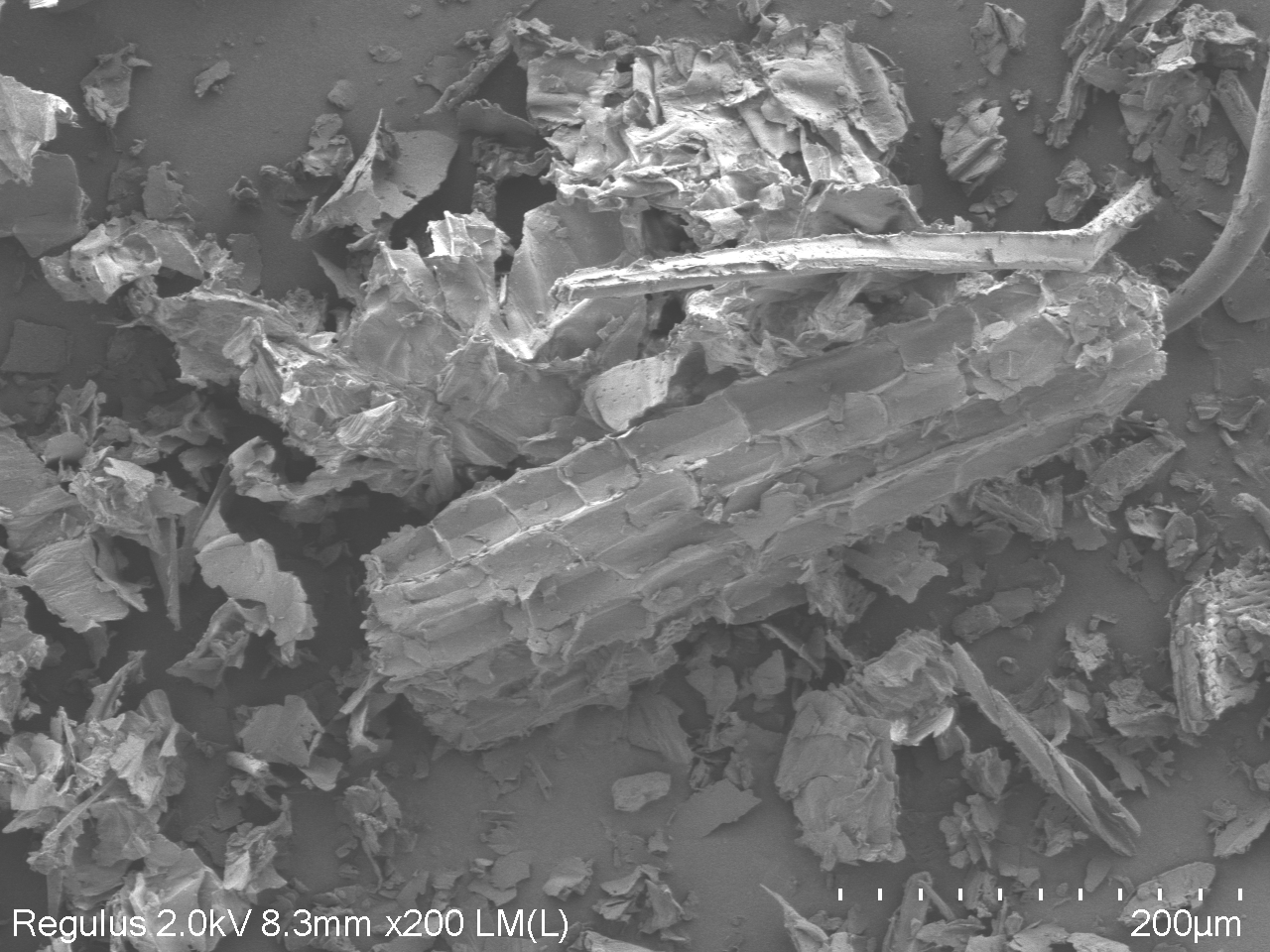

Supplement: Supplemental Information 6 [file peerj-13-20386-s006.zip › 3SEM1112/KB/1-4.tif]

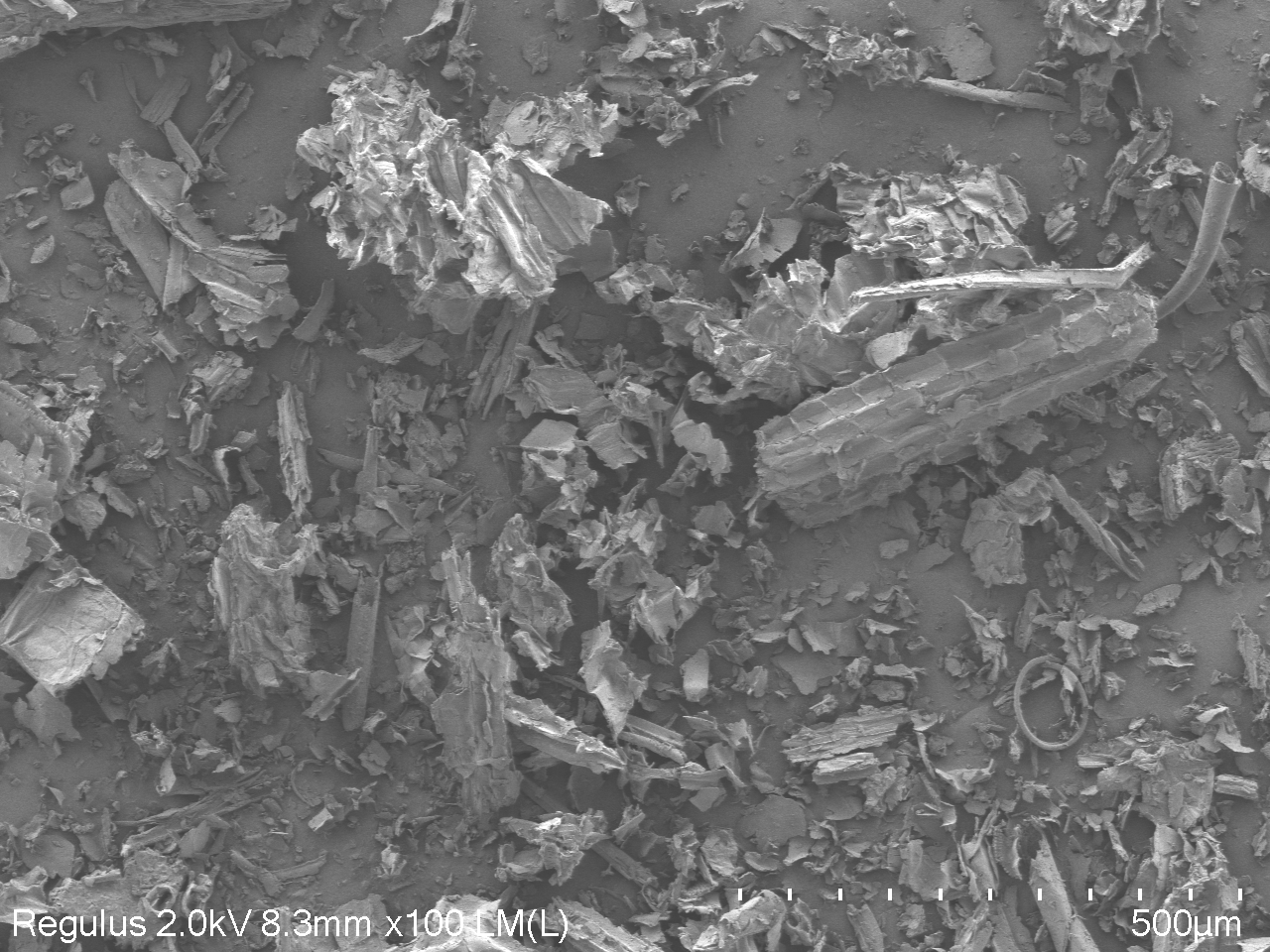

Supplement: Supplemental Information 6 [file peerj-13-20386-s006.zip › 3SEM1112/KB/1-5.tif]

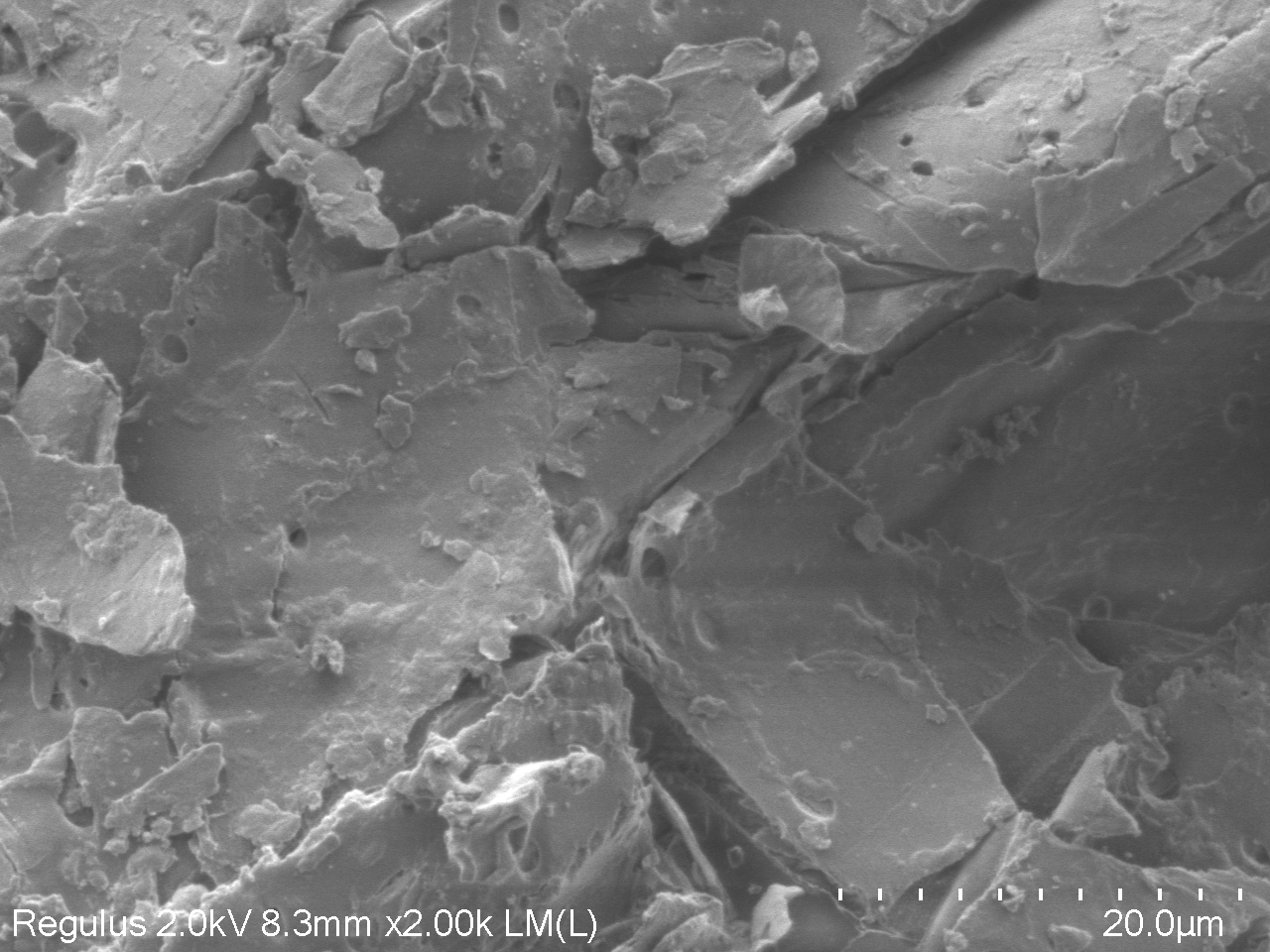

Supplement: Supplemental Information 6 [file peerj-13-20386-s006.zip › 3SEM1112/KB/1-6.tif]

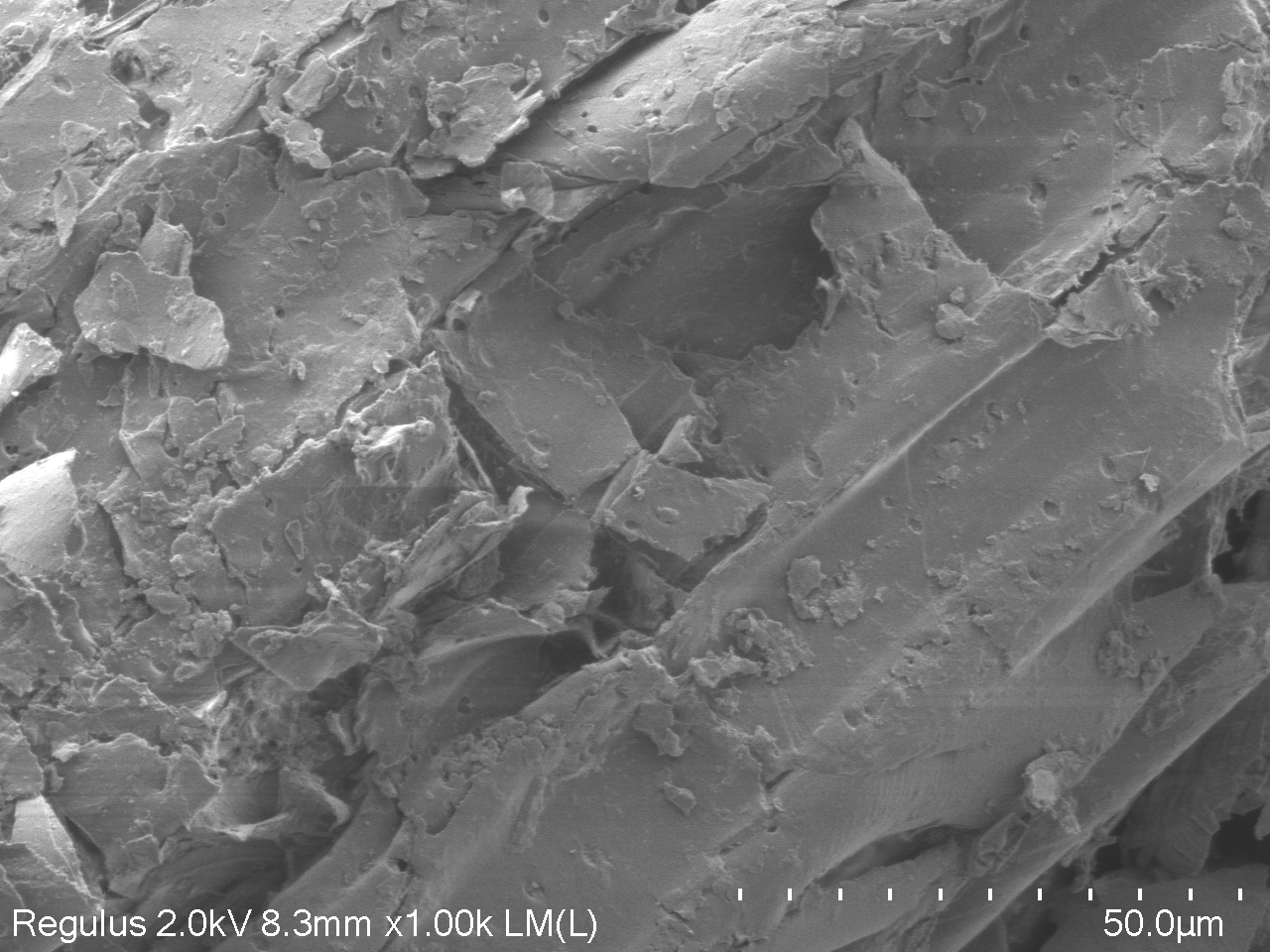

Supplement: Supplemental Information 6 [file peerj-13-20386-s006.zip › 3SEM1112/KB/1-7.tif]

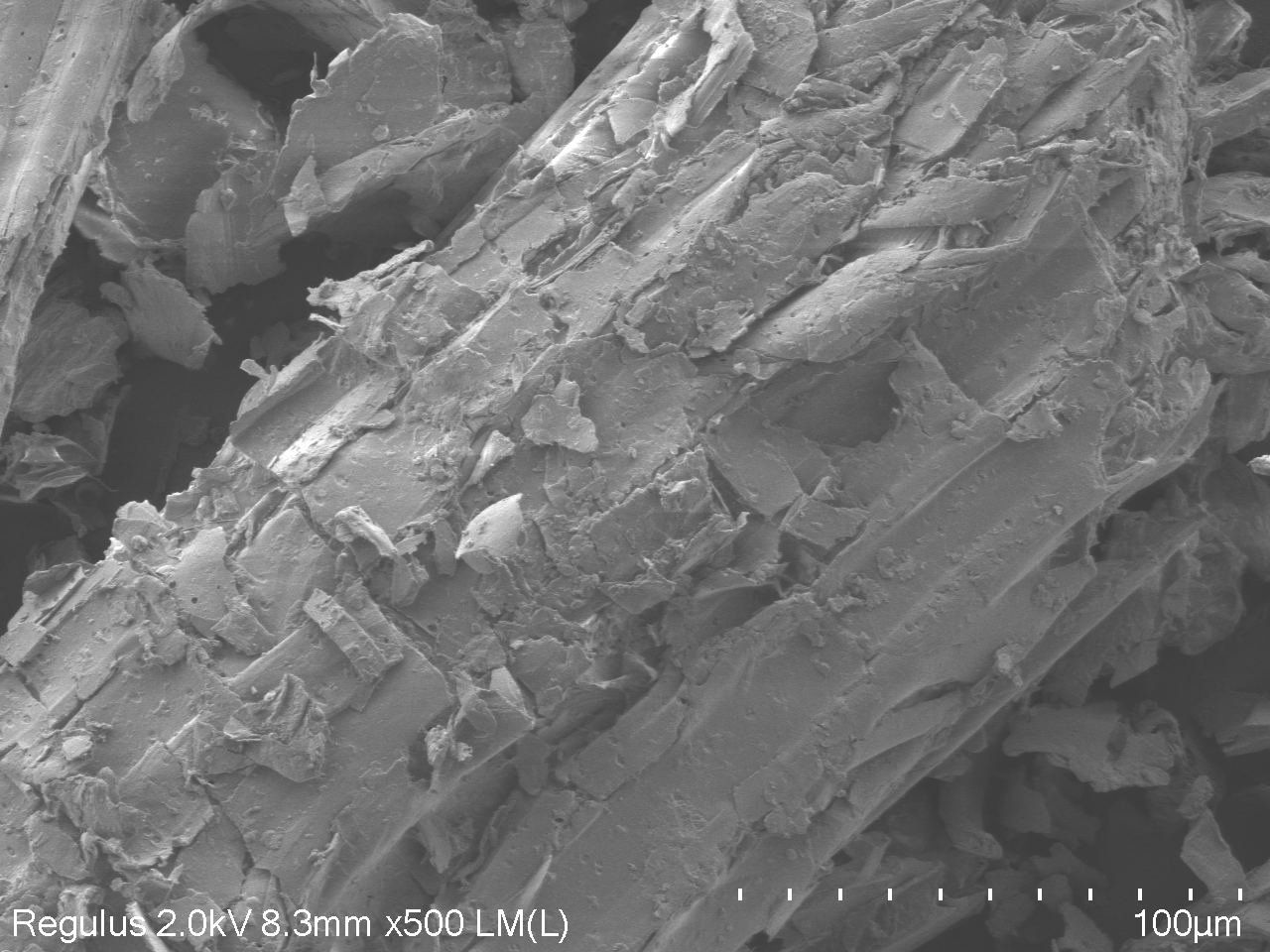

Supplement: Supplemental Information 6 [file peerj-13-20386-s006.zip › 3SEM1112/KB/1-8.tif]

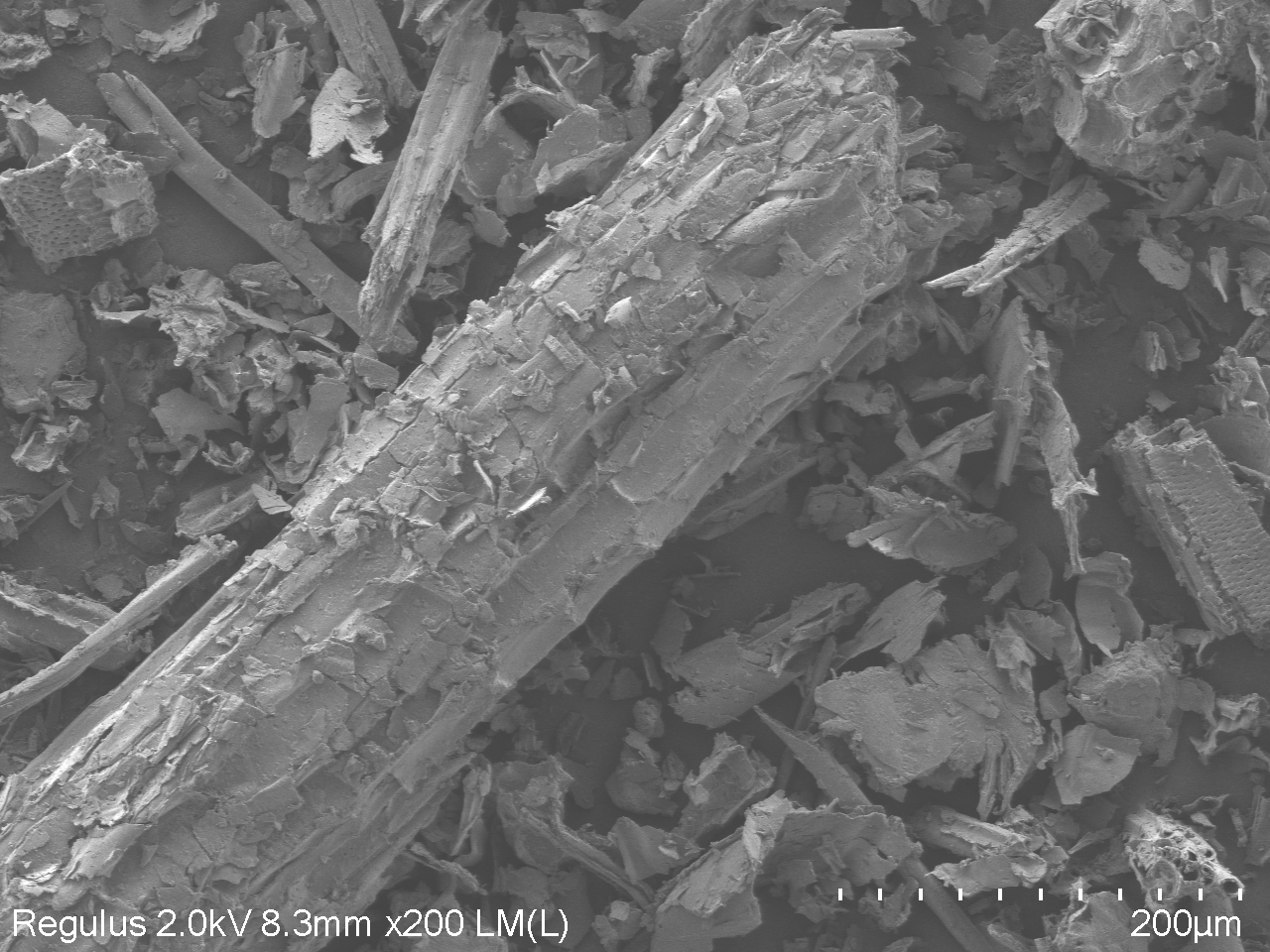

Supplement: Supplemental Information 6 [file peerj-13-20386-s006.zip › 3SEM1112/KB/1-9.tif]
