# Supplementary material for: Acclimation and degradation characteristic of the microbial system in corn straw
Source: PeerJ. 2025 Dec 16;13:e20386. doi: 10.7717/peerj.20386 (PMC12716131; doi:10.7717/peerj.20386)
Supplement: Supplemental Information 7 [file peerj-13-20386-s007.zip › Raw data 5FTIR observation/0.pdf]

Detailedinformationonthereport

Reportinglocation	D: Li Hongjia infrared sample view 1_Administrator 259. pdf
Report creator	Administrator
Report date	November 4, 2022, Friday, 11:05

Detailed information on the sample

Name of sample	Administrator259
Sample description	Sample 259 User Administrator Date Friday, November 04 2022
analyst	Administrator
creation date	2022-11-411:03:53
X-axis units	cm-1
Y-axis units	%T

Instrument details

Instrument model	SpectrumTwo
Instrument serial number	97951
software release	NIOS2Main00.02.006429-November-201310:09:27
Number of scans	1
resolution ratio	4

Instrument details (all)

Instrument model	SpectrumTwo
Instrument serial number	97951
software version	NIOS2Main00.02.006429-November-201310:09:27 1
Number of scans	1
resolution ratio	4
detector	MIRTGS
illuminant	MIR
light splitter	OptKBr
apodization	Stubborn
spectrum types	Light spectrum
beam type	Ratio
Correction of phase	Range
Scan speed	0.2
IGram Type	Two
scanning direction	Assemble
zero crossing	0
J-Stop aperture	8.94
IR-laser wave number	11750.00
manufacturer	L1600235
part number	L1600235
serial number	36926
instruction	ATRSamplebaseplateDiamond
The default scanning range is / cm-1	4000450
Force applied / N	30
Attachment type	UniversalATR
UATRcrystal combination	Diamond
UATR,number of rebounds	1
UATR options	Not specified

appendix

Manufacturer	L1600235
part number	L1600235
serial number	36926
instruction	ATRSamplebaseplateDiamond
The default scan range is / cm-1	4000450
Force applied / N	30
Attachment type	Universal
UATRcrystal combination	ATR,diamond
UATR,number of rebounds	1
UATR options	Not specified

PerkinElmer Spectrum Version 10.4.1 November 4, 2022
Friday 11:05

spectrogram

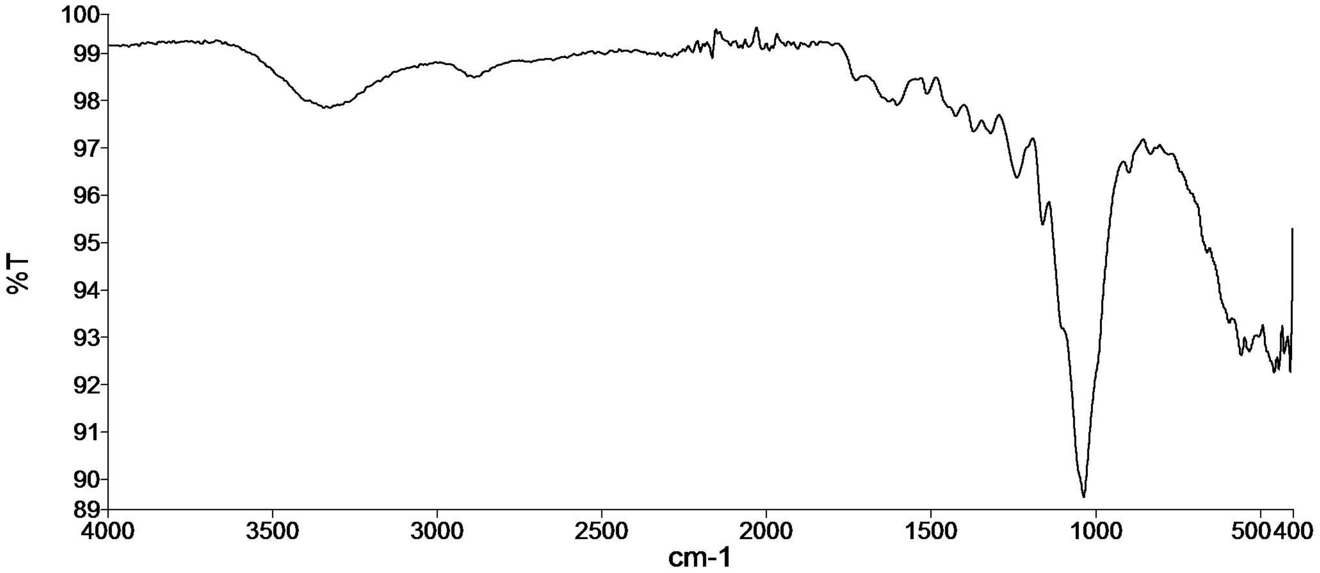

Name	Explain
Administrator 259	Sample 259 User Administrator Date Friday, November 04 2022
