# Supplementary material for: Acclimation and degradation characteristic of the microbial system in corn straw
Source: PeerJ. 2025 Dec 16;13:e20386. doi: 10.7717/peerj.20386 (PMC12716131; doi:10.7717/peerj.20386)
Supplement: Supplemental Information 7 [file peerj-13-20386-s007.zip › Raw data 5FTIR observation/3.pdf]

Detailed information on the report

Reportinglocation	D: Li Hongjia infrared sample view 1_ 260.pdf
Reportcreator	Administrator Administrator
Reportdate	November 4, 2022, Friday, 11:08

Detailed information on the sample

Name of sample	Administrator 260
Sample description	Sample 260 User Administrator Date Friday, November 04 2022
analyst	Administrator
creation date	2022-11-4 11:08:05
X-axis units	cm-1
Y-axis units	%T

Instrument details

Instrument model	Spectrum Two
Instrument serial number	97951
software release	NIOS2 Main 00.02.0064 29-November-2013 10:09:27
Number of scans	1
resolution ratio	4

Instrument details (all)

Instrument model	SpectrumTwo
Instrument serial number	97951
software version	NIOS2Main00.02.006429-November-201310:09:27
Number of scans	1
resolution ratio	4
detector	MIRTGS
illuminant	MIR
light splitter	OptKBr
apodization	Stubborn
spectrum types	Lightspectrum
beam type	Ratio
Correctionofphase	Range
Scanspeed	0.2
IGramType	Two
scanning direction	Assemble
zero crossing	0
J-Stop aperture	8.94
IR-laserwavenumber	11750.00
manufacturer	L1600235
part number	L1600235
serial number	36926
instruction	ATRSamplebaseplateDiamond
The default scanning range is/cm-1	4000450
Forceapplied/N	30
Attachmenttype	UniversalATR
UATRcrystalcombination	Diamond
UATR,numberofrebounds	1
UATRoptions	Notspecified

appendix

Manufacturer	L1600235
part number	L1600235
serial number	36926
instruction	ATR Sample base plate Diamond
The default scan rangeis/cm-1	4000 450
Forceapplied/N	30
Attachmenttype	UniversalATR
UATRcrystalcombination	diamond
UATR,numberofrebounds	1
UATRoptions	Not specified

spectrogram

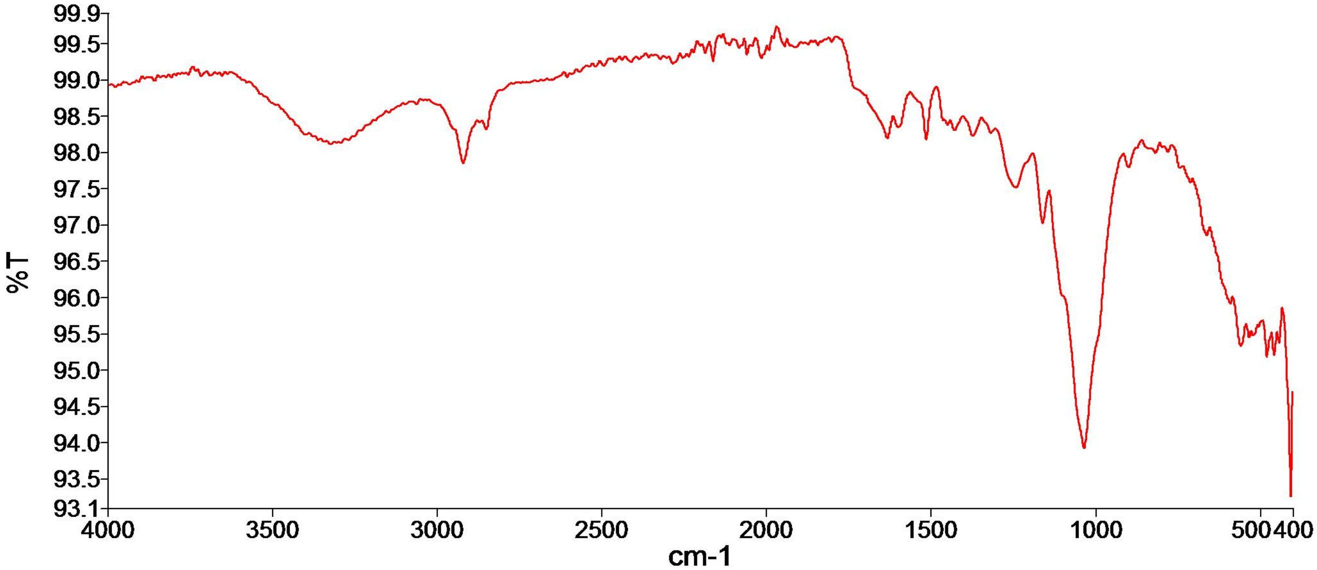

Name	Explain
Administrator 260	Sample 260 User Administrator Date Friday, November 04 2022
