# Supplementary material for: Acclimation and degradation characteristic of the microbial system in corn straw
Source: PeerJ. 2025 Dec 16;13:e20386. doi: 10.7717/peerj.20386 (PMC12716131; doi:10.7717/peerj.20386)
Supplement: Supplemental Information 7 [file peerj-13-20386-s007.zip › Raw data 5FTIR observation/5.pdf]

Detailed information on the report

Reportinglocation	D: Li Hongjia infrared sample view 1_ 261.pdf
Reportcreator	Administrator Administrator
Reportdate	November 4, 2022, Friday, 11:11

Detailed information on the sample

Name of sample	Administrator 261
Sample description	Sample 261 User Administrator Date Friday, November 04 2022
analyst	Administrator
creation date	2022-11-4 11:10:42
X-axis units	cm-1
Y-axis units	%T

Instrument details

Instrument model	Spectrum Two
Instrument serial number	97951
software release	NIOS2 Main 00.02.0064 29-November-2013 10:09:27
Number of scans	1
resolution ratio	4

Instrument details (all)

Instrument model	Spectrum Two
Instrument serial number	97951
software version	NIOS2 Main 00.02.0064 29-November-2013 10:09:27
Number of scans	1
resolution ratio	4
detector	MIR TGS
illuminant	MIR
light splitter	OptKBr
apodization	Stubborn
spectrum types	Light spectrum
beam type	Ratio
Correctionofphase	Range
Scanspeed	0.2
IGramType	Two
scanning direction	Assemble
zero crossing	0
J-Stop aperture	8.94
IR-laserwavenumber	11750.00
manufacturer	L1600235
partnumber	L1600235
serialnumber	36926
instruction	ATR Sample base plate Diamond
The default scanning range is / cm-1	4000 450
Forceapplied/N	30
Attachmenttype	Universal ATR
UATRcrystalcombination	Diamond
UATR,numberofrebounds	1
UATRoptions	Not specified

appendix

Manufacturer	L1600235
part number	L1600235
serial number	36926
instruction	ATR Sample base plate Diamond
The default scan rangeis/cm-1	4000 450
Forceapplied/N	30
Attachmenttype	Universal
UATRcrystalcombination	ATR, diamond
UATR,numberofrebounds	1
UATRoptions	Not specified

spectrogram

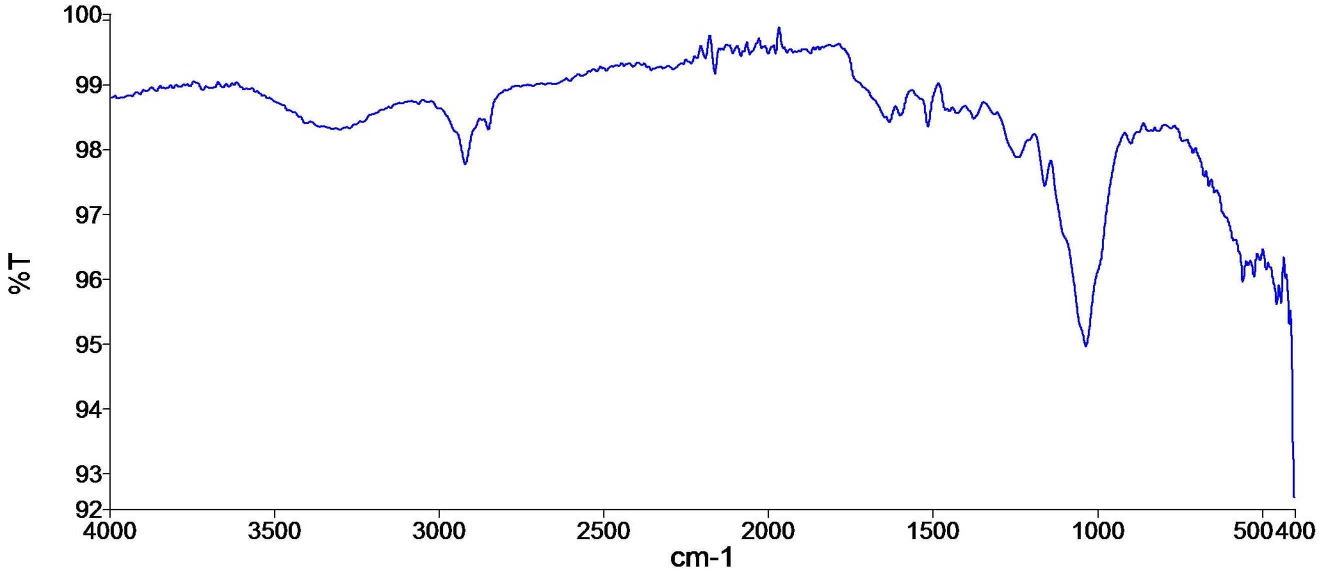

Name	Explain
Administrator 261	Sample 261 User Administrator Date Friday, November 04 2022
